# Deciphering the Role of RND Efflux Transporters in *Burkholderia cenocepacia*

**DOI:** 10.1371/journal.pone.0018902

**Published:** 2011-04-19

**Authors:** Silvia Bazzini, Claudia Udine, Andrea Sass, Maria Rosalia Pasca, Francesca Longo, Giovanni Emiliani, Marco Fondi, Elena Perrin, Francesca Decorosi, Carlo Viti, Luciana Giovannetti, Livia Leoni, Renato Fani, Giovanna Riccardi, Eshwar Mahenthiralingam, Silvia Buroni

**Affiliations:** 1 Dipartimento di Genetica e Microbiologia, Università degli Studi di Pavia, Pavia, Italy; 2 Cardiff School of Biosciences, Cardiff University, Cardiff, Wales, United Kingdom; 3 Dipartimento di Biologia, Università Roma Tre, Roma, Italy; 4 Trees and Timber Institute – National Research Council, San Michele all'Adige, Italy; 5 Department of Evolutionary Biology, University of Florence, Firenze, Italy; 6 Dipartimento di Biotecnologie Agrarie, Università degli Studi di Firenze, Firenze, Italy; University of Birmingham, United Kingdom

## Abstract

*Burkholderia cenocepacia* J2315 is representative of a highly problematic group of cystic fibrosis (CF) pathogens. Eradication of *B. cenocepacia* is very difficult with the antimicrobial therapy being ineffective due to its high resistance to clinically relevant antimicrobial agents and disinfectants. RND (Resistance-Nodulation-Cell Division) efflux pumps are known to be among the mediators of multidrug resistance in Gram-negative bacteria. Since the significance of the 16 RND efflux systems present in *B. cenocepacia* (named RND-1 to -16) has been only partially determined, the aim of this work was to analyze mutants of *B. cenocepacia* strain J2315 impaired in RND-4 and RND-9 efflux systems, and assess their role in the efflux of toxic compounds. The transcriptomes of mutants deleted individually in RND-4 and RND-9 (named D4 and D9), and a double-mutant in both efflux pumps (named D4-D9), were compared to that of the wild-type *B. cenocepacia* using microarray analysis. Microarray data were confirmed by qRT-PCR, phenotypic experiments, and by Phenotype MicroArray analysis. The data revealed that RND-4 made a significant contribution to the antibiotic resistance of *B. cenocepacia*, whereas RND-9 was only marginally involved in this process. Moreover, the double mutant D4-D9 showed a phenotype and an expression profile similar to D4. The microarray data showed that motility and chemotaxis-related genes appeared to be up-regulated in both D4 and D4–D9 strains. In contrast, these gene sets were down-regulated or expressed at levels similar to J2315 in the D9 mutant. Biofilm production was enhanced in all mutants. Overall, these results indicate that in *B. cenocepacia* RND pumps play a wider role than just in drug resistance, influencing additional phenotypic traits important for pathogenesis.

## Introduction

The *Burkholderia cepacia* complex (Bcc) constitutes a group of phenotypically similar non-fermenting, aerobic, Gram-negative rods that infect 2 to 8% of patients with cystic fibrosis (CF) [1]. Bcc comprises at least 17 different closely related species whose correct identification is particularly important in clinical microbiology as these bacteria are opportunistic pathogens that can cause severe lung infections in immuno-compromised as well as in CF patients [1].

In CF patients, antibiotics are used to clear early infection, treat acute exacerbations of chronic infection and reduce their relapse frequency. These treatments have had a major impact on the quality and survival of CF patients [2]. Despite the heavy use of antibiotics in CF, over the last decades, *B. cenocepacia* has emerged as an important respiratory pathogen in the CF community. Pulmonary colonization/infection by this bacterium may persist for months or even years but a minority of patients exhibits a rapid clinical deterioration associated with severe respiratory inflammation, epithelial necrosis and invasive disease, a condition known as cepacia syndrome [3], [4].

The *B. cenocepacia* epidemic ET12 lineage that originated in Canada and spread to Europe has been one of the most prevalent Bcc genotypes isolated from CF patients, with strain J2315 being studied in depth as model isolate [5]. The 8.06-Mb genome of this highly transmissible pathogen, consisting of three circular chromosomes and a plasmid, encodes a broad array of functions typical of metabolically versatile genus *Burkholderia*, as well as several virulence and drug resistance functions [5]. Antimicrobial therapy for Bcc is often ineffective as members of the *B. cepacia* complex are highly resistant to most clinically relevant antimicrobial agents and disinfectants [6]. Multi-drug resistance (MDR) in CF isolates is defined as resistance to all of the agents belonging to at least two of three classes of antibiotics, such as quinolones, aminoglycosides, and β-lactam agents, including monobactams and carbapenems [7].

Particularly interesting among mediators of MDR in Gram-negative bacteria are transporters belonging to the RND (Resistance-Nodulation-Cell Division) family, whose members catalyze the active efflux of many antibiotics and chemotherapeutic agents [8]. RND transporters are protein complexes that span both the cytoplasmic and outer membrane. The complex comprises a cytoplasmic membrane transporter protein, a periplasmic-exposed membrane adaptor protein, and an outer-membrane channel protein. The *Escherichia coli* AcrAB-TolC and the *Pseudomonas aeruginosa* MexAB-OprM complexes are well characterized; besides, the resolution of the three-dimensional structures of various components supported the model according to which these efflux systems form a channel for the extrusion of substrates/drugs from within the cell envelope back into the external environment [9]–[13]. There are also a number of studies suggesting that RND efflux systems play important roles in bacterial pathogenesis, participating in colonization and persistence of bacteria in the host, as well as in metal ion homeostasis [14], [15].

The significance of RND efflux systems in *B. cenocepacia* has been only partially determined. We have previously identified 14 genes encoding putative RND efflux pumps in the genome of *B. cenocepacia* J2315 [16]. After the completion of the whole genome sequence [5], two additional genes encoding RND pumps were discovered and, very recently, a complete description of the distribution of RND proteins within *Burkholderia* genus was obtained [17]. We named the operons encoding the *B. cenocepacia* RND efflux pumps RND-1 to RND-16 [18]. Most of these operons comprise the membrane fusion protein, the RND pump, and the outer membrane protein encoding genes.

Systematic measures of the role that RND efflux systems play in *Burkholderia* can be obtained by deleting single or multiple *rnd* operons and examining the genotype and phenotype of the resulting mutants. However, *B. cenocepacia* strain J2315 is difficult to manipulate genetically, in part due to its high level of antibiotic resistance, which precludes the use of the most common selectable markers for gene exchange. For this reason, also in our previous work, we adopted a recently developed mutagenesis strategy [19] to obtain *rnd* knockout mutants of *B. cenocepacia* J2315 [18]. The mutagenesis strategy we employed has the advantage of generating markerless deletions, making it possible to repeatedly use the same antibiotic resistance cassette for engineering subsequent gene deletions [19]. We successfully deleted three of these operons in *B. cenocepacia* strain J2315, encoding the putative RND-1, RND-3, and RND-4 transporters (namely BCAS0591*-*BCAS0593, BCAL1674*-*BCAL1676, and BCAL2822*-*BCAL2820 genes) and the corresponding inactivated strains were named D1, D3, and D4. The mutant phenotypes demonstrated that RND-3 and RND-4 contributed significantly to the antibiotic resistance of *B. cenocepacia* [18].

The availability of *rnd* knockout mutants in *B. cenocepacia* J2315 is a good starting point to further investigate the role of these efflux systems not only in antibiotic resistance but also in other metabolic pathways, including those relevant for pathogenesis. In fact, multidrug transporter genes are frequently subjected to both local and global regulation and are taking part in complex transcriptional networks, which may be elucidated by transcriptome analysis. Hence, the aim of this work was to analyze mutants of *B. cenocepacia* J2315 impaired in *rnd* genes to assess their role in the efflux of toxic compounds and physiology of *B. cenocepacia* by comparing the transcriptome of mutants with that of the wild-type strain using microarray analysis. We focused our attention on the previously characterized D4 strain, as it showed an interesting phenotype regarding drug resistance [18], and a novel mutant D9 [20], which was impaired in RND-9 operon (encoded by BCAM1945-1947 genes). We chose D9 since it has been recently shown by a combination of *in silico* analyses that BCAM1946 (RND-9) belongs to the HAE-1 family comprising proteins responsible for the extrusion of antibiotics [17], and thus might be able to pump out toxic compounds. However, the deep phylogenetic analysis performed by Perrin *et al.* [17] showed also that the BCAM1946 protein sequence joined the same cluster as BCAL2821 (RND-4), even if they belong to different and distant branches, and has a narrow phylogenetic distribution, in that its orthologs are present only in a few Bcc species. This finding suggests that RND-4 and RND-9 might be involved in different physiologic processes. Further, this operon was chosen as BCAM1947 gene was found to be over-expressed in the *sputum* of CF patients [21] and because the whole operon shares amino acid identity with the more known MexEF-OprN efflux system of *P. aeruginosa* [22], [23]. In fact, the product of BCAM1945 possesses a 38% amino acid sequence identity with OprN from *P. aeruginosa*, while BCAM1946 has a 56% of identity with MexF and BCAM1947 a 46% with MexE.

Hence, in this work we tried to shed some light on the role that RND-4 and RND-9 might have in cell physiology and in particular in the efflux of toxic compounds by analysing the transcriptome of three mutants: D4, which was previously described [18], D9 and D4–D9, single and double mutants respectively. Microarray data were confirmed by qRT-PCR and phenotypic experiments, as well as by Phenotype MicroArray analysis.

## Materials and Methods

### Bacterial strains and growth conditions

Bacterial strains and plasmids used in this study are listed in Table 1. Bacteria were grown in Luria-Bertani (LB) broth (Difco), with shaking at 200 rpm, or on LB agar, at 37°C. The construction of mutants D4, D9 and D4–D9 has been described in other papers [18], [20].

**Table 1:** Strains and plasmids used in this work.

Strain or plasmid	Relevant characteristics	Source and/or reference
***B. cenocepacia*** ** strains**		
J2315	CF clinical isolate	G. Manno
D4	J2315 ΔBCAL2820-BCAL2822	[18]
D9	J2315 ΔBCAM1945-BCAM1948	[20]
D4–D9	J2315 ΔBCAM1945-BCAM1948 ΔBCAL2820-BCAL2822	[20]
***E. coli*** ** strains**		
DH5α	F^−^ Φ80d*lacZ*Δ*M15* Δ(*lacZYA-argF*)*U169 endA1 recA1 hsdR*17(r_K_^−^ m_K_^+^) *supE44 thi*-*1* Δ*gyrA96 relA1*	Laboratory stock
SY327	*araD* Δ(*lac pro*) *argE*(Am) *recA56 nalA* λ *pir*, Rif^r^	M.A. Valvano
**Plasmids**		
pGEM-T Easy	Vector for PCR cloning, Amp^r^	Promega
pGPI*Sce*-I	*ori*_R6K_, ΩTp^r^, *mob* ^+^, containing the I*Sce*-I restriction site, Tp^r^	M.A. Valvano
pRK2013	*ori*_colE1_, RK2 derivative, Kan^r^, *mob* ^+^, *tra* ^+^, Kan^r^	M.A. Valvano
pDAI*Sce*-I	pDA12 encoding the I*Sce*-I homing endonuclease, Tet^r^	M.A. Valvano

Amp^r^, ampicillin resistance; Kan^r^, kanamycin resistance; Rif^r^, rifampin resistance; Tet^r^, tetracycline resistance; Tp^r^, trimethoprim resistance.

### MIC determination

Determination of MIC (Minimal Inhibitory Concentration) for *B. cenocepacia* J2315 and the deleted mutants D9 and D4–D9 was performed by streaking 1×10^4^ cells onto LB agar containing 2-fold dilutions of different drugs. The MIC was defined as the lowest drug concentration that prevented visible growth. The following compounds were tested to determine the resistance profile: aztreonam, ethidium bromide, chloramphenicol, gentamicin, tobramicin, nalidixic acid, ciprofloxacin, levofloxacin, norfloxacin, sparfloxacin, ampicillin, ceftazidime, erythromycin, meropenem, piperacillin, kanamycin and trimethoprim. Plates were incubated at 37°C for 3 days and growth was visually evaluated. The results represent the average of three independent replicates. The significance of MIC differences between the strains was assessed using the Wilcoxon rank-sum test.

### RNA purification and preparation for microarrays

For the microarray and qRT-PCR experiments, wild-type and mutant *B. cenocepacia* J2315 cells were harvested by centrifugation and transferred into sterile tubes. Total RNA was purified using the RiboPure Bacteria Kit (Ambion) according to the manufacturer's instructions. 1×10^9^ cells were used for three biological replicates of each strain (J2315, D4, D9 and D4–D9).

A 1 hour incubation of each sample with DNase I (Ambion) was used, following the manufacturer's instructions. After extraction, the RNA was concentrated using the LiCl method [24]. RNA quality and concentration were assessed using the Agilent 2100 Bioanalyzer (Agilent) and agarose gel electrophoresis. All RNA samples fulfilled the requirements for microarray experiments. 10 µg of total RNA were used for labeling reactions. cDNA generation and labeling was performed using the CyScribe Post-Labeling kit (GE Healthcare) according to the manufacturer's instructions and including spike-in controls for quality control (Agilent). cDNA was purified by ethanol precipitation and the purification of the labeled cDNA was performed using the CyScribe GFX purification kit. For the elution, water was used instead of the buffer provided by the kit. The quantification of the amount of generated cDNA and of Cy dye was performed with a NanoDrop spectrophotometer.

### Microarray hybridization and analysis

The microarrays used were 4×44 K 60-mer arrays that contain spots corresponding to all coding regions of *B. cenocepacia* J2315 genome (a total 7251 probes including duplicate probes for several genes) and also probes corresponding to selected intergenic regions (1489). Hybridization and washing were performed following the “Two-colour microarray based gene expression analysis” protocol from Agilent, with the following exceptions: fragmentation buffer was not used, 1 µl of a mixture of labeled oligonucleotides was added, and the mixture of cDNA was incubated at 98°C for 3 minutes for denaturing. The hybridization buffer was from the Gene Expression Hybridization kit. The microarrays were scanned using a microarray scanner (G2565 BA, Agilent) and the Scan Control software version A.7.0.3. Feb 2007 (Agilent). The scan region was adjusted to 61×21.6 mm and the scanning resolution was set to 5 µm. The Extended Dynamic Range function was switched on with 100% and 10% PMT gain settings. The images were analysed with the Feature Extraction software version 9.5.1. Feb 2007 (Agilent) and the FE protocol used was GE2_v5_95_Feb07 with default settings. GeneSpring was used to analyze gene expression data. The data were filtered based on expression level changes of greater than 1.5-fold. Differentially expressed genes were filtered on t-test p-value with a threshold of 0.05 (parametric test which does not assume the variances as equal: Welch's t-test) without multiple testing correction.

The software Blast2GO (version 2.3.4) [25] was used, with default parameters, to obtain the functional annotation of the differentially expressed transcripts as well as the related gene ontology (GO) terms. Blast2GO was also used for GO functional enrichment analysis of genes, by performing Fisher's exact test with robust false discovery rate (FDR) correction to obtain an adjusted p-value between certain test gene groups and the whole annotation.

### Quantitative Real-Time PCR (qRT-PCR)

For each strain six unlinked genes were chosen for qRT-PCR based on their differential expression pattern and annotation. Three genes were chosen among the up-regulated ones and three among the down-regulated ones. cDNA was synthesized using the M-MLV Reverse Transcriptase (Promega) and using 2 µg of total RNA as starting material. cDNA was precipitated, resuspended in DEPC water and stored at −80°C. Primer sequences for quantitative PCR are listed in Table 2. qRT-PCR reactions were performed on a Rotor-Gene-6000 cycler (Corbett), using QuantiFast SYBR Green PCR Kit (QIAGEN) according to the manufacturer's protocol except that 10 µl were used as a final volume for each reaction. Cycling conditions were: 95°C for 5 min (1 cycle), 95°C for 10 sec followed by 60°C for 30 sec (35 cycles). A melting curve analysis was included at the end of each run. Each sample was spotted in triplicate and a reference gene as well as control samples without cDNA were included in each experiment. The BCAM0166 (*ndh*) gene showed a stable expression in the different strains and was used as reference gene. The comparative Ct-method was used to determine the fold difference in gene expression between the mutant strains and the wild-type.

**Table 2:** Primers used in this work.

Primer name	Primer sequence
Bcal0114F	5′-CGGATGCAGACCCAGAT-3′
Bcal0114R	5′-TGCAGGCTGTTCGTCAG-3′
Bcal0135F	5′-AACATGCCGAACCTCG-3′
Bcal0135R	5′-GCGATGATGTTCTCCTT-3′
Bcal0140F	5′-GTGCCTTACCAACTCT-3′
Bcal0140R	5′-CTGCTGCTGGCGAATG-3′
Bcal0178F	5′-TTGGGCGACTCAATGG-3′
Bcal0178R	5′-TTCGTGTATGGCGGAT-3′
Bcal0520F	5′-CCTGCTTCCATCGCTT-3′
Bcal0520R	5′-ACGCTCAACCCGCCCG-3′
Bcal0566F	5′-TCGTACACCAACAGCG-3′
Bcal0566R	5′-TGAGCCCCACCGTCGT-3′
Bcal0577F	5′-GCAGGTCAGCAGCAAC-3′
Bcal0577R	5′-CTGCGCGTAAGCCTTCT-3′
Bcal1828F	5′-GCATCAGGCGGCTTAC-3′
Bcal1828R	5′-CGCTTCGTCGGGAAAC-3′
Bcal3152F	5′-CTGCTGACGCTGTTGC-3′
Bcal3152R	5′-AACTCCAGCCCGCCGAC-3′
Bcam0726F	5′-GCAGCATGAACCACAC-3′
Bcam0726R	5′-CTGGCAAAGACGAACC-3′
Bcam1484F	5′-AGCATCCCGATCAGGT-3′
Bcam1484R	5′-GGCGAAGCGGAAGACG-3′
Bcam2616F	5′-CTGCACGACCTGCTGG-3′
Bcam2616R	5′-TGCCGGTCTGCTCCTG-3′
Bcam0695F	5′-CGGGGCGAGCGGGTTG-3′
Bcam0695R	5′-CCTCGGCGGCGTCGTG-3′
Bcam0727F	5′-AGGTCGGCGGGCAGGA-3′
Bcam0727R	5′-GCGGTACAGGTGTTCG-3′
ndhF	5′-GCGATCGGGCTGTACAAGTT-3′
ndhR	5′-AGTGGCTCAGCGACTGGAA-3′

The comparison of gene expression fold change, obtained both by microarray analysis and qRT-PCR, was assessed by Pearson correlation.

### Swimming, biofilm and chemotaxis assays

For swimming assays, LB grown *B. cenocepacia* cultures (A_600__nm_ = 1.0) were inoculated with a toothpick on ‘swimming plates’ (1 g/l tryptone, 0.5 g/l yeast extract, 5 g/l NaCl, 3 g/l agar noble) and incubated for 42 hours at 37°C. In this growth medium bacteria can swim through the soft agar and produce a halo. The diameter of the halo is a measure of the ability to swim.

Crystal violet binding assay was carried out using 96-wells plates pre-treated with mucin as described by Rose *et al*. [26]. Bacterial cultures were grown in LB and diluted to A_600_ = 0.01; 150 µL of each strain was then placed into 96-well plates. The plates were incubated at 37°C statically for 72 hours. After incubation, the plates were washed three times with PBS to remove planktonic growth. The remaining biofilm was fixed with methanol for 15 min. Once methanol was removed and plates were dried, biofilms were stained with 1% Crystal Violet for 5 min. The stain was removed by washing with water and plates were dried. Biofilm thickness was measured by adding 33% glacial acetic acid and taking an OD reading at 600 nm using an automated plate reader [26].

The Congo red binding assay was carried out as previously described, with slight modifications [27]. Briefly, bacteria were grown on LB agar plates for 72 hours at 37°C. Colonies were scraped off, suspended in 9 g/l NaCl and normalized to a A_600__nm_ = 2.0. Cells from 1 ml of bacterial suspension were harvested by centrifugation, suspended in 1 ml of Congo red buffer [0.002% (w/v) Congo red dye (Sigma-Aldrich), 9 g/l NaCl] and incubated at room temperature for 10 min. Samples were then centrifuged for 5 min at 6000 rpm and the optical density at 500 nm wavelength (A_500__nm_) of the supernatant was measured. The amount of Congo red dye not retained by the cells was estimated by measuring the absorbance at A_500__nm_ of the supernatants. A_500__nm_ levels are in inverse proportion to exopolysaccharide and fimbrial structures production. The Congo red binding of *B. cenocepacia* J2315 is defined as one hundred percent binding.

For all the above described assays the average of the results obtained from three independent experiments are reported with standard deviation. The statistical significance of the observed differences in mean invasion frequencies was determined by calculating the p-values using the two-tailed Student t test for unpaired data sets. p-values are reported in figure legends.

The *Burkholderia* chemotaxis assay was slightly modified from Leungsakul *et al*., [28]. Cells in the exponential phase of growth or heat-killed cells (negative control) were washed and resuspended in drop assay medium (MSB containing 0.2% bacto-agar and 10 mM succinate as an energy source) and poured in Petri plates. 10 µl of 40% tryptone or 40 X LB or 20% yeast extract were poured at the centre of each plate. 10 µl of 20% casamino acids solution was used as a positive control. Heat-killed cells for negative controls were prepared by autoclaving at 121°C for 30 min (control for non-chemotactic aggregation). No-substrate negative controls were also used. The chemotactic response was assessed after 18 hours.

### Phenotype MicroArray (PM) tests

The four *B. cenocepacia* strains J2315, D4, D9 and D4–D9 were tested on chemical sensitivity PM panels (PM11–PM20) (Biolog) for 960 different conditions including several concentrations of a wide variety of antibiotics, antimetabolites, heavy metals and other inhibitors. A tetrazolium dye is used as a reporter of active metabolism [29]. The reduction of the dye causes the formation of a purple colour that is recorded by a CCD camera every 15 min and provides quantitative and kinetic information about the response of cells in the tested conditions.

The wild-type strain and the three mutants were grown 36 hours at 37°C on BUG agar (Biolog). A cellular suspension in IF-0 (Biolog), whose density was adjusted to 80% transmittance by a Biolog turbidimeter, was prepared for each strain. The cellular suspension was diluted 13.64 times in IF-10 GN/GP (Biolog), dye G (Biolog) was added, according to the Biolog instructions, and used for plate inoculation. All PM plates were incubated at 37°C in an Omnilog reader (Biolog). Readings were recorded for 48 hours and data were analysed with Omilog-PM software (release OM_PM_109 M) (Biolog).

The data from the Omnilog-PM software were filtered, using the area of the kinetic curves as a parameter, then transferred to Excel spreadsheets (Microsoft Corporation) and processed with Bionumerics software (Applied Math) for principal-component analysis (PCA) in order to establish the correlations between the phenotype profiles of the strains.

The Omnilog-PM software also allowed the IC50 value to be determined for each chemical tested (four concentrations of each chemical were present in the plates from PM11 to PM20). IC50 is expressed in well units and should be defined as the well or fraction of a well at which a particular per-well parameter (*i.e.* the area of the curve) is at half of its maximal value over a concentration series. The half-maximal value most likely falls between the per-well parametric values of two consecutive wells, in which case, a fraction of a well is interpolated from the half-maximal value (Biolog, personal communication).

### Microarray data accession numbers

The raw microarray data (J2315 and D4) can be found in ArrayExpress under the accession number E-MEXP-2999. The raw data (J2315, D9 and D4–D9) can be found in ArrayExpress under the accession number E-MEXP-2997.

## Results

### Resistance profile of *rnd* operon deleted mutants

In order to investigate the contribution of efflux pumps to intrinsic drug resistance of *B. cenocepacia* J2315, we recently deleted 3 operons encoding the putative RND transporters RND-1, RND-3, and RND-4 [18]. In this work we continued in the same direction and analyzed the effect of the deletion of operon encoding RND-9 efflux pump in the wild-type strain (D9 mutant), as well as in the D4 strain (the double D4-D9 mutant) [20]. RND-9, which is located on chromosome 2, comprises genes BCAM1945-1947. It is noteworthy that in the D9 and D4-D9 mutant strains the BCAM1948 gene, encoding a MerR transcriptional regulator and hypothesized to control the expression of RND-9 operon, was inactivated, too [20].

The strains D9 and D4-D9 were tested for their susceptibility to a number of drugs, in comparison to the wild-type strain *B. cenocepacia* J2315. Strain D9 showed a 2-fold decrease of the MIC value of aztreonam, ethidium bromide, tobramycin, levofloxacin, and sparfloxacin in respect to the wild-type strain (Table 3). The D4-D9 double mutant exhibited a 4 to 16-fold increase in drug susceptibility to several of the antimicrobials tested: in particular, it is more susceptible than the wild-type strain but comparable to the D4 mutant when exposed to aztreonam, chloramphenicol, ethidium bromide, gentamicin, tobramycin, and to different fluoroquinolones, such as nalidixic acid, ciprofloxacin, levofloxacin, norfloxacin, sparfloxacin. Furthermore, the MIC for nalidixic acid was 16-fold lower in D4–D9 than in J2315 and 4-fold lower than in D4 (Table 3). The MIC values of other drugs such as ampicillin, ceftazidime, meropenem, piperacillin, erythromycin, and kanamycin were not altered in the D9 and in the D4–D9 as compared to J2315 (data not shown).

**Table 3:** Antimicrobial susceptibilities (µg/ml) of *B. cenocepacia* J2315, D9 and D4-D9 mutant strains.

Compound	Strain			
	**J2315**	**D4**	**D9**	**D4**–**D9**
Aztreonam	2000	250	1000	250
Ethidium bromide	2000	125	1000	125
Chloramphenicol	4	1	4	1
Gentamicin	2000	1000	2000	1000
Tobramycin	1000	250	500	250
Nalidixic acid	16	4	16	1
Ciprofloxacin	8	2	8	2
Levofloxacin	4	0.5	2	0.5
Norfloxacin	32	8	32	8
Sparfloxacin	8	1	4	1

The significance of MIC differences between the strains was assessed using the Wilcoxon rank-sum test and all the differences in antimicrobial susceptibility were statistically significant (p<0.05).

### Transcriptome analysis

In order to derive more information about the biological role of RND transporters, transcriptome analysis was carried out by using *B. cenocepacia* J2315, D4, D9 and D4-D9 strains. After a global analysis of the microarray data obtained, different gene lists were generated: genes induced in each mutant *versus* wild-type J2315, down-regulated genes in each mutant strain *versus* J2315 and differentially expressed genes overlapping in different mutants. A complete list of the microarray data is reported in Table S1.

Overall, our analyses showed that 216 genes were differentially expressed in D4 mutant in respect to the wild-type strain (Table S1), corresponding to 3% of the total 7251 probes used in this work. Among them, 32 encoded hypothetical proteins with unknown function. 138 CDSs (64%) were up-regulated and 78 (36%) down-regulated (Table S1). 118 among the differentially expressed genes in D4 mutant were located on chromosome 1 (55%), 55 on chromosome 2 (25%), and 42 (20%) on chromosome 3. 60 intergenic regions appeared to be differentially expressed in D4 strain (39 up-regulated and 21 down-regulated, Table S1).

The D9 mutant showed 168 genes differentially expressed in respect to the wild-type strain (Table S1). Among them, 43 encoded proteins with unknown function and 1 was not annotated. 61 CDSs (36%) were up-regulated and 107 down-regulated (64%) (Table S1). 66 (40%) out of 168 differentially expressed genes were located on chromosome 1, 73 (43%) on chromosome 2 and 29 (17%) on chromosome 3. Moreover, also 26 intergenic regions resulted to be differentially expressed in this mutant (8 were up-regulated and 18 down-regulated, Table S1).

In the case of the D4–D9 strain, 550 differentially expressed genes (7.6% of the total probes) were detected. 257 of them (47% of the differentially expressed genes) resulted to be up-regulated, while 293 (53%) were down-regulated. 110 encoded proteins with unknown function and 1 was not annotated (Table S1). 259 out of 550 differentially expressed genes were located on chromosome 1 (47%), 221 (40%) on chromosome 2, 67 (12%) on chromosome 3, and 3 (less than 1%) on the plasmid. In the case of D4–D9 mutant, also 84 intergenic regions resulted to be differentially expressed (31 up-regulated and 53 down-regulated) respect to the wild-type strain.

A χ^2^ analysis of the distribution of the differentially expressed genes on each chromosome was performed. In the case of D4 and D9 strains, the results indicate that there is a significant correlation between the number of differentially expressed genes and their location on each chromosome, *i.e.* the proportion of differentially expressed genes of each chromosome is correlating to the total number of genes on each chromosome. This was not observed with the D4-D9 double mutant.

**Figure 1:**
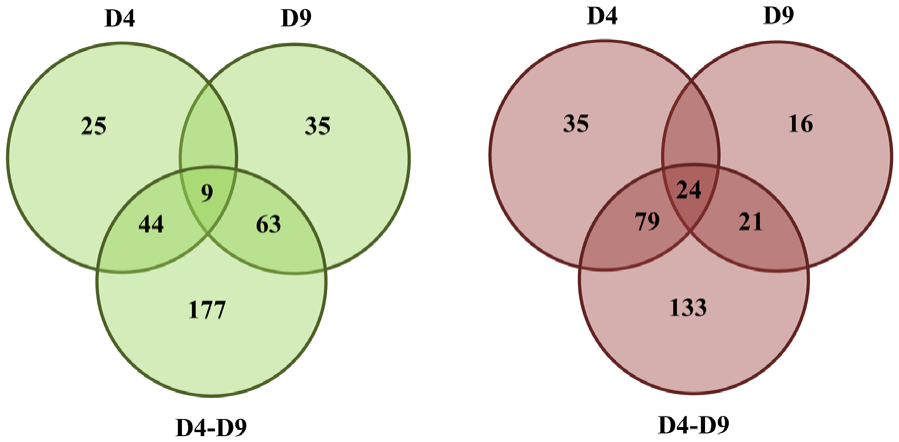
Differential gene regulation in the *B. cenocepacia* RND efflux mutants. The Venn diagram represents the differently expressed genes (down-regulated on the left, up-regulated on the right) in each mutant with respect to the wild-type strain.

Among the differentially expressed genes, it was possible to find some similarities shared by our efflux pump deleted strains (Figure 1). In particular, 33 genes resulted to be differentially expressed with respect to the J2315 strain in all the mutants described in this work (Table S1), with 24 of them being up-regulated in all mutants, and 9 down-regulated (Figure 1). 84 genes were differentially expressed in both D9 and D4–D9 mutants; interestingly, only BCAM1697 resulted to be up-regulated in D9 and down-regulated in the double mutant, while the expression profile of all the other genes was consistent in both strains, 63 being down-regulated and 21 up-regulated (Table S1, Figure 1). As regarding D4 and D4-D9 mutants, 123 genes resulted to be differentially expressed in both (44 down-regulated and 79 up-regulated, all consistent in both strains, Table S1). These concepts are clarified by the Venn diagram shown in Figure 1.

Major classes of *B. cenocepacia* genes with altered expression in the mutant strains in respect to the wild-type were identified (see supplemental files: Table S1 and Figures S1, S2, S3, S4, S5, S6). Using a functional enrichment analysis of genes (see supplemental files: Tables S2, S3, S4, S5, S6, S7) it was possible to individuate statistically significant functional categories that are over- or under- represented in the differentially expressed gene-lists of the efflux pump(s)-deleted mutant strains described in this work in respect to the wild-type. The composition of these gene groups is discussed in more detail below.

### Flagellum mediated motility

Like many other microorganisms, *B. cepacia* complex bacteria are motile and use complex protein structures called flagella. They possess one or longer polar flagella responsible for swimming motility. Many biological processes other than motility require the presence of these structures, for example the production of biofilms, adherence and invasion into host cells [30]–[32]. Flagella represent one of the virulence factors which contribute to the development of disease caused by these bacteria as shown by *in vivo* data [33]. They have been described as a major factor contributing to host inflammatory responses to bacteria due to the interaction of bacterial flagellin with the Toll-like receptor 5 (TLR5) [34]–[36].

The production and assembly of these multi-component structures involve more than 40 genes. In particular, members of the Bcc express one of two types of flagellin that can be distinguished by size (55 kDa for type I and 45 kDa for type II) and restriction fragment length polymorphism (RFLP) patterns of the *fliC* gene [37], [38].

The results obtained from the comparison of the microarray analysis of *B. cenocepacia* J2315 with D4, D9 and D4–D9 mutants showed that a large proportion of the differentially expressed genes were involved in flagellum assembly and motility (Table S1, Figures S1, S4 and S5). In particular, D4 and D4-D9 mutants shared 26 up-regulated flagellum-related genes (Table 4). Among them we found: *fliC* (BCAL0114), encoding the major structural component of flagellin, and BCAL0521 encoding the flagellar protein FliJ; BCAL0140-BCAL0143, BCAL0523, BCAL0527, BCAL0561, and BCAL3501, which code for flagellar biosynthesis proteins and assembly; BCAL0113, BCAL0520, BCAL0567 and BCAL0577 encoding the hook-associated proteins. Moreover, some genes belonging to the flagellar regulon master regulator *flh* (BCAL0124 and BCAL0125) were also over-expressed in these mutants (Table 4). Lastly, some flagellar basal body Rod protein encoding genes (BCAL0565, BCAL0566, BCAL0568, BCAL0569, and BCAL3507) were up-regulated and shared by the two mutants, as like as P- and L-ring proteins encoding ones (BCAL0570 and BCAL0571) (Table 4).

**Table 4:** Motility and adherence related genes differentially expressed in *B. cenocepacia* D4, D9 and D4–D9 mutants respect to J2315.

Gene	Description	Change in gene expression(log_2_ fold change)		
		D4 *vs* J2315	D9 *vs* J2315	D4–D9 *vs* J2315
BCAL0113	flagellar hook-associated protein	4.89	-	3.75
BCAL0114	flagellin	7.76	-	4.97
BCAL0124	flagellar regulon master regulator subunit FlhD	3.59	-	1.52
BCAL0125	flagellar regulon master regulator subunit FlhC	3.31	−1.16	2.03
BCAL0140	flagellar biosynthetic protein FlhB	3.78	−1.88	2.63
BCAL0142	flagellar biosynthesis protein FlhF	3.25	−0.82	2.28
BCAL0143	flagellar biosynthesis protein FlhG	4.53	-	1.85
BCAL0144	RNA polymerase sigma factor for flagellar	2.52	-	1.05
BCAL0520	putative flagellar hook-length control protein	2.98	−1.21	2.31
BCAL0521	flagellar fliJ protein	3.23	-	1.88
BCAL0522	flagellum-specific ATP synthase	3.55	−1.85	2.36
BCAL0523	flagellar assembly protein	3.73	-	2.16
BCAL0524	flagellar motor switch protein	2.03	-	-
BCAL0525	flagellar M-ring protein	2.16	-	-
BCAL0526	fliE flagellar hook-basal body complex protein FliE	2.19	-	-
BCAL0527	flagellar protein	3.243	-	2.89
BCAL0561	putative flagella synthesis protein	2.23	-	1.38
BCAL0562	putative negative regulator of flagellin	2.81	-	1.34
BCAL0564	putative flagellar basal-body Rod protein	3.44	-	-
BCAL0565	flagellar basal-body Rod protein	3.23	-	1.90
BCAL0566	putative basal-body Rod modification protein	4.88	−1.21	2.56
BCAL0567	putative flagellar hook protein	4.18	−1.31	2.36
BCAL0568	flagellar basal-body Rod protein	4.02	−1.34	2.43
BCAL0569	flagellar basal-body Rod protein	4.10	-	2.35
BCAL0570	flagellar L-ring protein precursor	3.14	−1.21	1.94
BCAL0571	flagellar P-ring protein precuror	2.85	−0.61	1.83
BCAL0576	putative flagellar hook-associated protein	4.41	-	-
BCAL0577	putative flagellar hook-associated protein	4.30	-	4.00
BCAL3501	flagellar biosynthetic protein	2.71	-	1.47
BCAL3503	flagellar biosynthetic protein	1.07	-	-
BCAL3505	probable flagellar motor switch protein	3.13	-	1.83
BCAL3506	flagellar motor switch protein FliM	2.58	-	-
BCAL3507	flagellar basal body-associated protein FliL	1.68	-	1.28
BCAM0777	putative flagellar motor proton channel	1.38	-	-
BCAM0778	putative flagellar motor protein	1.73	-	-
BCAM0987	putative flagellar basal body Rod protein	1.84	-	-
BCAM2758	cblS, two-component regulatory system. sensor kinase protein	1.33	-	-
BCAM2759	cblD, putative minor pilin and initiator	1.58	-	-

In addition to the genes mentioned above, D4 mutant over-expressed 12 additional flagellum-associated genes, four of which coding for flagellar motor proteins (BCAL0524, BCAL3506, BCAM0777, and BCAM0778) and other two for flagellar basal body Rod protein (BCAL0564 and BCAM0987) (Table 4).

In contrast, the D9 mutant showed an enrichment of motility related genes in the down-regulated gene list (BCAL0125, BCAL0140, BCAL0142, BCAL0520, BCAL0522, BCAL0566, BCAL0567, BCAL0568, BCAL0570, BCAL0571) (Table 4; Figure S4).

### Chemotaxis

The bacterial chemotaxis, which is mediated by two-component systems, directs motile cells to favourable environments by controlling phosphorylation of histidine kinase CheA and its cognate response regulator CheY. Kinase activity is modulated by the chemoreceptors, which are in turn regulated by both the binding of chemoeffector and the level of methylation [39]–[41].

The expression of chemotaxis-related genes was strongly influenced by the inactivation of the RND pumps. The D4 and D4-D9 mutants shared 13 up-regulated chemotaxis-related genes with respect to *B. cenocepacia* J2315 (Table 5). These genes encoded MotA and MotB chemotaxis proteins (BCAL0126 and BCAL0127), the chemotaxis two-component sensor regulator (BCAL0128), and the sensor kinase CheA (BCAL0129). Moreover, the CheY2 (BCAL0135) and the CheZ (BCAL0136) encoding genes were up-regulated in both deleted strains, as well as other four genes coding for methyl-accepting chemotaxis proteins (BCAL0762, BCAM1424, BCAM1804, and BCAM2689), plus one methyl-transferase (BCAL0132), and one methyl-esterase (BCAL0134) (Table 5).

**Table 5:** Chemotaxis related genes differentially expressed in *B. cenocepacia* D4, D9 and D4-D9 mutants respect to J2315.

Gene	Description	Change in gene expression(log_2_ fold change)		
		D4 vs J2315	D9 vs J2315	D4-D9 vs J2315
BCAL0126	chemotaxis protein MotA	3.43	-	2.27
BCAL0127	chemotaxis protein MotB	3.09	-	2.02
BCAL0128	chemotaxis two-component response regulator	3.32	-	2.44
BCAL0129	chemotaxis two-component sensor kinase CheA	3.52	-1.45	1.91
BCAL0130	chemotaxis protein CheW	2.99	-	-
BCAL0131	methyl-accepting chemotaxis protein I	1.48	-	-
BCAL0132	chemotaxis protein methyltransferase	3.48	-	1.49
BCAL0133	putative chemotaxis protein	3.33	-1.36	1.86
BCAL0134	chemotaxis protein-glutamate methylesterase	3.19	-0.83	1.73
BCAL0135	chemotaxis protein CheY2	2.55	-	1.34
BCAL0136	chemotaxis protein CheZ	2.48	-0.62	1.46
BCAL0762	putative methyl-accepting chemotaxis protein	1.96	-	1.58
BCAL1452	putative chemotaxis methyl-accepting membrane	-	-	0.69
BCAM1424	methyl-accepting chemotaxis protein	3.56	-	3.44
BCAM1503	putative methyl-accepting chemotaxis protein	1.87	-	-
BCAM1804	methyl-accepting chemotaxis protein	3.29	-	2.93
BCAM2374	putative methyl-accepting chemotaxis protein	1.45	-	-
BCAM2689	putative methyl-accepting chemotaxis protein	1.19	-	0.92

Furthermore, the D4 strain over-expressed other methyl-accepting chemotaxis proteins: (BCAL0131, BCAM1503, and BCAM2374), and BCAL0130 coding for the chemotaxis protein CheW. D4–D9 strain also over-expressed BCAL1452, coding for a methyl-accepting chemotaxis protein (Table 5). In contrast, in the D9 mutant no chemotaxis-related genes were over-expressed, while several of them were down-regulated (BCAL0129, BCAL0133, BCAL0134, BCAL0136) (Table 5; Figure S4).

### Down-regulated genes

The genes that showed a decreased expression profile in D4 and D4–D9 mutants belonged to many different functional classes. It was not possible to observe particularly representative classes because only a small number of the down-regulated genes were associated to each of many different metabolic processes. The under-expressed genes were mainly involved in basal metabolic processes of the cells, such as: macromolecule metabolic process, biopolymer modification, regulation of biosynthetic processes, regulation of cellular metabolic processes, cellular respiration and protein transport (Table S1, Figure S2 and S6). Strikingly, the down-regulated genes in mutant D9 belonged both to the motility/adherence and chemotaxis classes in contrast to the D4 and D4-D9 mutants which up-regulated this class of genes (Table S1, Figure S3). It is quite possible that the phenotype exhibited by the double mutant might be linked to D4 inactivation.

### Verification of microarray data by qRT-PCR

Evaluation of the fold change correlation between qRT-PCR experiments and microarray analysis was used to validate the over-expression and under-expression ratios observed in the microarray data (Table 6). 6 genes for each strain were chosen on the basis of their putative function, expression patterns and statistical reliability of the expression fold-change. The primers used are listed in Table 2.

**Table 6:** Fold change obtained in D4, D9 and D4–D9 microarray compared to the fold change obtained by qRT-PCR.

Gene	Description	Microarraylog_2_ fold change	qRT-PCRlog_2_ fold change
		**D4**	
BCAL0114	flagellin	7.76	7.47
BCAL0135	chemotaxis protein CheY2	2.55	0.62
BCAL0577	putative flagellar hook-associated protein	4.31	6.54
BCAL0178	putative DNA methyltransferase	−3.11	−10.45
BCAL3152	putative RNA polymerase sigma factor	−4.37	−2.23
BCAM2616	putative HTH AraC family transcriptional regulator	−1.97	−0.48
		**D9**	
BCAM0726	conserved hypothetical protein	1.71	2.38
BCAM0727	conserved hypothetical protein	1.24	2.93
BCAM1484	putative response regulator	0.58	0.19
BCAL0140	flagellar biosynthetic protein FlhB	−1.88	−1.14
BCAL0520	putative flagellar hook-length control protein	−1.21	−3.19
BCAL0566	putative basal-body Rod modification protein	−1.21	−2.86
		**D4–D9**	
BCAL0140	flagellar biosynthetic protein FlhB	2.63	2.28
BCAL0520	putative flagellar hook-length control protein	2.31	1.84
BCAL0566	putative basal-body Rod modification protein	2.56	3.03
BCAL1828	putative fimbrial usher protein	−5.25	−3.90
BCAL3152	putative RNA polymerase sigma factor	−3.96	−3.85
BCAM0695	putative lipoprotein	−6.75	−4.56

For the D4 mutant strain the genes coding for the following proteins were chosen: the flagellin (BCAL0114), the chemotaxis protein CheY2 (BCAL0135), the putative DNA methyltransferase (BCAL0178), the putative flagellar hook-associated protein (BCAL0577), the putative RNA polymerase sigma factor (BCAL3152), and the putative HTH AraC family transcriptional regulator (BCAM2616). For the D9 mutant the following were selected: the flagellar biosynthetic protein FlhB (BCAL0140), the putative flagellar hook-length control protein (BCAL0520), the putative basal-body Rod modification protein (BCAL0566), two conserved hypothetical proteins (BCAM0726 and BCAM0727), and the putative response regulator BCAM1484 encoding genes. For the double mutant D4–D9 the following genes were chosen: BCAL0140, BCAL0520, BCAL0566, BCAL3152, the putative fimbrial usher protein encoding gene (BCAL1828), and the putative lipoprotein encoding gene BCAM0695. BCAM0166 (*ndh*, NADH dehydrogenase encoding gene) was used as internal reference gene. Over-expression and under-expression ratios were statistically consistent with the microarray findings and the overall trend of gene expression was similar for both microarray and qRT-PCR experiments for all the tested genes (Table 6), as shown by Pearson correlation (data not shown). A good linear correlation between both datasets was observed, with a coefficient of 0.85 (p<0.01) and a slope of 0.72.

### Involvement of RND-4 and RND-9 efflux pumps in flagella-dependent phenotypes

Microarray analysis suggested that the RND-4 and RND-9 efflux pumps could play opposite roles in flagellum-dependent functions, like swimming and chemotaxis. To assess this hypothesis, these phenotypes were analyzed in the wild-type and in the RND-mutated strains, as described in Materials and Methods.

**Figure 2:**
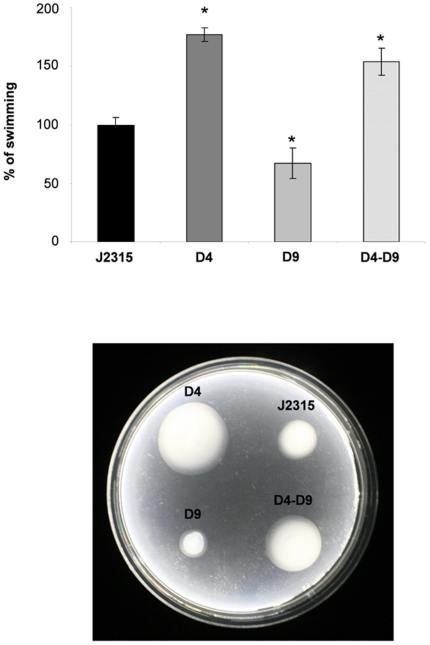
Effect of RND-4 and RND-9 mutations on swimming motility. The average diameter of swimming halos from three different experiments are plotted with standard deviations. Significantly differences with respect to J2315 are indicated by an * (p<0.01). Results are given in percentage, considering *B. cenocepacia* J2315 (wt) swimming halo as 100%. The panel below the graph shows one representative experiment. J2315, *B. cenocepacia* wild-type; D4, RND-4 mutant; D9, RND-9 mutant; D4-D9, RND4-RND9 mutant.

Data obtained revealed that single mutants D4 and D9 showed enhanced and reduced swimming motility with respect to the wild-type, respectively. Moreover, the D4–D9 mutant showed a swimming phenotype similar to that of the D4 mutant, suggesting that at least for this phenotype, mutation of RND-4 dominates over the mutation of RND-9 (Figure 2). These data were in full agreement with the microarray analysis, showing that flagellum-related genes are up-regulated in the D4 and D4–D9 mutants and down-regulated in the D9 mutant (Table 4).

Concerning chemotaxis, we have performed preliminary experiments using different attractant/repellents. The three mutants and the wild-type showed the same positive chemotactic phenotype versus casaminoacids and LB, and absence of chemotactic response using toluene, aztreonam and chloramphenicol as repellents (data not shown).

**Figure 3:**
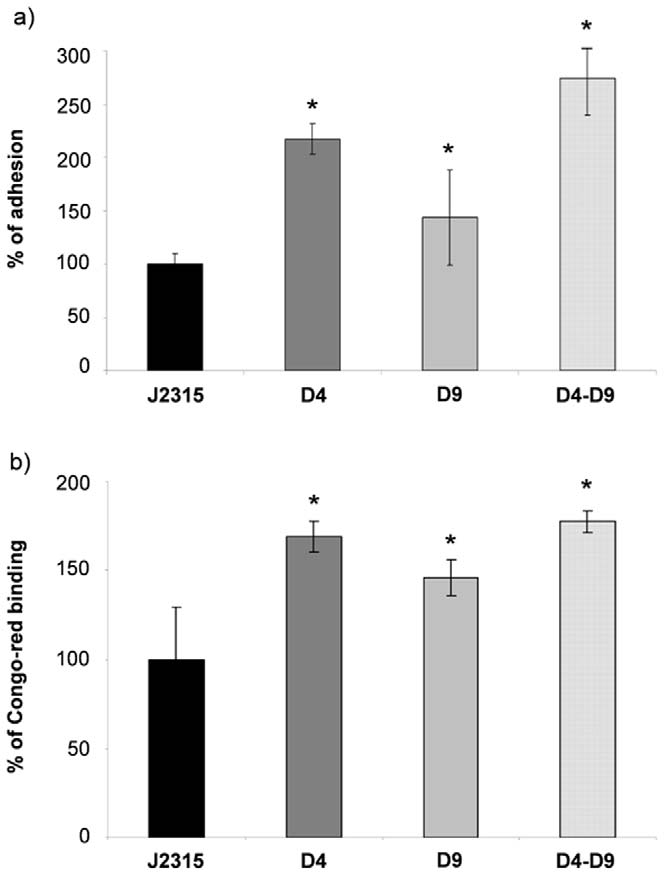
Effect of RND-4 and RND-9 mutations on biofilm formation. (A) Adhesion to polyvinyl chloride mitrotiter plates measured by crystal violet staining. (B) Congo red dye binding ability. In both cases, results are given as a percentage, considering *B. cenocepacia* J2315 (wild-type) as 100%. The mean of three different experiments with standard deviation is reported. Significantly differences with respect to J2315 are indicated by an * (p<0.01). J2315, *B. cenocepacia* wild-type; D4, RND-4 mutant; D9, RND-9 mutant; D4–D9, RND4-RND9 mutant.

It is known that in many bacteria flagella could play a role also in adhesion and biofilm formation [42 and references therein]. Therefore, we performed a preliminary investigation about the ability of the four strains to produce biofilm by using two standard methods: adhesion to polyvinyl chloride microplates and Congo red binding. The two methods gave comparable results and, surprisingly, demonstrated that all the mutants showed enhanced biofilm formation, with respect to the wild-type (Figure 3).

### Phenotype MicroArray analysis

**Figure 4:**
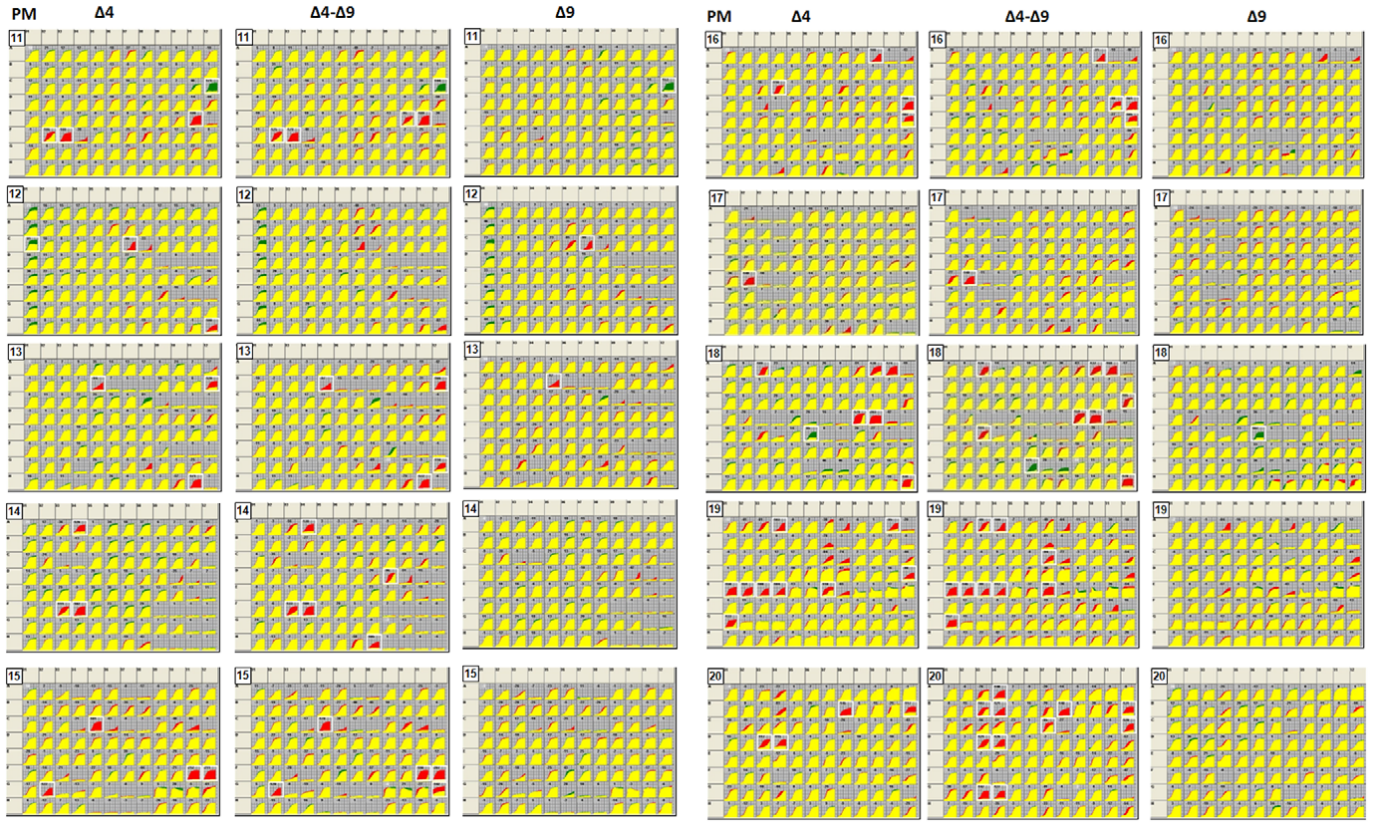
The Phenotype Microarray profile of *B.* cenocepacia J2315 and the RND mutants. Metabolic plates (from PM 11 to PM20) representing the growth of the three *B. cenocepacia* mutant strains D4, D9 and D4–D9 *versus* the wild-type strain J2315, in the presence of toxic compounds is shown.

To check the effect of the deletion of RND-4 and/or RND-9 operons on the phenotype of the strain *B. cenocepacia* J2315, a Phenotype MicroArray (PM) (Biolog) analysis was performed. Phenotype MicroArray [29], [43] is a technology allowing to quantitatively measure thousands of cellular phenotypes all at once. Ten different panels (PM11-PM20) that enable chemical sensitivity tests for bacteria, were analyzed. A total of 240 chemicals at four different concentrations were tested out (a more detailed information about the PM panels is available at http://www.biolog.com). Data obtained are shown in Figure 4.

**Figure 5:**
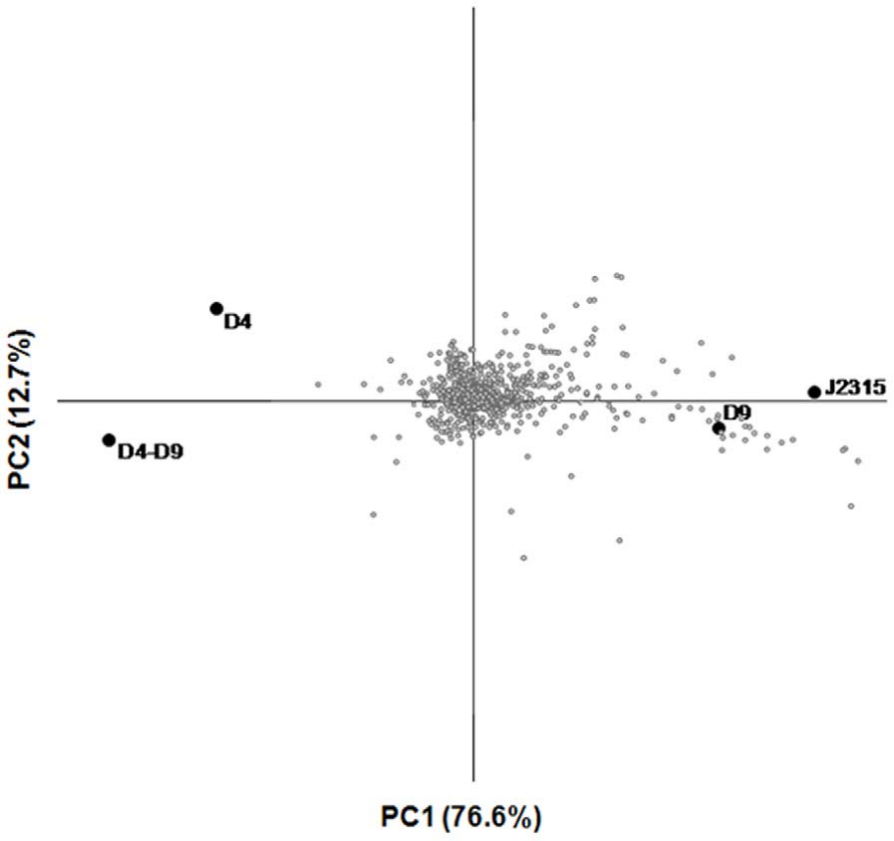
Principal component analysis of phenotype microarrays profiles of *B. cenocepacia* J2315 and D4, D9, D4–D9 mutants, obtained from an analysis of 960 chemical sensitivity tests (PM11-PM20). The figure shows the four strains (J2315, D4, D9, D4–D9) and the phenotypical tests plotted in an X-Y diagram corresponding to the first two components.

Principal component analysis (PCA) was applied to the PM data to study the differences between the phenotype profiles of the four strains in more detail (Figure 5). PCA separated the isolates into two groups by the first component (which accounted for 76.6% of phenotypic variation). One group housed wild-type and mutant D9, and the other included mutants D4 and D4–D9. The second component (which accounted for 12.7% of phenotypic variation) provided a fairly good separation of strains D4 and D4–D9, and did not allow the separation of wild-type and D9 mutant. These results suggested that D9 mutant has a phenotype very similar to that of the wild-type strain, while D4 is phenotypically different from the wild-type and similar to the D4–D9 mutant. The compounds under which the differences between the area of the kinetic curves of the wild-type and mutant strains were over 15000 Biolog units in at least one of the concentrations for each chemical assayed were selected and IC50 values are shown in Table S8. In agreement with the inactivation procedure used in this work, which makes use of a tetracycline resistance cassette, the three mutants exhibited a decreased sensitivity to minocycline, an antibiotic belonging to the tetracycline family. The mutants D4 and D4–D9 showed an increased sensitivity in respect to the wild-type to different types of compounds: antibiotics, DNA intercalators, drugs, fungicides, detergents, toxic anions, ionophores, uncouplers, oxidizing agents.

## Discussion

In order to investigate the contribution of efflux pumps to intrinsic drug resistance of *B. cenocepacia* J2315, we recently deleted 3 operons encoding the putative RND transporters RND-1 (BCAS0591*-*BCAS0593), RND-3 (BCAL1674*-*BCAL1676), and RND-4 (BCAL2822*-*BCAL2820) [18]. We named the corresponding inactivated strains D1, D3, and D4. The *B. cenocepacia* D3 and D4 mutants demonstrated increased sensitivity to inhibitory compounds, suggesting an involvement of these proteins in the intrinsic drug resistance of *B. cenocepacia* J2315. In contrast, deletion of the RND-1 operon did not lead to detectable phenotype alterations under the conditions assayed [18]. In this work we continued in the same direction and analyzed the effect of the deletion of operon encoding RND-9 efflux pump (BCAM1945–1947 genes) in both the wild-type strain (thus obtaining D9 mutant), and in the D4 strain (thus obtaining the double D4–D9 mutant). Understanding the role of RND efflux transporters in *B. cenocepacia* is fundamental to highlight their involvement in drug resistance. Here, by integrating transcriptomics, phenomics, and a set of different phenotypic assays, we have expanded our previous work [18] and, in general, our knowledge on the role of this clinically important protein family. In particular, we have focused our attention on RND-4 and RND-9 efflux pump encoding operons, characterizing the deleted mutants D4, D9 and the double mutant D4–D9 by a combination of different experimental approaches. We used the Phenotype MicroArray (phenomic) procedure, a new technology that allows to quantitatively measure thousands of cellular phenotypes all at once, to check the ability of the wild-type and mutant strains to pump out different toxic metabolites. This phenomic analysis confirmed and strengthened previous data obtained by Buroni *et al*. [18] on mutant D4, showing that RND-4 is involved in the extrusion of a wide variety of compounds toxic for cell metabolism, in agreement with antimicrobial susceptibilities of the mutant as previously determined [18]. Similar results were obtained for the double mutant D4–D9.

Concerning mutant D9, the scenario is more intriguing; indeed, RND-9 seems to be only partially involved in drug efflux, showing MIC values only 2-fold lower than the wild-type strain for a few drugs, at least in our experimental conditions. These data are in full agreement with Phenotype Microarray analysis, which revealed that D9 mutant had a phenotype very similar to the wild-type strain. This opens the intriguing question of the role that this operon may play *in vivo*. However, since *B. cenocepacia* J2315 shows many genes involved in antibiotic resistance, many of which might have (partially) overlapping functions, it is quite possible that some of them might act in a synergistic fashion in determining the intrinsic resistance to one or more toxic compounds. So a two-fold decrease in MIC in the D9 deletion mutant is a proof that this pump may be involved in resistance to these antibiotics. Besides, as shown by Perrin *et al.* [17], BCAM1946 protein sequence (which appertains to RND-9 operon) belongs to the same phylogenetic cluster embedding BCAL2821 (which is part of RND-4), but to a different and distant branch, very close to the widely distributed RND-10 (BCAM2549-51); lastly, the phylogenetic distribution of RND-9 is very narrow, in that its orthologs were shown to be present only in a few Bcc species [17]. This might suggest that the absence of RND-9 function in D9 mutant could be replaced by other efflux systems, belonging to the same and/or to different phylogenetic clusters. An alternative, even though not mutually exclusive possibility, is that since the toxic compounds tested are not metabolic intermediates produced by *Burkholderia* cells, RND-9 is involved in the efflux of toxic (or even not-toxic) molecules produced by the microorganism under different physiological conditions.

The phenotypic similarity shared by mutants D4 and D4–D9 was confirmed also at the molecular level by the transcriptome analysis. Indeed, the microarray results showed that D4 and D4–D9 mutants have a similar expression profile, in particular motility and chemotaxis-related genes appear to be up-regulated in both strains. In contrast, the same genes are down-regulated or not differentially expressed in D9 mutant. Most differentially regulated genes of the single mutants were also differentially regulated in the double mutant, and for the most part in the same directionality. This illustrated how the double mutant displays a combined, additive expression profile of both single mutants and one would therefore expect to see an additive phenotype. The overall trend of gene expression was confirmed by qRT-PCR experiments by Pearson correlation, indicating that the microarray for *B. cenocepacia* is reliable to assess gene expression changes in this strain as has been shown in previous studies [21], [44]. Moreover, data are consistent with the observations from the motility assays, in which the D4 and the double mutant show enhanced swimming motility with respect to the wild-type, in contrast with mutant D9 where this phenomenon is reduced. Moreover, D4 has 12 more up-regulated genes involved in motility than D4–D9, as reported in Table 4. This could be an explanation to the fact that this mutant is more motile than the double mutant D4–D9 (Fig. 2). In this view, it seems that D9 mutation is able to partially suppress the effects of the D4 mutation, at least for what concerns swimming.

Regarding chemotaxis, despite the differences observed in the microarray analysis, the three mutants showed the same chemotactic phenotype at least under our experimental conditions. It is possible that differences in chemotaxis might be appreciated by the use of specific attractant or repellent molecules. However, it is not trivial to identify such specific compounds and further studies should be performed in order to address this point.

These unexpected and interesting results strongly suggest that the biological role of the RND-4 and RND-9 efflux pumps might not be restricted to the sole transport of toxic (and/or not toxic) compounds, but also that their function might be related to motility and/or chemotaxis. To the best of our knowledge, this is the second time that the effect of RND efflux pumps mutation on motility-related phenotypes has been described. Indeed, the absence of RND components AcrB or TolC in *Salmonella enterica* caused widespread repression of chemotaxis and motility genes in these mutants, and for *acrB* mutant this was associated with decreased motility [45]. However, why the deletion of an efflux pump should have a fallout on bacterial motility and chemotaxis remains an open question. It is conceivable that the cytoplasmic accumulation of efflux pump-specific metabolites (different for each mutant) could act as signals triggering opposite behavioural response in the two mutants. For instance, we have recently shown that RND-4 contributes to the transport of *N*-acyl homoserine lactone (AHLs) as we found a reduced accumulation of AHLs quorum sensing (QS) signal molecules in the growth medium of D4 mutant [18]. Actually, the D4 and D4/D9 mutant produce about 30% less AHLs than the wild-type, while D9 produces almost the same level of acyl-HSL as the wild-type ([18] and Figure S7). In accordance with the low impact of D4 and D9 mutations on AHLs production, only few genes known to be AHL-regulated are also differentially regulated in our microarray analysis (Table S9). Among these, none can be directly related to chemotaxis or biofilm formation, and only BCAL0562 and BCAL3506 could be related to flagella. Overall, these observations suggest that it is unlikely that the phenotype of the D4, D9 and D4–D9 mutants is due to an unbalance in AHLs import/export rates. However, it cannot be ruled out that other molecules acting as metabolic signals could accumulate in the D4, D9 and D4–D9 mutants and account for the motility and biofilm phenotypes of these strains. Another possible explanation for the biological significance of the phenotype exhibited by D4 and D4–D9 strains might rely on the assumption that: i) the bacterial cell can “sense” the concentration of toxic compounds outside and/or inside the cell and that ii) the cell itself tries to respond to the increase of the concentration of toxic compound(s) by activating the efflux pump systems responsible for the extrusion of that compound(s). Accordingly, we can speculate that in the absence (such as in D4 and D4–D9 mutants) of these systems, the cell might somehow bypass this defect by increasing the ability to move in the environment in order to “escape” and to explore spaces and niches where the concentration of the toxic compounds is lower. In other words, the increased ability to move might represent a sort of “indirect protection” of the cell towards toxic compounds.

Since in many bacteria flagellum could play a role in biofilm formation, the different regulation of flagellum-related genes in D4 and D9 prompted us to speculate that these strains might also have opposite biofilm phenotypes. Therefore, we performed preliminary experiments to investigate the biofilm formation ability of the wild-type and of the three mutants. Results showed, surprisingly, that all the mutants had an enhancement of biofilm formation with respect to the wild-type. Therefore, differences in flagella expression in the D4 and D9 strains, with respect to the wild-type, play a minor role in biofilm formation, at least under our experimental conditions. The increased biofilm production of the RND-mutants was unexpected since we did not identify genes obviously involved in biofilm formation among the 33 having the same expression pattern in the three microarray experiments (Figure 1 and Table S1). Actually, biofilm formation is a complex pleiotropic phenotype, strongly dependent upon experimental conditions and growth media [46], [47]. Therefore, it is not easy to correlate the microarray data derived from planktonic cultures with the increased biofilm production of the RND-mutants, with respect to the wild-type. However, 19 out of the 24 genes up-regulated in all the microarray experiments, are phage-related genes (located in the region spanning from ORFs BCAS0506 to BCAS0554; Table S1, Figure 1). Over-expression of phage-related genes in sessile cells compared with planktonic cells and/or increased expression in response to stress has been observed in several species [47 and references therein]. Bacterial stress response can increase the mobility of bacteriophages, and it has been proposed that prophage production may play a role in generating genetic diversity in the biofilm [47 and references therein]. It is tempting to speculate that cytoplasmic accumulation of toxic metabolites and/or metabolic signals due to the lack of RND-4 and/or RND-9 efflux pumps could produce a general stress response triggering the expression of genes involved in biofilm formation. This finding stimulates future studies on the role played by RND pumps in the efflux of endogenously produced molecules potentially involved in virulence and host colonization (*e.g.* biofilm matrix components, biologically active secondary metabolites, signal molecules), besides their role in drug resistance. The biofilm experiment also showed that D9 produces less biofilm than D4 and D4–D9. This result might be explained, at least in part, by the observation that, besides flagella genes, also cellulose biosynthetic genes (ORFs BCAL1391 and BCAL1395, Table S1) were up- and down-regulated in the D4 and D9 mutants, respectively, and the D9 showed down-regulation of fimbrial genes (ORFs BCAL0959 and BCAL2636, Table S1).

The different expression of genes involved in pathways strongly related to virulence is a first step towards a better understanding of *B. cenocepacia* pathogenesis. A relevant point is that inactivation of efflux pumps enhances biofilm formation and, sometimes, motility. If this is true also in the host, the use of efflux pump inhibitors could be, on one side positive for helping the antibiotic therapy, on the other side, it could promote biofilm formation and chronic infection. More detailed study on the effect of RND efflux pumps in virulence-related phenotype and chronic infection are strongly desirable.

In the future the construction of a multiple inactivated strain will be helpful both to understand if the lack of these proteins may affect pathways important for the life of the pathogen and, hopefully, to construct an attenuated strain, for the design of a suitable vaccine.

## Supporting Information

Figure S1 **Pie chart representing Gene Ontology (GO) terms distribution in** ***B. cenocepacia*** **D4 mutant up-regulated genes.** Representation of the functional classes at the different nodes of one level in GO term association analysis.
(TIF)

Figure S2 **Pie chart representing Gene Ontology (GO) terms distribution in** ***B. cenocepacia*** **D4 mutant down-regulated genes.** Representation of functional classes at the different nodes of one level in GO term association analysis.
(TIF)

Figure S3 **Pie chart representing Gene Ontology (GO) terms distribution in** ***B. cenocepacia*** **D9 mutant up-regulated genes.** Representation of functional classes at the different nodes of one level in GO term association analysis.
(TIF)

Figure S4 **Pie chart representing Gene Ontology (GO) terms distribution in** ***B. cenocepacia*** **D9 mutant down-regulated genes.** Representation of functional classes at the different nodes of one level in GO term association analysis.
(TIF)

Figure S5 **Pie chart representing Gene Ontology (GO) terms distribution in** ***B. cenocepacia*** **D4–D9 mutant up-regulated genes.** Representation of functional classes at the different nodes of one level in GO term association analysis.
(TIF)

Figure S6 **Pie chart representing Gene Ontology (GO) terms distribution in** ***B. cenocepacia*** **D4–D9 mutant down-regulated genes.** Representation of functional classes at the different nodes of one level in GO term association analysis.
(TIF)

Figure S7 **Evaluation of AHLs accumulation in the growth medium of** ***B. cenocepacia*** **J2315 and RND mutants.** AHL measurement was carried out using *E. coli* (pSCR1) as described by Buroni *et al*. [18]. AHL was extracted from spent supernatants, AHL levels were measured with a volume of extract corresponding to 10^9^ CFU. Values of AHL accumulated in the supernatant are in percentage in relation to the wild-type strain. The experiments were performed in triplicate giving comparable results. Significantly differences with respect to J2315 are indicated by an * (p<0.05). J2315, *B. cenocepacia* wild-type; D4, RND-4 mutant; D9, RND-9 mutant; D4–D9, RND4-RND9 mutant.
(TIFF)

Table S1 **Complete list of genes up- or down-regulated in** ***B. cenocepacia*** **strains D4, D9, D4–D9** ***versus*** **J2315 deriving from the microarray analysis.**
(DOC)

Table S2 **Gene Ontology (GO) terms functional enrichment analysis showing the over or under-representation of up-regulated genes of mutant D4 in comparison to** ***B. cenocepacia*** **J2315 whole genome functional annotation.** Only GO terms over- or under- represented with an associated p-value <0.05 are shown.
(DOC)

Table S3 **Gene Ontology (GO) terms functional enrichment analysis showing the over or under-representation of down-regulated genes of mutant D4 in comparison to** ***B. cenocepacia*** **J2315 whole genome functional annotation.**
(DOC)

Table S4 **Gene Ontology (GO) terms functional enrichment analysis showing the over or under-representation of up-regulated genes of mutant D9 in comparison to** ***B. cenocepacia*** **J2315 whole genome functional annotation.** Only GO terms over- or under- represented with an associated p-value <0.05 are shown.
(DOC)

Table S5 **Gene Ontology (GO) terms functional enrichment analysis showing the over or under-representation of down-regulated genes of mutant D9 in comparison to** ***B. cenocepacia*** **J2315 whole genome functional annotation.** Only GO terms over- or under- represented with an associated p-value <0.05 are shown.
(DOC)

Table S6 **Gene Ontology (GO) terms functional enrichment analysis showing the over or under-representation of up-regulated genes of mutant D4-D9 in comparison to** ***B. cenocepacia*** **J2315 whole genome functional annotation.** Only GO terms over- or under- represented with an associated p-value <0.05 are shown.
(DOC)

Table S7 **Gene Ontology (GO) terms functional enrichment analysis showing the over or under-representation of down-regulated genes of mutant D4–D9 in comparison to** ***B. cenocepacia*** **J2315 whole genome functional annotation.** Only GO terms over- or under- represented with an associated p-value <0.05 are shown.
(DOC)

Table S8 **Schematic representation of data obtained from PM (from PM11 to PM20) analyses of** ***B. cenocepacia*** **strain J2315, D4, D9 and D4–D9.** *IC50 was calculated on the basis of the kinetic curves obtained on the four different concentrations of each chemical compound and it was defined as the well or fraction of a well at which the area of kinetic curve is at half of its maximal value over the concentration series. **IC50 is reported only for compounds under which the difference between the areas of the kinetic curves of wild-type and mutant strain was over 15000 units in at least one of the concentrations tested.
(DOC)

Table S9 **List of genes differentially regulated in** ***B. cenocepacia*** **strains D4, D9, D4–D9** ***versus*** **J2315 known to be also controlled by AHL-based quorum sensing.**
(DOC)
