# Supplementary figures and images for: Deciphering the Role of RND Efflux Transporters in Burkholderia cenocepacia
Source: PLoS One. 2011 Apr 19;6(4):e18902. doi: 10.1371/journal.pone.0018902 (PMC3079749; doi:10.1371/journal.pone.0018902)

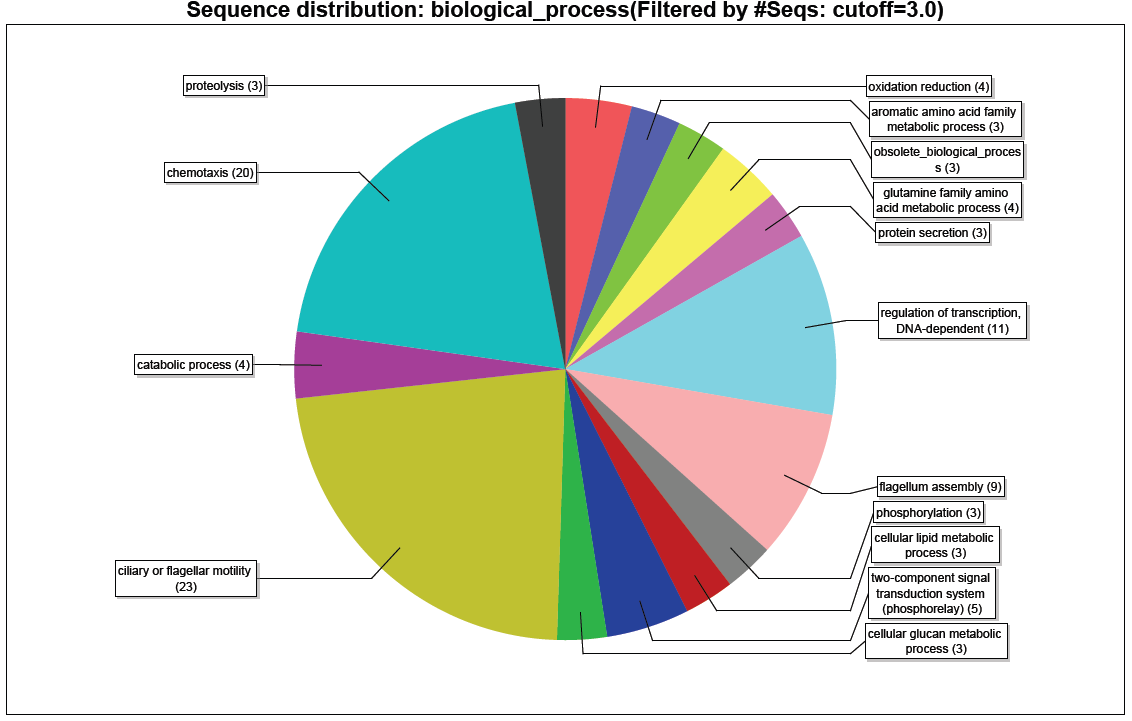

Supplement: Figure S1 — Pie chart representing Gene Ontology (GO) terms distribution in B. cenocepacia D4 mutant up-regulated genes. Representation of the functional classes at the different nodes of one level in GO term association analysis. (TIF) [file pone.0018902.s001.tif]

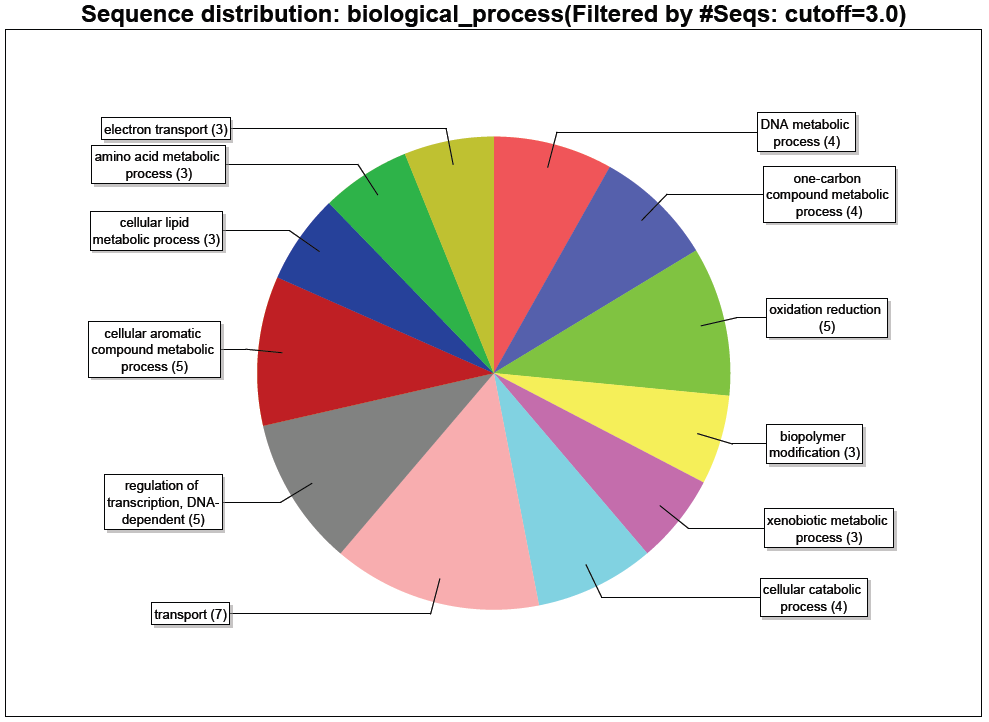

Supplement: Figure S2 — Pie chart representing Gene Ontology (GO) terms distribution in B. cenocepacia D4 mutant down-regulated genes. Representation of functional classes at the different nodes of one level in GO term association analysis. (TIF) [file pone.0018902.s002.tif]

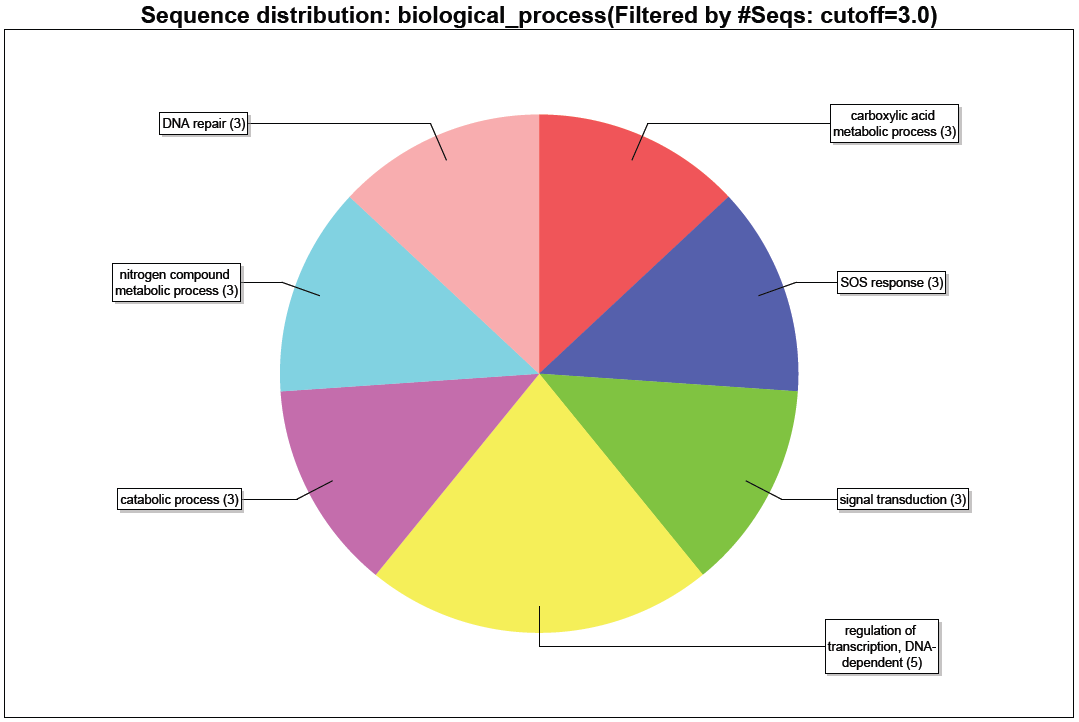

Supplement: Figure S3 — Pie chart representing Gene Ontology (GO) terms distribution in B. cenocepacia D9 mutant up-regulated genes. Representation of functional classes at the different nodes of one level in GO term association analysis. (TIF) [file pone.0018902.s003.tif]

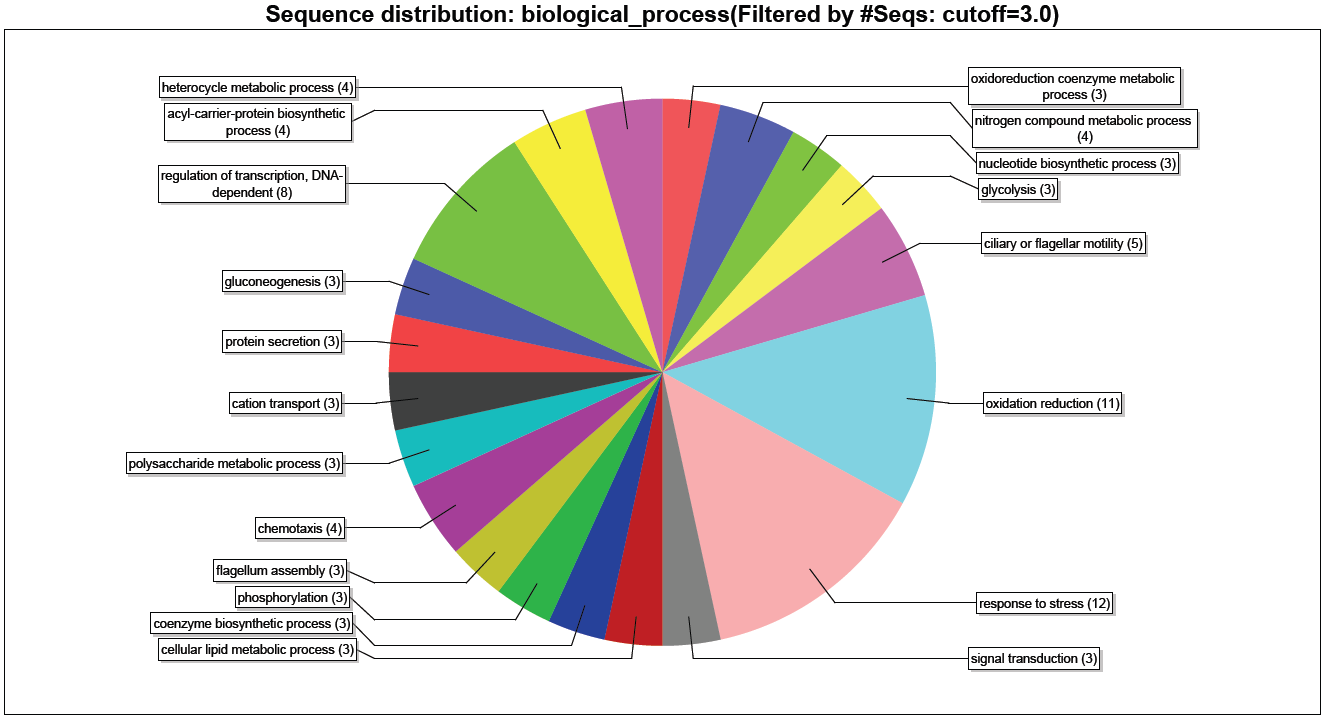

Supplement: Figure S4 — Pie chart representing Gene Ontology (GO) terms distribution in B. cenocepacia D9 mutant down-regulated genes. Representation of functional classes at the different nodes of one level in GO term association analysis. (TIF) [file pone.0018902.s004.tif]

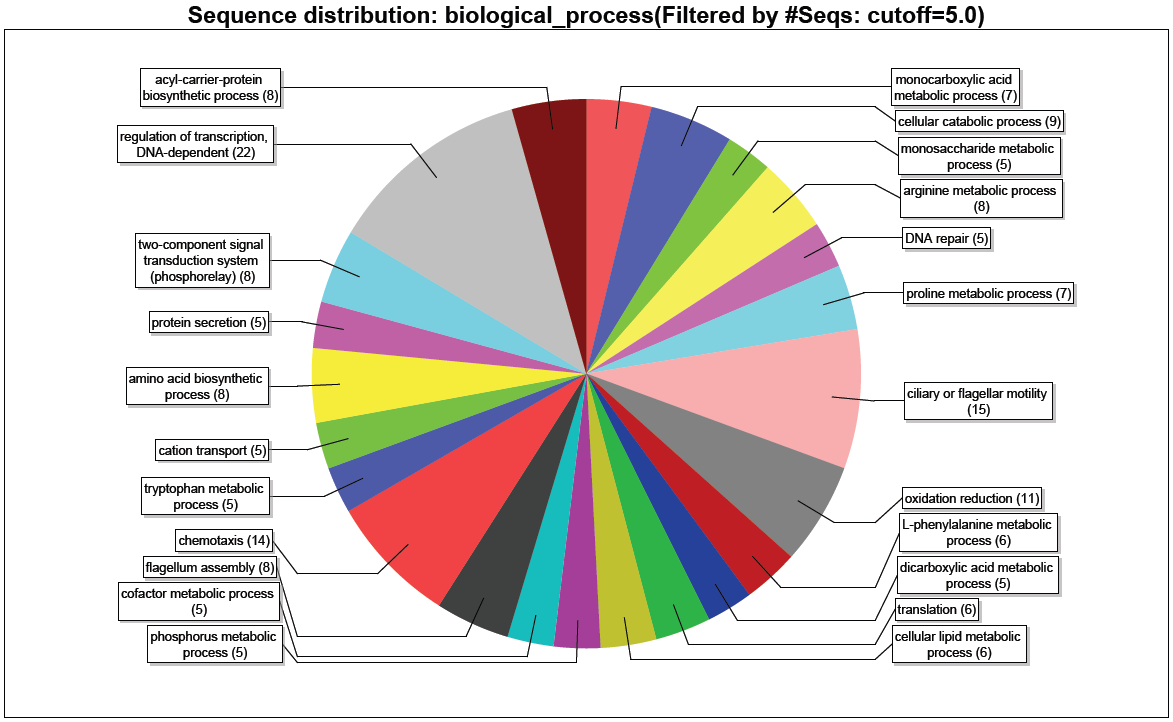

Supplement: Figure S5 — Pie chart representing Gene Ontology (GO) terms distribution in B. cenocepacia D4–D9 mutant up-regulated genes. Representation of functional classes at the different nodes of one level in GO term association analysis. (TIF) [file pone.0018902.s005.tif]

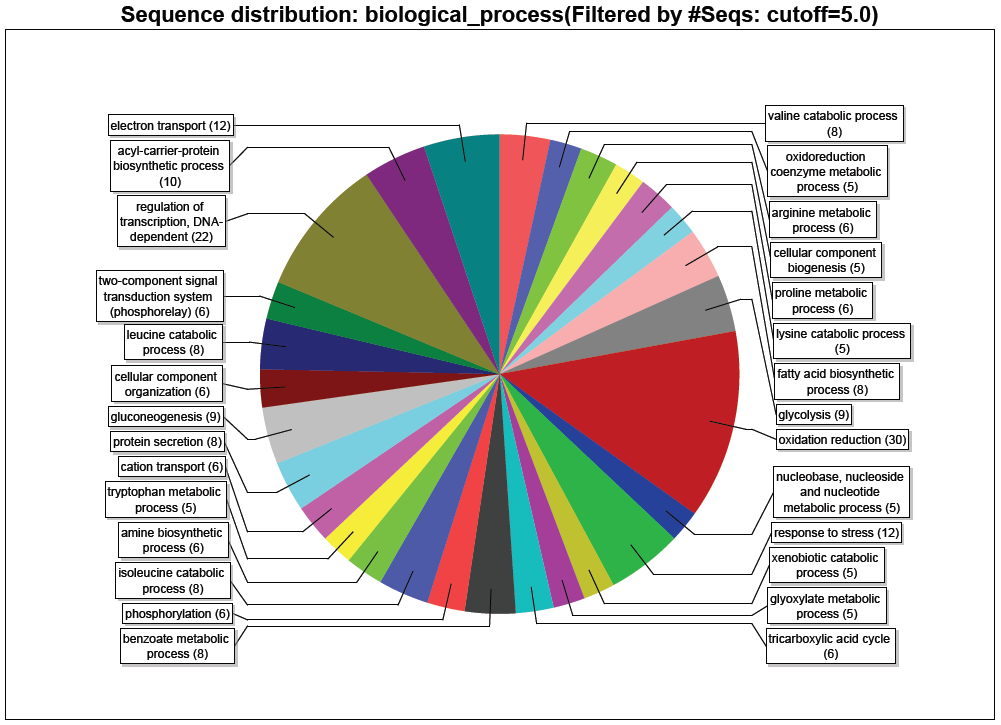

Supplement: Figure S6 — Pie chart representing Gene Ontology (GO) terms distribution in B. cenocepacia D4–D9 mutant down-regulated genes. Representation of functional classes at the different nodes of one level in GO term association analysis. (TIF) [file pone.0018902.s006.tif]

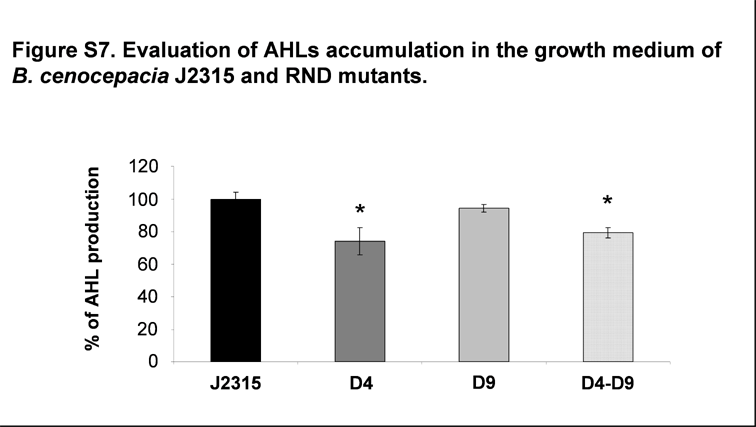

Supplement: Figure S7 — Evaluation of AHLs accumulation in the growth medium of B. cenocepacia J2315 and RND mutants. AHL measurement was carried out using E. coli (pSCR1) as described by Buroni et al. [18]. AHL was extracted from spent supernatants, AHL levels were measured with a volume of extract corresponding to 109 CFU. Values of AHL accumulated in the supernatant are in percentage in relation to the wild-type strain. The experiments were performed in triplicate giving comparable results. Significantly differences with respect to J2315 are indicated by an * (p<0.05). J2315, B. cenocepacia wild-type; D4, RND-4 mutant; D9, RND-9 mutant; D4–D9, RND4-RND9 mutant. (TIFF) [file pone.0018902.s007.tif]
