# Supplementary material for: Deciphering the Role of RND Efflux Transporters in Burkholderia cenocepacia
Source: PLoS One. 2011 Apr 19;6(4):e18902. doi: 10.1371/journal.pone.0018902 (PMC3079749; doi:10.1371/journal.pone.0018902)
Supplement: Table S1 — Complete list of genes up- or down-regulated in B. cenocepacia strains D4, D9, D4–D9 versus J2315 deriving from the microarray analysis. (DOC) [file pone.0018902.s008.doc]

**Table S1.** **Complete list of genes up- or down-regulated in *B. cenocepacia* strains D4, D9, D4-D9 *versus* J2315 deriving from the microarray analysis.**

| **Gene no.** | **Description** | **Change in gene expression (log_2_ fold change)** | | |
| --- | --- | --- | --- | --- |
|  |  | **D4 vs J2315** | **D9 vs J2315** | **D4-D9 vs J2315** |
| BCAL0010 | phenylalanine-4-hydroxylase | - | - | 2.02 |
| BCAL0012 | putative adenylate cyclase | - | -0.59 | - |
| BCAL0042 | bifunctional PutA protein | - | - | 0.82 |
| BCAL0043 | putative extracellular ligand-binding protein | - | - | 0.70 |
| BCAL0052 | putative oxidoreductase | - | - | 0.60 |
| BCAL0057 | putative membrane protein | -1.09 | - | - |
| BCAL0105 | putative phage membrane protein | - | - | -0.76 |
| BCAL0108 | putative membrane protein | -3.66 | - | -4.58 |
| BCAL0110 | putative aminotransferase | 3.28 | - | - |
| BCAL0111 | putative TPR repeat protein | 2.64 | - | 1.05 |
| BCAL0112 | conserved hypothetical protein | 4.32 | -0.68 | 2.99 |
| BCAL0113 | flagellar hook-associated protein | 4.90 | - | 3.75 |
| BCAL0114 | flagellin (type II) | 7.76 | - | 4.97 |
| BCAL0121 | aquaporin Z | - | - | -1.12 |
| BCAL0124 | flagellar regulon master regulator subunit FlhD | 3.59 | - | 1.52 |
| BCAL0125 | flagellar regulon master regulator subunit FlhC | 3.31 | -1.16 | 2.03 |
| BCAL0126 | chemotaxis protein MotA | 3.43 | - | 2.27 |
| BCAL0127 | chemotaxis protein MotB | 3.09 | - | 2.02 |
| BCAL0128 | chemotaxis two-component response regulator CheY | 3.32 | - | 2.44 |
| BCAL0129 | chemotaxis two-component sensor kinase CheA | 3.52 | -1.45 | 1.91 |
| BCAL0130 | chemotaxis protein CheW | 2.99 | - | - |
| BCAL0131 | methyl-accepting chemotaxis protein | 1.48 | - | - |
| BCAL0132 | chemotaxis protein methyltransferase CheR | 3.49 | - | 1.49 |
| BCAL0133 | putative chemoreceptor glutamine deamidase CheD | 3.33 | -1.36 | 1.86 |
| BCAL0134 | chemotaxis protein-glutamate methylesterase CheB1 | 3.19 | -0.83 | 1.73 |
| BCAL0135 | chemotaxis protein CheY2 | 2.55 | - | 1.34 |
| BCAL0136 | chemotaxis protein CheZ | 2.48 | -0.62 | 1.46 |
| BCAL0137 | conserved hypothetical protein | 1.85 | - | 0.61 |
| BCAL0140 | flagellar biosynthetic protein FlhB | 3.78 | -1.88 | 2.63 |
| BCAL0142 | flagellar biosynthesis protein FlhF | 3.25 | -0.82 | 2.28 |
| BCAL0143 | flagellar biosynthesis protein FlhG | 4.53 | - | 1.85 |
| BCAL0144 | RNA polymerase sigma factor for flagellar regulon FliA | 2.52 | - | 1.05 |
| BCAL0149 | putative dienelactone hydrolase | - | - | 0.70 |
| BCAL0160 | putative methylase | - | - | 0.62 |
| BCAL0165 | putative plasmid replication-associated protein | -2.73 | - | -2.54 |
| BCAL0168 | hypothetical protein | -5.83 | - | -5.30 |
| BCAL0169 | conserved hypothetical protein | -6.08 | - | -5.00 |
| BCAL0170 | conserved hypothetical protein | -1.93 | - | - |
| BCAL0171 | putative plasmid conjugal transfer protein | -1.91 | - | -1.89 |
| BCAL0172 | putative plasmid conjugal transfer protein | -1.93 | - | -1.05 |
| BCAL0173 | putative plasmid conjugal transfer protein | - | - | -0.85 |
| BCAL0174 | putative plasmid conjugal transfer protein | -1.19 | - | - |
| BCAL0175 | conserved hypothetical protein | -3.62 | - | -2.29 |
| BCAL0176 | conserved hypothetical protein | -2.78 | - | -0.93 |
| BCAL0177 | putative plasmid conjugal transfer protein | -4.76 | - | -2.87 |
| BCAL0178 | putative DNA methyltransferase | -3.11 | - | - |
| BCAL0179 | hypothetical protein | -8.34 | - | -5.01 |
| BCAL0180 | putative membrane protein | -7.17 | - | -3.15 |
| BCAL0181 | conserved hypothetical protein | -6.28 | - | -5.10 |
| BCAL0182 | putative plasmid recombinase | -4.02 | - | -2.98 |
| BCAL0198 | putative outer membrane protein | - | - | -1.06 |
| BCAL0199 | putative lipoprotein | -1.00 | - | - |
| BCAL0200 | putative lipoprotein | - | - | -0.75 |
| BCAL0206A | putative outer membrane protein | - | - | -0.66 |
| BCAL0229 | 30S ribosomal protein S12 | - | - | 0.84 |
| BCAL0230 | 30S ribosomal protein S7 | - | - | 0.78 |
| BCAL0258 | 30S ribosomal protein S11 | - | - | 0.74 |
| BCAL0261 | 50S ribosomal protein L17 | - | - | 0.63 |
| BCAL0268 | putative cytochrome c biogenesis protein | - | - | -0.64 |
| BCAL0278 | putative type IV pilus secretion protein | - | - | -1.02 |
| BCAL0289 | glutamate synthase large subunit | 1.21 | - | - |
| BCAL0290 | glutamate synthase small subunit | 1.46 | - | 0.61 |
| BCAL0291 | sodium:amino acid symporter family protein | - | - | 0.63 |
| BCAL0311 | ATP phosphoribosyltransferase | - | - | -1.44 |
| BCAL0355 | putative transmembrane protein (fragment) | - | - | -1.51 |
| BCAL0366 | nitroreductase family protein | - | - | 0.65 |
| BCAL0388 | putative monooxygenase | - | -0.78 | -0.64 |
| BCAL0433 | spermidine N(1)-acetyltransferase | 1.41 | - | 0.96 |
| BCAL0438 | putative DNA-3-methyladenine glycosylase II | - | - | -0.88 |
| BCAL0446 | putative aminotransferase | - | - | 0.70 |
| BCAL0448 | ABC transporter. ATP-binding protein | - | - | -1.00 |
| BCAL0511 | putative deoxygenase | - | -1.10 | -0.99 |
| BCAL0514 | putative membrane protein | - | -0.91 | -1.34 |
| BCAL0518 | putative coniferyl aldehyde dehydrogenase | - | - | -1.15 |
| BCAL0520 | putative flagellar hook-length control protein FliK | 2.98 | -1.21 | 2.31 |
| BCAL0521 | flagellar FliJ protein | 3.23 | - | 1.88 |
| BCAL0522 | flagellum-specific ATP synthase FliI | 3.55 | -1.85 | 2.35 |
| BCAL0523 | flagellar assembly protein FliH | 3.73 | - | 2.16 |
| BCAL0524 | flagellar motor switch protein FliG | 2.03 | - | - |
| BCAL0525 | flagellar M-ring protein FliF | 2.16 | - | - |
| BCAL0526 | fliE, flagellar hook-basal body complex protein FliE | 2.19 | - | - |
| BCAL0527 | flagellar protein FliS | 3.25 | - | 2.89 |
| BCAL0528 | conserved hypothetical protein | 2.71 | - | 2.07 |
| BCAL0529 | conserved hypothetical protein | - | - | -3.04 |
| BCAL0534 | two-component regulatory system, response regulator | - | - | -0.80 |
| BCAL0541 | putative FAD dependent oxidoreductase | - | -0.68 | - |
| BCAL0544 | putative periplasmic dipeptide transport protein | - | - | 0.82 |
| BCAL0549 | putative exported protein | - | - | -3.65 |
| BCAL0561 | putative flagella synthesis protein FlgN | 2.23 | - | 1.38 |
| BCAL0562 | putative negative regulator of flagellin synthesis (anti-sigma-28 factor) | 2.81 | - | 1.34 |
| BCAL0564 | putative flagellar basal-body rod protein FlgB | 3.44 | - | - |
| BCAL0565 | flagellar basal-body rod protein FlgC | 3.23 | - | 1.91 |
| BCAL0566 | putative basal-body rod modification protein FlgD | 4.88 | -1.21 | 2.56 |
| BCAL0567 | putative flagellar hook protein FlgE1 | 4.19 | -1.31 | 2.36 |
| BCAL0568 | flagellar basal-body rod protein FlgF | 4.02 | -1.34 | 2.43 |
| BCAL0569 | flagellar basal-body rod protein FlgG | 4.10 | - | 2.35 |
| BCAL0570 | flagellar L-ring protein precursor | 3.14 | -1.21 | 1.94 |
| BCAL0571 | flagellar P-ring protein precuror | 2.85 | -0.61 | 1.83 |
| BCAL0572 | putative peptidoglycan hydrolase FlgJ | 2.37 | - | 1.27 |
| BCAL0575 | YcgR family protein | 1.69 | - | 1.00 |
| BCAL0576 | putative flagellar hook-associated protein (HAP1) | 4.41 | - | - |
| BCAL0577 | putative flagellar hook-associated protein (HAP3) | 4.31 | - | 3.99 |
| BCAL0582 | putative membrane protein | -1.15 | - | -1.11 |
| BCAL0600 | putative glutamine synthetase | - | - | -0.91 |
| BCAL0620 | LacI family regulatory protein | - | - | -0.95 |
| BCAL0625 | LysR family regulatory protein | - | - | -0.83 |
| BCAL0627 | putative hydrolase | -1.01 | - | - |
| BCAL0659 | allophanate hydrolase subunit 1 | -1.60 | - | -2.02 |
| BCAL0693 | Bordetella pertussis Bvg accessory factor family protein | - | -0.65 | - |
| BCAL0695 | putative membrane protein | - | - | -1.54 |
| BCAL0703 | serine peptidase | - | - | -1.28 |
| BCAL0762 | putative methyl-accepting chemotaxis protein | 1.96 | - | 1.58 |
| BCAL0768 | conserved hypothetical protein | - | 0.95 | 1.00 |
| BCAL0770 | putative DNA polymerase III alpha subunit | - | 0.80 | 0.86 |
| BCAL0779 | putative phosphosugar-binding protein | - | - | -2.70 |
| BCAL0783 | putative membrane protein | - | - | -1.92 |
| BCAL0785 | cytochrome d ubiquinol oxidase subunit I | - | - | -1.41 |
| BCAL0786 | putative membrane protein | - | - | -1.14 |
| BCAL0792 | putative maleylacetoacetate isomerase | -1.10 | - | - |
| BCAL0812 | sigma 54 modulation protein | -1.42 | -1.50 | -1.15 |
| BCAL0833 | putative Acetoacetyl-CoA reductase phbB | - | -0.94 | - |
| BCAL0854 | GntR family regulatory protein | - | - | 1.02 |
| BCAL0889 | conserved hypothetical protein | - | -0.94 | - |
| BCAL0925 | putative glycerol kinase | - | - | 1.08 |
| BCAL0940 | putative transglycosylase | - | - | -0.62 |
| BCAL0950 | Major Facilitator Superfamily protein | - | -0.71 | -0.67 |
| BCAL0953 | putative recombinase A | - | 0.61 | 0.77 |
| BCAL0954 | RecX family regulatory protein | - | - | 0.71 |
| BCAL0959 | putative type IV fimbrial pilin protein | - | - | -0.91 |
| BCAL0965 | putative hydrolase | - | - | -0.70 |
| BCAL0966 | conserved hypothetical protein | - | - | -0.70 |
| BCAL0980 | molybdopterin-guanine dinucleotide biosynthesis protein A 2 | - | - | -0.82 |
| BCAL1040 | glycosyl transferases group 1 protein | - | - | 0.59 |
| BCAL1044 | GntR family regulatory protein | - | - | -0.89 |
| BCAL1069 | putative cyclic-di-GMP signaling protein | 1.02 | - | 0.98 |
| BCAL1076 | putative exported protein | - | - | -2.87 |
| BCAL1091 | putative ABC transporter membrane protein | - | - | 0.92 |
| BCAL1092 | putative ABC transporter extracellular solute-binding protein | 1.29 | - | - |
| BCAL1095 | putative membrane protein | - | - | -1.43 |
| BCAL1105 | putative exported protein | -1.35 | - | - |
| BCAL1129 | hypothetical protein | - | - | 0.59 |
| BCAL1130 | conserved hypothetical protein | - | - | 0.65 |
| BCAL1136 |  | - | -1.24 | -1.66 |
| BCAL1213 | 2-oxoisovalerate dehydrogenase beta subunit | - | - | -1.00 |
| BCAL1214 | lipoamide acyltransferase component of branched- chain alpha-keto acid dehydrogenase complex | - | - | -1.23 |
| BCAL1215 | dihydrolipoamide dehydrogenase | - | - | -1.26 |
| BCAL1229 | putative lipoprotein | - | - | 0.71 |
| BCAL1236 | putative exported protein | - | - | 0.91 |
| BCAL1249 | putative PHB depolymerase | - | -1.45 | -1.54 |
| BCAL1263 | transcription elongation factor | - | - | 0.65 |
| BCAL1267 | FtsH endopeptidase | - | - | -0.75 |
| BCAL1282 | putative membrane protein | - | -0.77 | -0.66 |
| BCAL1305 | conserved hypothetical protein | -1.39 | - | - |
| BCAL1311 | putative SNF2-related helicase (pseudogene) | -2.40 | - | -1.41 |
| BCAL1315 | conserved hypothetical protein | -1.72 | - | - |
| BCAL1316 | conserved hypothetical protein | -5.09 | - | - |
| BCAL1317 | putative phage integrase | -5.15 | - | -4.37 |
| BCAL1338 | conserved hypothetical protein | - | - | -1.35 |
| BCAL1351 | putative exported protein | - | - | -1.95 |
| BCAL1372 | putative exported protein | - | - | -0.81 |
| BCAL1390 | endoglucanase precursor | 1.03 | - | - |
| BCAL1391 | putative cellulose biosynthesis protein | 1.50 | -0.75 | 0.89 |
| BCAL1395 | putative cellulose synthase catalytic subunit | 1.29 | -0.90 | 1.20 |
| BCAL1396 | putative membrane protein | - | -0.84 | - |
| BCAL1418 | Major Facilitator Superfamily protein | - | - | -0.94 |
| BCAL1423 | ABC transporter ATP-binding protein | - | - | 0.82 |
| BCAL1442 | conserved hypothetical protein | - | - | -0.95 |
| BCAL1452 | putative methyl-accepting chemotaxis protein | - | - | 0.70 |
| BCAL1464 | putative membrane protein | -1.08 | - | - |
| BCAL1475 | putative polysaccharide deacetylase | - | -1.09 | -1.06 |
| BCAL1482 | translation initiation factor IF-3 | - | - | 0.65 |
| BCAL1499 | putative exported protein | -1.03 | - | - |
| BCAL1522 | putative exported heme utilisation related protein | - | - | -0.66 |
| BCAL1523 | putative lipoprotein | -3.36 | - | -4.58 |
| BCAL1524 | putative lipoprotein | -3.50 | - | -2.15 |
| BCAL1525a | putative flp type pilus leader peptidase | - | 0.72 | - |
| BCAL1546 | putative sulfate transporter membrane protein | -2.50 | - | -4.13 |
| BCAL1562 | putative phage-related protein | - | 1.23 | - |
| BCAL1577 | hypothetical phage protein | - | - | 0.64 |
| BCAL1591 | putative phage DNA transposition protein | - | - | -4.45 |
| BCAL1635a | putative exported protein | 2.38 | - | 1.84 |
| BCAL1649 | putative membrane protein | - | 0.89 | 0.91 |
| BCAL1650 | conserved hypothetical protein | - | 1.19 | 1.41 |
| BCAL1651 | LexA repressor | - | 1.07 | 1.38 |
| BCAL1677 | putative type-1 fimbrial protein | - | - | -1.86 |
| BCAL1680 | putative type-1 fimbrial protein | - | - | -0.85 |
| BCAL1681 | putative exported protein | - | - | -1.07 |
| BCAL1687 | putative exported protein | - | - | 0.86 |
| BCAL1688 | putative RNA polymerase sigma factor | - | - | 0.93 |
| BCAL1691 | putative iron transport-related ATP-binding protein | - | - | 0.69 |
| BCAL1692 | putative iron transport-related membrane protein | - | - | 1.35 |
| BCAL1722 | putative exported chitinase | 1.75 | - | 1.71 |
| BCAL1739 | conserved hypothetical protein | - | 0.60 | - |
| BCAL1752 | conserved hypothetical protein | - | - | 0.99 |
| BCAL1753 | LysR family regulatoy protein | - | - | 0.75 |
| BCAL1786 | putative lipoprotein | - | - | -1.82 |
| BCAL1796 | putative saccharopine dehydrogenase | -2.20 | - | - |
| BCAL1801 | putative membrane protein | - | - | 0.69 |
| BCAL1828 | putative fimbrial usher protein | - | - | -5.25 |
| BCAL1829 | putative outer membrane protein | - | - | -2.26 |
| BCAL1832 | conserved hypothetical protein | - | -0.93 | -0.60 |
| BCAL1845 | putative membrane protein | - | - | -0.65 |
| BCAL1910 | acetoin:2.6-dichlorophenolindophenol oxidoreductase beta subunit | - | - | -0.83 |
| BCAL1952 | conserved hypothetical protein | -1.55 | - | -0.84 |
| BCAL1988 | putative D-amino acid dehydrogenase small subunit | - | - | 0.76 |
| BCAL2027 | conserved hypothetical protein | - | -0.87 | -0.76 |
| BCAL2046 | conserved hypothetical protein | - | - | -0.67 |
| BCAL2064 | putative membrane protein | - | - | -0.72 |
| BCAL2139 | two-component regulatory system, response regulator | - | - | -0.68 |
| BCAL2141 | cytochrome O ubiquinol oxidase protein | - | - | -1.76 |
| BCAL2142 | cytochrome o ubiquinol oxidase subunit III | - | - | -1.25 |
| BCAL2194 | chaperone protein HscA homolog | - | - | 0.65 |
| BCAL2206 | phasin-like protein | 1.23 | - | - |
| BCAL2252 | putative DNA photolyase | - | - | 0.81 |
| BCAL2269 | putative membrane protein | - | - | 0.67 |
| BCAL2270 | conserved hypothetical protein | - | - | 0.68 |
| BCAL2279 | conserved hypothetical protein (pseudogene) | - | - | -0.67 |
| BCAL2281 | putative ferrichrome receptor | 1.70 | - | 1.14 |
| BCAL2287 | putative fumarate hydratase | - | - | 0.71 |
| BCAL2288 | bacterioferritin | - | - | 0.76 |
| BCAL2290 | putative bacterioferritin ferredoxin protein | - | - | 0.76 |
| BCAL2352 | putative carbonic anhydrase | - | - | 1.01 |
| BCAL2383 | potassium-transporting ATPase A chain | - | - | -1.01 |
| BCAL2391 | putative nicotinate-nucleotide adenylyltransferase | - | 0.61 | 0.69 |
| BCAL2426 | putative membrane protein | - | - | -1.84 |
| BCAL2439 | putative membrane protein | - | - | -0.63 |
| BCAL2464 | short chain dehydrogenase | - | - | -0.83 |
| BCAL2466 | ecotin precursor | - | - | 0.63 |
| BCAL2476 | hypothetical protein | - | - | 0.74 |
| BCAL2476a | conserved hypothetical protein (fragment) | - | 0.66 | - |
| BCAL2512 | putative transmembrane protein | - | - | -2.05 |
| BCAL2519 | putative O-antigen acetylase | - | - | -1.20 |
| BCAL2529 | putative transcriptional regulator | - | - | -1.40 |
| BCAL2559 | putative transcriptional regulator | - | - | -1.97 |
| BCAL2560 | putative DNA-binding protein | - | - | -1.25 |
| BCAL2561 | putative LysR transcriptional regulatory protein | - | -0.69 | - |
| BCAL2573 | putative ABC transporter ATP-binding protein | 1.72 | - | - |
| BCAL2604 | conserved hypothetical protein | - | -0.64 | - |
| BCAL2605 | two-component regulatory system,. sensor kinase | - | - | 0.65 |
| BCAL2606 | two-component regulatory system,. response regulator | - | - | 0.66 |
| BCAL2607 | putative exported protein | - | - | 0.75 |
| BCAL2613 | putative acetyltransferase | - | - | 0.61 |
| BCAL2635 | putative exported protein | - | -1.15 | -1.29 |
| BCAL2636 | putative fimbriae chaperone | - | -1.02 | -1.31 |
| BCAL2637 | putative fimbriae usher protein | - | - | -0.92 |
| BCAL2645 | putative OmpA family transmembrane protein | - | -0.60 | -0.66 |
| BCAL2698 | putative membrane protein | -1.17 | - | - |
| BCAL2701 | aminotransferase class-III | - | - | 0.63 |
| BCAL2738 | putative exported protein | - | - | 0.62 |
| BCAL2746 | putative citrate synthase | - | - | -0.74 |
| BCAL2757 | superoxide dismutase SodB | - | - | 0.66 |
| BCAL2793 | Major Facilitator Superfamily protein | - | - | -0.76 |
| BCAL2799 | putative carbohydrate kinase | - | - | 0.65 |
| BCAL2813 | putative periplasmic solute-binding protein | - | - | 0.60 |
| BCAL2817 | S-(hydroxymethyl)glutathione dehydrogenase | - | - | 0.62 |
| BCAL2818 | putative sugar kinase protein | - | - | 0.82 |
| BCAL2820 | outer membrane efflux protein | -5.70 | - | -6.81 |
| BCAL2821 | RND family efflux system transporter protein | -5.42 | - | -4.86 |
| BCAL2822 | RND family efflux system transporter protein | -3.01 | - | -2.70 |
| BCAL2834 | putative acylhydrolase | - | - | 0.69 |
| BCAL2847 | Putative methionine aminopeptidase | - | - | 0.70 |
| BCAL2848 | conserved hypothetical protein | - | - | 0.60 |
| BCAL2904 | conserved hypothetical protein | 1.66 | - | 1.36 |
| BCAL2962a | hypothetical protein | - | - | 0.62 |
| BCAL2975 | possible regulatory protein | - | -1.46 | -1.82 |
| BCAL2976 | NAD-dependent formate dehydrogenase gamma subunit | - | - | -0.72 |
| BCAL2977 | NAD-dependent formate dehydrogenase beta subunit | - | -0.87 | -1.05 |
| BCAL2978 | NAD-dependent formate dehydrogenase alpha subunit | - | -1.28 | -1.56 |
| BCAL2979 | NAD-dependent formate dehydrogenase delta subunit | - | -1.53 | -1.63 |
| BCAL2998 | transglycosylase associated protein | - | - | -1.08 |
| BCAL3003 | hypothetical protein | -1.81 | - | - |
| BCAL3008 | putative outer membrane porin protein | -1.27 | - | - |
| BCAL3071 | putative nitrogen assimilation regulatory protein Nac | - | - | -0.69 |
| BCAL3081 | hypothetical protein | - | - | 0.76 |
| BCAL3111 | conserved hypothetical protein | - | -0.78 | - |
| BCAL3148 | polyketide cyclase / dehydrase and lipid transport family | -2.46 | - | -2.67 |
| BCAL3149 | outer membrane lipoprotein carrier protein LolA | -2.61 | - | -3.20 |
| BCAL3150 | putative exported protein | -2.71 | -1.45 | -4.21 |
| BCAL3151 | putative transmembrane anti-sigma factor | -2.15 | -1.52 | -2.59 |
| BCAL3152 | putative RNA polymerase sigma factor, ECF subfamily | -4.37 | -1.43 | -3.96 |
| BCAL3153 | putative lipoprotein | -6.00 | -1.88 | -4.28 |
| BCAL3185 | 4-hydroxybenzoate transporter | - | - | -0.81 |
| BCAL3214 | carboxymuconolactone decarboxylase family | - | -0.59 | - |
| BCAL3227 | conserved hypothetical protein | - | - | 0.93 |
| BCAL3258 | tetracycline repressor protein | -1.22 | -0.99 | -1.00 |
| BCAL3259_J_0 | tetA, pseudogene | -1.84 | -2.08 | -1.93 |
| BCAL3259_J_1 | tetA, pseudogene | -1.27 | -1.43 | -1.40 |
| BCAL3263 | conserved hypothetical protein | - | - | 0.64 |
| BCAL3282 | putative phospho-2-dehydro-3-deoxyheptonate polyphosphatase | - | - | 0.62 |
| BCAL3297 | putative ferritin DPS-family DNA binding protein | - | -1.01 | - |
| BCAL3298 | conserved hypothetical protein | - | -0.71 | - |
| BCAL3311 | putative exported protein | - | - | 0.65 |
| BCAL3312 | putative cytochrome b-561 membrane protein | - | - | 0.83 |
| BCAL3353 | putative outer membrane autotransporter | -2.79 | - | -4.25 |
| BCAL3380 | putative allantoicase | - | - | -0.61 |
| BCAL3473 | outer membrane porin | - | 0.60 | - |
| BCAL3501 | flagellar biosynthetic protein FliR | 2.71 | - | 1.47 |
| BCAL3503 | flagellar biosynthetic protein FliP precursor | 1.07 | - | - |
| BCAL3505 | flagellar motor switch protein FliN | 3.13 | - | 1.83 |
| BCAL3506 | flagellar motor switch protein FliM | 2.58 | - | - |
| BCAL3507 | flagellar basal body-associated protein FliL | 1.68 | - | 1.28 |
| BCALr0949 | tRNA Met anticodon CAT. Cove score 88.36 | - | - | -1.87 |
| BCALr0970a | tRNA Asn anticodon GTT. Cove score 85.87 | - | - | -3.20 |
| BCALr1551a | tRNA Leu anticodon CAG. Cove score 72.78 | - | - | -1.63 |
| BCALr1614 | tRNA Met anticodon CAT. Cove score 86.01 | - | - | -0.77 |
| BCALr2125c | tRNA Asp anticodon GTC. Cove score 90.27 | - | - | -2.34 |
| BCALr2125e | tRNA Asp anticodon GTC. Cove score 95.34 | - | - | -2.13 |
| BCALr2219 | tRNA Met anticodon CAT. Cove score 86.80 | - | - | -0.98 |
| BCAM0007 | putative phage integrase | - | 0.70 | 1.05 |
| BCAM0008 | conserved hypothetical protein | - | 0.86 | 1.12 |
| BCAM0030 | conserved hypothetical protein | - | - | 0.96 |
| BCAM0031 | conserved hypothetical protein | - | - | 0.96 |
| BCAM0034 | putative short-chain dehydrogenase | - | - | -1.71 |
| BCAM0069 | conserved hypothetical protein | - | 0.59 | 0.71 |
| BCAM0071 | puatative mandelate racemase/muconate lactonizing enzyme | - | 0.61 | - |
| BCAM0072 | putative thiamine pyrophosphate enzyme | - | 0.61 | - |
| BCAM0073 | hypothetical protein | - | - | 0.60 |
| BCAM0080 | putative AMP-binding enzyme | - | - | -4.01 |
| BCAM0095 | AraC family regulatory protein | - | - | 0.79 |
| BCAM0098 | putative ABC transporter system permease | 1.55 | - | - |
| BCAM0163 | putative microcin immunity protein | 2.67 | - | 0.68 |
| BCAM0186 | lectin | - | - | 1.56 |
| BCAM0187 | putative 2-isopropylmalate synthase | - | - | 0.99 |
| BCAM0188 | putative autoinducer - LuxR family | - | - | 0.61 |
| BCAM0189 | putative AraC-family regulatory protein | - | - | 0.61 |
| BCAM0191 | putative non-ribosomal peptide synthetase | - | - | 0.92 |
| BCAM0192 | conserved hypothetical protein | - | - | 1.43 |
| BCAM0193 | conserved hypothetical protein | - | - | 1.42 |
| BCAM0194 | conserved hypothetical protein | 2.07 | - | 1.59 |
| BCAM0195 | putative non-ribosomal peptide synthetase | - | - | 1.54 |
| BCAM0196 | conserved hypothetical protein | - | - | 0.80 |
| BCAM0232 | conserved hypothetical protein | - | - | -0.78 |
| BCAM0275a | conserved hypothetical protein | - | -1.33 | -1.59 |
| BCAM0276 | putative universal stress protein | - | -1.67 | -1.82 |
| BCAM0277 | conserved hypothetical protein | - | -1.87 | -1.88 |
| BCAM0278 | putative heat shock protein | - | -3.27 | -3.69 |
| BCAM0279 | putative nitroreductase | - | -1.52 | -1.56 |
| BCAM0280 | putative phospholipid-binding protein | - | -2.99 | -3.37 |
| BCAM0280A | conserved hypothetical protein | - | -2.33 | -2.67 |
| BCAM0281 | putative sulfate transporter family protein | - | -1.07 | -1.08 |
| BCAM0283 | putative lysine decarboxylase | - | - | -1.86 |
| BCAM0284 | putative cytochrome c | - | - | -2.01 |
| BCAM0285 | conserved hypothetical protein | - | - | -1.50 |
| BCAM0286 | putative alcohol dehydrogenase | - | -1.57 | -1.50 |
| BCAM0289 | putative histdine kinase | - | - | -2.45 |
| BCAM0290 | putative universal stress protein | - | -2.27 | -2.43 |
| BCAM0291 | putative universal stress protein | - | -2.18 | -2.55 |
| BCAM0292 | putative universal stress protein | - | -2.04 | -2.43 |
| BCAM0293 | putative acetate kinase | - | - | -2.31 |
| BCAM0294 | putative universal stress protein | - | -2.24 | -2.54 |
| BCAM0295 | conserved hypothetical protein | - | -2.24 | -2.51 |
| BCAM0296 | acetoacetyl-CoA reductase | - | - | -2.56 |
| BCAM0297 | putative polymerase | - | - | -2.55 |
| BCAM0298 | putative phosphate acetyl/butyryl transferase | - | -2.83 | -3.34 |
| BCAM0299 | putative zinc-binding alcoholdehydrogenase | - | -3.21 | -3.74 |
| BCAM0300 | putative metallo-beta-lactamase family protein | - | - | -1.66 |
| BCAM0301 | putative membrane protein | - | -1.23 | -1.40 |
| BCAM0302 | putative ABC transporter protein | - | - | -0.77 |
| BCAM0303 | putative ABC transporter ATP-binding protein | - | -2.40 | -2.98 |
| BCAM0304 | putative secretion protein - HlyD family | - | - | -1.63 |
| BCAM0305 | putative outer membrane efflux protein | - | -1.73 | -1.86 |
| BCAM0306 | putative membrane protein | - | -2.48 | -2.86 |
| BCAM0307 | conserved hypothetical protein | - | -1.83 | -2.04 |
| BCAM0308 | conserved hypothetical protein | - | -2.12 | -2.50 |
| BCAM0309 | putative cell division-related metallo peptidase | - | -2.37 | -2.69 |
| BCAM0311 | putative 6-phosphofructokinase | - | -1.82 | -2.01 |
| BCAM0312 | putative polysaccharide deacetylase | - | -1.49 | -1.72 |
| BCAM0313 | putative exported protein | - | -1.49 | -1.68 |
| BCAM0315 | putative exported protein | - | - | -1.33 |
| BCAM0316 | conserved hypothetical protein | - | -1.39 | -1.54 |
| BCAM0317 | putative membrane protein | - | -1.81 | -2.13 |
| BCAM0318 | putative cation-transporting ATPase | - | -1.16 | -1.40 |
| BCAM0319 | putative universal stress protein | - | -1.35 | -1.27 |
| BCAM0374 | conserved hypothetical protein | - | - | 0.62 |
| BCAM0384 | putative lipoprotein | - | -1.61 | -1.81 |
| BCAM0416 | putative agmatinase | - | - | -0.97 |
| BCAM0429 | conserved hypothetical protein | - | 1.08 | 0.96 |
| BCAM0453 | putative exported protein | - | - | -0.93 |
| BCAM0456 | putative ThiJ/PfpI family protein | - | - | -1.24 |
| BCAM0481 | AraC family regulatory protein | - | -0.63 | - |
| BCAM0495 | putative DNA-binding protein | -1.40 | - | - |
| BCAM0502 | conserved hypothetical protein | - | - | -0.70 |
| BCAM0540 | putative serine acetyltransferase | 1.70 | - | - |
| BCAM0542 | conserved hypothetical protein | - | - | 0.63 |
| BCAM0608 | FAD dependent oxidoreductase | - | - | -1.39 |
| BCAM0633 | conserved hypothetical protein | - | - | 0.85 |
| BCAM0634 | hypothetical protein | - | - | 1.65 |
| BCAM0637 | Major Facilitator Superfamily protein | - | - | 0.60 |
| BCAM0641 | putative amidase | - | - | 0.71 |
| BCAM0670 | putative ABC transporter system permease | - | - | -1.04 |
| BCAM0688 | conserved hypothetical protein | - | - | 0.74 |
| BCAM0694 | putative membrane protein | -1.44 | - | -2.04 |
| BCAM0695 | putative lipoprotein | -7.26 | - | -6.75 |
| BCAM0721 | O-acetylhomoserine (thiol)-lyase | - | - | 0.69 |
| BCAM0725 | conserved hypothetical protein | 1.06 | 0.76 | 0.76 |
| BCAM0726 | conserved hypothetical protein | 1.57 | 1.71 | 1.53 |
| BCAM0727 | conserved hypothetical protein | 1.46 | 1.24 | 1.43 |
| BCAM0731 | putative transcriptional activator - MarR | - | - | 0.85 |
| BCAM0746 | argininosuccinate synthase | - | - | 0.67 |
| BCAM0753 | putative membrane protein | - | - | -0.69 |
| BCAM0776 | putative cNMP-binding domain protein | 3.22 | - | 1.17 |
| BCAM0777 | putative flagellar motor proton channel | 1.38 | - | - |
| BCAM0778 | putative flagellar motor protein | 1.73 | - | - |
| BCAM0800 | conserved hypothetical protein | - | 0.77 | 1.03 |
| BCAM0801 | putative transcriptional regulator - LysR | - | - | 0.59 |
| BCAM0810 | putative aromatic oxygenase | -1.35 | - | - |
| BCAM0811 | putative aromatic oxygenase | -2.45 | - | - |
| BCAM0838 | hypothetical protein | - | 0.62 | - |
| BCAM0854 | bifunctional exopolysaccharide biosynthesis protein | - | - | -1.00 |
| BCAM0901 | putative AMP nucleosidase | - | - | 0.60 |
| BCAM0934 | putative secretion protein - HlyD family | - | - | 0.96 |
| BCAM0943 | conserved hypothetical protein | - | -0.60 | - |
| BCAM0946 | putative cytochrome b561 | - | - | -0.69 |
| BCAM0948 | putative TonB dependent receptor | - | - | 1.06 |
| BCAM0950 | lipase chaperone | - | - | 0.96 |
| BCAM0965 | malate dehydrogenase | - | - | 0.78 |
| BCAM0987 | putative flagellar basal body rod protein | 1.84 | - | - |
| BCAM1110 | putative MFS family sugar transporter protein | 2.58 | - | - |
| BCAM1111 | ornithine decarboxylase | - | - | -1.88 |
| BCAM1112 | biodegradative arginine decarboxylase | - | - | -1.57 |
| BCAM1113 | putrescine transport protein | - | - | -1.41 |
| BCAM1125 | putative ABC transporter system permease | - | - | -2.27 |
| BCAM1143 | putative alpha/beta hydrolase fold protein | - | - | -1.36 |
| BCAM1151 | methylmalonate-semialdehyde dehydrogenase | - | - | -0.77 |
| BCAM1152 | putative MFS family sugar transporter protein | - | - | -0.88 |
| BCAM1167 | conserved hypothetical protein | - | - | 0.64 |
| BCAM1226 | glycosyl transferase | - | - | -1.07 |
| BCAM1279 | conserved hypothetical protein | - | 0.86 | - |
| BCAM1283 | putative phosphoesterase | - | - | -3.43 |
| BCAM1316a | conserved hypothetical protein | - | - | 1.04 |
| BCAM1316b | conserved hypothetical protein | - | - | 1.00 |
| BCAM1344 | conserved hypothetical protein | - | - | -0.95 |
| BCAM1349 | putative regulatory protein | - | - | -0.61 |
| BCAM1351 | putative regulatory protein | - | -1.54 | -1.64 |
| BCAM1352 | putative phosphoesterase | -1.47 | - | -2.46 |
| BCAM1413a | conserved hypothetical protein | - | - | 0.62 |
| BCAM1421 | putative multidrug efflux protein | - | - | 0.68 |
| BCAM1423 | putative AMP-binding enzyme | - | - | -1.28 |
| BCAM1424 | methyl-accepting chemotaxis protei | 3.56 | - | 3.44 |
| BCAM1444 | putative membrane protein | - | 0.85 | - |
| BCAM1455 | putative porin | - | - | -0.65 |
| BCAM1480 | conserved hypothetical protein | - | -1.29 | -1.13 |
| BCAM1481 | conserved hypothetical protein | - | -1.47 | -1.63 |
| BCAM1482 | conserved hypothetical protein | - | -1.58 | -1.88 |
| BCAM1484 | putative response regulator protein | - | 0.58 | 1.18 |
| BCAM1491 | putative exported protein | -1.21 | - | - |
| BCAM1495 | putative universal stress protein | - | -1.43 | -1.58 |
| BCAM1496 | conserved hypothetical protein | - | - | -1.20 |
| BCAM1502 | conserved hypothetical protein | - | - | -1.78 |
| BCAM1503 | putative methyl-accepting chemotaxis protein | 1.87 | - | - |
| BCAM1529 | putative hydrolase | - | - | -1.01 |
| BCAM1555 | conserved hypothetical protein | -2.11 | - | - |
| BCAM1569 | putative BNR/Asp-box protein | - | - | -0.74 |
| BCAM1570 | alcohol dehydrogenase | - | -1.69 | -1.87 |
| BCAM1577 | FAD-dependent oxidoreductase | - | - | -0.97 |
| BCAM1613 | putative hydratase/decarboxylase | - | - | -1.30 |
| BCAM1619 | putative DNA-binding cold-shock protein | - | 0.66 | - |
| BCAM1635 | putative CoA transferase | - | - | -1.28 |
| BCAM1657 | putative exported protein | - | - | -1.67 |
| BCAM1675 | conserved hypothetical protein | - | - | 0.61 |
| BCAM1696 | putative lipoprotein | - | - | -0.87 |
| BCAM1697 | putative membrane-associated amino terminal protease | - | 0.76 | -0.64 |
| BCAM1732 | putative porin | - | - | -0.90 |
| BCAM1744 | putative exported peptidase | 3.40 | - | 3.91 |
| BCAM1756 | putative molybdopterin oxidoreductase | - | 0.73 | 0.87 |
| BCAM1757 | putative membrane protein | - | - | 0.64 |
| BCAM1775 | putative transglycosylase associated protein | - | - | 0.59 |
| BCAM1777A | putative exported protein | 2.00 | -1.19 | 0.86 |
| BCAM1780 | peptidoglycan-binding LysM:peptidase m23b precursor | 2.48 | - | 1.77 |
| BCAM1799 | putative prophage protein | - | 0.64 | 0.59 |
| BCAM1804 | methyl-accepting chemotaxis protein | 3.29 | - | 2.93 |
| BCAM1812 | putative arginase | - | - | -0.59 |
| BCAM1828 | putative amidase | - | - | -0.94 |
| BCAM1829 | putative universal stress protein | - | -0.85 | -1.31 |
| BCAM1876 | hypothetical phage protein | 2.25 | - | 0.76 |
| BCAM1881 | hypothetical phage protein | - | 0.72 | 0.64 |
| BCAM1884 | putative DNA-binding phage protein | - | - | 0.73 |
| BCAM1948 | MerR-family transcriptional regulator | - | -3.91 | -4.32 |
| BCAM1960 | calcineurin-like phosphoesterase | - | - | -0.61 |
| BCAM1998 | two-component response regulator protein | - | - | -3.24 |
| BCAM2000 | conserved hypothetical protein | - | - | 0.68 |
| BCAM2019 | putative AraC-family transcriptional regulator | - | -0.69 | -0.88 |
| BCAM2024 | putative membrane protein | 4.57 | - | 2.63 |
| BCAM2031 | putative exported protein | - | - | -1.69 |
| BCAM2039 | putative transcriptional regulator | -4.91 | 0.77 | -4.52 |
| BCAM2042 | type III secretion system protein | - | - | -0.69 |
| BCAM2043_J_0 | putative type III secretion system protein (pseudogene) | - | - | -0.76 |
| BCAM2045 | type III secretion system protein | - | - | -1.08 |
| BCAM2057 | type III secretion system protein | - | - | -0.59 |
| BCAM2067 | putative undecaprenyl pyrophosphate synthetase | - | - | 0.60 |
| BCAM2084 | conserved hypothetical protein | - | - | 0.65 |
| BCAM2086 | putative spermidine synthase | - | - | 0.61 |
| BCAM2087 | putative lipoprotein | - | - | 0.91 |
| BCAM2159 | conserved hypothetical exported protein | - | - | -0.69 |
| BCAM2167 | conserved hypothetical protein | - | -1.39 | -1.44 |
| BCAM2191 | enoyl-CoA hydratase/isomerase family protein | -1.80 | - | -2.32 |
| BCAM2192 | enoyl-CoA hydratase/isomerase family protein | - | - | -2.86 |
| BCAM2193 | putative 3-hydroxyisobutyrate dehydrogenase | - | - | -1.58 |
| BCAM2194 | methylmalonate-semialdehyde dehydrogenase | - | - | -1.68 |
| BCAM2195 | putative AMP-binding enzyme | - | - | -1.63 |
| BCAM2199 | putative transmembrane protein | - | - | -0.89 |
| BCAM2203 | putative membrane protein | - | - | -2.48 |
| BCAM2207 | conserved hypothetical protein | - | - | -0.92 |
| BCAM2209 | conserved hypothetical protein | - | -0.88 | -0.92 |
| BCAM2210 | putative transmembrane protein | - | - | -0.69 |
| BCAM2233 | putative pyochelin biosynthetic protein PchC | - | - | 0.71 |
| BCAM2234 | putative pyochelin biosynthetic protein PchB | - | - | 0.64 |
| BCAM2304 | putative amine dehydrogenase | -3.63 | - | -5.35 |
| BCAM2305 | putative AraC family transcriptional protein | - | - | -1.22 |
| BCAM2333 | putative glutathione-independent formaldehyde dehydrogenase | -1.86 | - | - |
| BCAM2334 | putative multidrug resistance protein – HlyD family | -2.37 | - | -1.93 |
| BCAM2335 | putative RND family efflux transporter | - | - | -0.80 |
| BCAM2336 | putative sugar transferase | -2.57 | - | -3.70 |
| BCAM2337 | putative transmembrane transport protein | -2.50 | - | -4.41 |
| BCAM2338 | putative glycosyltransferase | -3.95 | - | -5.84 |
| BCAM2339 | putative transmembrane methyltransferase | -1.93 | - | - |
| BCAM2340 | putative fatty acid biosynthetic protein | -3.06 | - | -2.64 |
| BCAM2374 | putative methyl-accepting chemotaxis protein | 1.45 | - | - |
| BCAM2377 | conserved hypothetical exported protein | 2.53 | - | - |
| BCAM2400a | conserved hypothetical exported protein | - | -1.43 | -2.28 |
| BCAM2400b | putative exported protein | -1.67 | -1.50 | -3.13 |
| BCAM2409 | putative glycine-betaine binding ABC transporter protein | - | -1.54 | -1.64 |
| BCAM2418 | putative haemagglutinin-related autotransporter protein | - | - | -0.90 |
| BCAM2419 | putative outer membrane protein A precursor | - | - | -2.59 |
| BCAM2425 | conserved hypothetical protein | -1.99 | - | -0.78 |
| BCAM2426 | putative diguanylate phosphodiesterase | -2.28 | - | -3.02 |
| BCAM2434 | putative TetR family transcriptional regulator | - | - | -2.18 |
| BCAM2462 | putative gram-negative porin | - | - | -0.65 |
| BCAM2508 | putative membrane protein | - | - | -0.94 |
| BCAM2549 | putative RND family transporter | - | - | 0.87 |
| BCAM2551 | putative secretion protein - HlyD family | - | - | 0.92 |
| BCAM2552 | putative hydrolase | - | 0.80 | 1.12 |
| BCAM2561 | putative 4-aminobutyrate aminotransferase | 1.44 | - | - |
| BCAM2562 | putative succinate-semialdehyde dehydrogenase | 1.48 | - | - |
| BCAM2564 | putative aerotaxis receptor | 3.38 | - | 1.14 |
| BCAM2596 | putative beta-lactamase | - | - | -1.15 |
| BCAM2616 | putative AraC family transcriptional regulator | -1.97 | - | -2.10 |
| BCAM2621_J_0 | putative porin-related protein (pseudogene) | - | -0.94 | -1.10 |
| BCAM2621_J_1 | putative porin-related protein (pseudogene) | - |  | -1.06 |
| BCAM2625 | conserved hypothetical protein | - | - | -0.88 |
| BCAM2665 | putative MFS family transporter | - | - | 0.63 |
| BCAM2674 | putative cytochrome oxidase subunit I | - | 0.61 | - |
| BCAM2676 | conserved hypothetical membrane protein | - | - | -0.64 |
| BCAM2685 | conserved hypothetical protein | - | -1.38 | -1.37 |
| BCAM2689 | putative methyl-accepting chemotaxis protein | 1.19 | - | 0.92 |
| BCAM2700 | putative membrane protein | - | - | -0.63 |
| BCAM2701 | aconitate hydratase 1 | - | - | -0.84 |
| BCAM2708 | IclR family regulatory protein | - | - | 0.75 |
| BCAM2710 | putative acetyl-CoA synthetase | - | -1.19 | - |
| BCAM2720 | putative phospholipase C precursor | - | - | -2.08 |
| BCAM2732 | putative membrane protein | - | - | 0.60 |
| BCAM2746 | carbon starvation protein A | - | - | 0.80 |
| BCAM2758 | cblS, two-component regulatory system sensor kinase protein | 1.33 | - | - |
| BCAM2759 | cblD, putative minor pilin and initiator | 1.58 | - | - |
| BCAM2760 | cblC, putative outer membrane usher | 1.40 | - | - |
| BCAM2771 | putative dihydrodipicolinate synthetase | - | - | -1.26 |
| BCAM2836 | putative diguanylate cyclase | 1.25 | - | 1.11 |
| BCAM2837_J_0 | putative response regulator( pseudogene) | 1.65 | - | 1.73 |
| BCAM2837_J_1 | putative response regulator (pseudogene) | 1.81 | - | 1.64 |
| BCAMr0727 | tRNA Pseudo anticodon GAA. Cove score 36.58 | 1.55 | - | 1.12 |
| BCAS0036 | putative transcriptional regulator - LysR family | -2.16 | - | - |
| BCAS0041 | radical SAM superfamily protein | - | - | -2.46 |
| BCAS0044 | putative phosphoribosylglycinamide synthetase | - | 0.70 | - |
| BCAS0051 | putative glycerol utilisation-related protein | - | - | 0.59 |
| BCAS0055 | putative transporter - LysE family | - | - | -0.75 |
| BCAS0060 | extracellular amino acid-binding protein | - | - | 0.66 |
| BCAS0151 | hypothetical protein | 2.04 | - | - |
| BCAS0152 | putative hydrolase | 1.59 | - | - |
| BCAS0194 | conserved hypothetical protein | - | -0.60 | - |
| BCAS0216 | putative acyl carrier protein | - | - | 0.72 |
| BCAS0236 | putative haemagglutinin-related autotransporter protein | - | - | -1.92 |
| BCAS0239 | putative excinuclease ABC subunit A family protein | - | 0.83 | 1.15 |
| BCAS0292 | conserved hypothetical protein | - | - | 1.70 |
| BCAS0293 | nematocidal protein AidA | - | - | 1.84 |
| BCAS0319 | putative oxidoreductase, may form heterodimer with product of BCAS0320 | -2.18 | - | -3.05 |
| BCAS0320 | isoquinoline 1-oxidoreductase alpha subunit | -3.77 | - | -4.32 |
| BCAS0321 | hypothetical glycine-rich autotransporter protein | -2.61 | - | -2.04 |
| BCAS0321b | hypothetical glycine-rich autotransporter protein | - | - | -1.95 |
| BCAS0352 | AraC family regulatory protein | - | - | -0.94 |
| BCAS0353 | putative aldose 1-epimerase | - | - | -4.11 |
| BCAS0374 | Major Facilitator Superfamily protein | - | - | -3.58 |
| BCAS0397 | metallo peptidase, subfamily M20D | 1.63 | - | - |
| BCAS0398 | putative diguanylate cyclase | 1.68 | - | - |
| BCAS0472 | putative multidrug resistance transporter protein | - | - | -0.94 |
| BCAS0496 | benzoate 1.2-dioxygenase alpha subunit | - | - | -0.67 |
| BCAS0504 | putative phage transmembrane acetyltransferase | - | 0.59 | - |
| BCAS0505a | hypothetical phage protein | 2.10 | - | 1.38 |
| BCAS0506 | putative phage tail protein gpI | 1.81 | 0.85 | 1.77 |
| BCAS0507 | putative phage baseplate assembly protein gpJ | 1.59 | 0.78 | 1.77 |
| BCAS0508 | putative phage baseplate protein gpW | 1.45 | 0.81 | 1.90 |
| BCAS0509 | putative phage baseplate assembly protein gpV | - | - | 0.63 |
| BCAS0510 | hypothetical phage protein | - | - | 1.46 |
| BCAS0512 | putative phage protein gpU | - | - | 1.20 |
| BCAS0513 | putative phage tail protein | 1.29 | - | 1.38 |
| BCAS0515 | hypothetical phage protein | - | 1.10 | 2.39 |
| BCAS0516 | hypothetical phage protein | 2.40 | 1.05 | 2.31 |
| BCAS0517 | putative phage tail tube protein | 2.36 | 0.79 | 2.12 |
| BCAS0518 | putative phage tail sheath protein | 2.23 | 1.06 | 2.24 |
| BCAS0519 | hypothetical phage protein | 2.64 | 1.06 | 2.33 |
| BCAS0520 | hypothetical phage protein | 2.95 | 0.94 | 2.37 |
| BCAS0521 | hypothetical phage protein | 2.42 | 1.03 | 2.39 |
| BCAS0522 | hypothetical phage protein | 1.85 | - | 2.07 |
| BCAS0523 | hypothetical phage protein | 2.91 | 1.25 | 2.63 |
| BCAS0524 | hypothetical phage protein | 3.01 | 0.98 | 2.39 |
| BCAS0525 | putative phage Mu G protein | - | - | 0.61 |
| BCAS0527 | hypothetical phage protein | 1.23 | - | 1.21 |
| BCAS0528 | putative phage portal protein | 1.25 | 0.64 | 1.40 |
| BCAS0529 | hypothetical phage protein | 1.23 | - | 1.05 |
| BCAS0530 | hypothetical phage protein | - | 0.85 | 1.55 |
| BCAS0531 | putative phage membrane protein | 1.52 | 0.62 | 1.56 |
| BCAS0532 | putative phage exported protein Rz | 1.32 | - | 1.73 |
| BCAS0534 | putative phage membrane protein | 1.21 | - | 0.92 |
| BCAS0540 | hypothetical phage protein | 1.17 | 0.66 | 0.76 |
| BCAS0541 | hypothetical phage protein | 1.31 | - | 0.73 |
| BCAS0542 | hypothetical phage protein | 1.36 | - | 0.82 |
| BCAS0543 | putative phage transcriptional regulator | 1.56 | 0.68 | 0.90 |
| BCAS0544 | hypothetical phage protein | 1.83 | 0.85 | 1.34 |
| BCAS0546 | putative phage integrase | 1.21 | - | - |
| BCAS0547 | putative phage DNA-binding protein | 2.26 | - | 1.34 |
| BCAS0548 | hypothetical phage protein | 2.09 | 0.98 | 1.63 |
| BCAS0549 | hypothetical phage protein | 2.01 | 0.93 | 1.47 |
| BCAS0550 | hypothetical phage protein | 1.30 | 0.80 | 1.33 |
| BCAS0551 | phage DNA-binding protein | - | - | 0.92 |
| BCAS0552 | hypothetical phage protein | 1.20 | 0.69 | 0.97 |
| BCAS0553 | hypothetical phage protein | 1.06 | - | 0.69 |
| BCAS0554 | putative phage transcriptional regulator | 1.02 | 0.59 | 0.78 |
| BCAS0576 | putative binding-protein-dependent transport system component | 1.43 | - | - |
| BCAS0617 | conserved hypothetical protein | - | - | 0.92 |
| BCAS0661B | conserved hypothetical protein | - | 0.60 | 0.63 |
| BCAS0662 | conserved hypothetical protein | - | - | 0.65 |
| BCAS0673 | conserved hypothetical protein | - | 0.84 | - |
| BCAS0677 | conserved hypothetical protein | 2.24 | - | 1.11 |
| BCAS0678 | hypothetical protein | - | - | 1.03 |
| BCAS0737 | putative acetyl-CoA acetyltransferase | - | - | -1.27 |
| BCAS0738 | putative short-chain dehydrogenase family protein | - | - | -1.22 |
| BCAS0739 | putative acetyl-CoA synthetase | - | - | -0.97 |
| BCAS0766 | multidrug efflux protein transport protein | - | - | -3.93 |
| IG1_1043076 | interG_chr1_pos_568_1043076:1043230 | - | 0.69 | - |
| IG1_1063818 | interG_chr1_pos_580_1063818:1063915 | 0,63 | - | - |
| IG1_1185171 | interG_chr1_pos_655_1185171:1185327 | - | -1.11 | - |
| IG1_1224302 | interG_chr1_pos_668_1224302:1224367 | - | - | -2.37 |
| IG1_1314636 | interG_chr1_pos_706_1314636:1319398 | - | - | -1.36 |
| IG1_1326468 | interG_chr1_pos_712_1326468:1326783 | - | - | -1.17 |
| IG1_1435033 | interG_chr1_pos_779_1435033:1437975 | -0,95 | - | - |
| IG1_147730 | interG_chr1_pos_68_147730:147909 | 3,47 | -1.76 | 2.90 |
| IG1_1527001 | interG_chr1_pos_824_1527001:1527782 | - | -0.67 | - |
| IG1_155066 | interG_chr1_pos_74_155066:155230 | 4,65 | - | 2.31 |
| IG1_1560901 | interG_chr1_pos_845_1560901:1562397 | 0,73 | - | - |
| IG1_157905 | interG_chr1_pos_77_157905:157971 | 2,69 | - | 1.36 |
| IG1_1605376 | interG_chr1_pos_871_1605376:1607218 | 0,67 | - | - |
| IG1_1787902 | interG_chr1_pos_972_1787902:1789704 | 1,24 | - | - |
| IG1_1814299 | interG_chr1_pos_989_1814299:1814586 | 1,08 | - | - |
| IG1_1836014 | interG_chr1_pos_1002_1836014:1836102 | - | - | -1.59 |
| IG1_196793 | interG_chr1_pos_107_196793:196856 | -3,17 | - | -2.54 |
| IG1_1982241 | interG_chr1_pos_1068_1982241:1984670 | -0,69 | - | - |
| IG1_1996664 | interG_chr1_pos_1072_1996664:1997407 | - | 0.71 | - |
| IG1_2016072 | interG_chr1_pos_1080_2016072:2016676 | 0,99 | - | - |
| IG1_2207229 | interG_chr1_pos_1142_2207229:2209972 | - | - | -1.33 |
| IG1_2346535 | interG_chr1_pos_1202_2346535:2354005 | - | - | -0.76 |
| IG1_2363528 | interG_chr1_pos_1207_2363528:2377150 | - | - | 1.23 |
| IG1_2434003 | interG_chr1_pos_1226_2434003:2440929 | - | - | -3.90 |
| IG1_2541097 | interG_chr1_pos_1255_2541097:2541290 | 0,60 | - | 0.76 |
| IG1_2646467 | interG_chr1_pos_1296_2646467:2646584 | - | - | 0.85 |
| IG1_2651825 | interG_chr1_pos_1303_2651825:2652279 | 0,70 | - | - |
| IG1_2784549 | interG_chr1_pos_1366_2784549:2784739 | - | - | -1.45 |
| IG1_286959 | interG_chr1_pos_172_286959:287112 | - | - | 0.70 |
| IG1_2912072 | interG_chr1_pos_1433_2912072:2912451 | - | - | -0.63 |
| IG1_2965090 | interG_chr1_pos_1458_2965090:2965194 | - | -0.83 | - |
| IG1_3051717 | interG_chr1_pos_1497_3051717:3052805 | - | -1.09 | - |
| IG1_3070378 | interG_chr1_pos_1507_3070378:3070549 | - | - | 4.31 |
| IG1_3169576 | interG_chr1_pos_1531_3169576:3169744 | -1,10 | - | -0.65 |
| IG1_322475 | interG_chr1_pos_197_322475:322559 | 1,49 | - | - |
| IG1_32536 | interG_chr1_pos_22_32536:33103 | - | - | -0.91 |
| IG1_3385231 | interG_chr1_pos_1656_3385231:3385326 | - | - | -0.83 |
| IG1_3598341 | interG_chr1_pos_1746_3598341:3598449 | 0,66 | - | - |
| IG1_3647450 | interG_chr1_pos_1774_3647450:3647534 | - | - | -1.11 |
| IG1_3692717 | interG_chr1_pos_1793_3692717:3702285 | - | - | -0.78 |
| IG1_397645 | interG_chr1_pos_257_397645:397799 | - | - | 0.65 |
| IG1_418869 | interG_chr1_pos_267_418869:419121 | 0,75 | - | - |
| IG1_486377 | interG_chr1_pos_301_486377:487637 | - | - | -0.79 |
| IG1_52439 | interG_chr1_pos_35_52439:52522 | - | - | 0.84 |
| IG1_563415 | interG_chr1_pos_336_563415:563974 | - | -0.83 | -1.15 |
| IG1_579967 | interG_chr1_pos_341_579967:580205 | 1,21 | - | - |
| IG1_582769 | interG_chr1_pos_345_582769:583037 | 0,90 | - | - |
| IG1_619308 | interG_chr1_pos_364_619308:619404 | 4,04 | - | 2.40 |
| IG1_631117 | interG_chr1_pos_374_631117:631394 | 3,93 | - | 3.36 |
| IG1_752786 | interG_chr1_pos_418_752786:753250 | -0,87 | - | - |
| IG1_786937 | interG_chr1_pos_442_786937:787316 | -0,59 | - | - |
| IG1_825611 | interG_chr1_pos_472_825611:825836 | 1,46 | - | - |
| IG1_898886 | interG_chr1_pos_500_898886:898984 | - | - | -0.67 |
| IG1_901672 | interG_chr1_pos_504_901672:902168 | - | - | -1.68 |
| IG2_1050346 | interG_chr2_pos_534_1050346:1050566 | - | - | 1.12 |
| IG2_1076340 | interG_chr2_pos_550_1076340:1076405 | 0,61 | - | 0.71 |
| IG2_1099336 | interG_chr2_pos_567_1099336:1099416 | - | - | -0.64 |
| IG2_1199962 | interG_chr2_pos_643_1199962:1204067 | - | - | -1.67 |
| IG2_1239472 | interG_chr2_pos_653_1239472:1245613 | - | 0.68 | 1.27 |
| IG2_1279151 | interG_chr2_pos_671_1279151:1279362 | - | - | -2.26 |
| IG2_1299473 | interG_chr2_pos_679_1299473:1300656 | - | - | 0.76 |
| IG2_1452240 | interG_chr2_pos_749_1452240:1452464 | - | - | 0.62 |
| IG2_1458623 | interG_chr2_pos_754_1458623:1458764 | -0,71 | - | - |
| IG2_1462450 | interG_chr2_pos_757_1462450:1465344 | - | - | -0.73 |
| IG2_1502006 | interG_chr2_pos_772_1502006:1502154 | - | - | -1.86 |
| IG2_1619082 | interG_chr2_pos_821_1619082:1621207 | 0,64 | - | - |
| IG2_1656016 | interG_chr2_pos_843_1656016:1656240 | 1,62 | - | 1.21 |
| IG2_1660910 | interG_chr2_pos_846_1660910:1661218 | - | - | -1.84 |
| IG2_1664765 | interG_chr2_pos_849_1664765:1664977 | - | -1.50 | -1.34 |
| IG2_1669623 | interG_chr2_pos_852_1669623:1670980 | 0,69 | - | - |
| IG2_1713089 | interG_chr2_pos_874_1713089:1714739 | - | - | -0.75 |
| IG2_1722745 | interG_chr2_pos_880_1722745:1728440 | - | - | -0.91 |
| IG2_1750779 | interG_chr2_pos_894_1750779:1751230 | - | -1.03 | -0.86 |
| IG2_1953120 | interG_chr2_pos_965_1953120:1953814 | 2,83 | - | 2.49 |
| IG2_1975919 | interG_chr2_pos_977_1975919:1976205 | - | - | 0.72 |
| IG2_1991391 | interG_chr2_pos_987_1991391:1991519 | - | - | 0.62 |
| IG2_2018202 | interG_chr2_pos_1001_2018202:2019462 | -0,77 | - | - |
| IG2_2044392 | interG_chr2_pos_1018_2044392:2049728 | - | - | -1.96 |
| IG2_2050164 | interG_chr2_pos_1019_2050164:2050639 | - | -0.68 | -1.37 |
| IG2_2087974 | interG_chr2_pos_1034_2087974:2088183 | 0,62 | - | - |
| IG2_2259633 | interG_chr2_pos_1128_2259633:2260078 | -3,91 | 0.97 | -2.21 |
| IG2_2260478 | interG_chr2_pos_1129_2260478:2260877 | - | - | -1.25 |
| IG2_2304176 | interG_chr2_pos_1139_2304176:2304510 | -1,35 | - | -1.33 |
| IG2_2552298 | interG_chr2_pos_1228_2552298:2552384 | - | - | -0.63 |
| IG2_2586922 | interG_chr2_pos_1237_2586922:2587412 | -0,79 | - | - |
| IG2_2590456 | interG_chr2_pos_1239_2590456:2590587 | - | - | 0.62 |
| IG2_2617890 | interG_chr2_pos_1244_2617890:2618092 | -0,72 | - | - |
| IG2_2619918 | interG_chr2_pos_1246_2619918:2631560 | -0,77 | - | - |
| IG2_2701955 | interG_chr2_pos_1284_2701955:2703048 | - | -0.87 | -1.47 |
| IG2_2717368 | interG_chr2_pos_1290_2717368:2718618 | 0,66 | - | - |
| IG2_2731412 | interG_chr2_pos_1296_2731412:2731800 | - | - | -1.57 |
| IG2_2855166 | interG_chr2_pos_1340_2855166:2857103 | - | - | 0.81 |
| IG2_2875859 | interG_chr2_pos_1346_2875859:2876060 | 0,84 | - | - |
| IG2_2907312 | interG_chr2_pos_1356_2907312:2907401 | 1,56 | - | - |
| IG2_2970010 | interG_chr2_pos_1386_2970010:2971563 | - | -0.84 | -0.91 |
| IG2_3040342 | interG_chr2_pos_1416_3040342:3042214 | - | -0.80 | -0.66 |
| IG2_3061820 | interG_chr2_pos_1426_3061820:3063511 | - | - | 0.61 |
| IG2_3096444 | interG_chr2_pos_1435_3096444:3096551 | - | - | -0.98 |
| IG2_3173242 | interG_chr2_pos_1461_3173242:3174483 | 0,66 | - | - |
| IG2_3193737 | interG_chr2_pos_1472_3193737:3194294 | 0,64 | - | - |
| IG2_3195840 | interG_chr2_pos_1473_3195840:3196013 | - | -0.64 | -0.81 |
| IG2_342807 | interG_chr2_pos_157_342807:346518 | - | -1.95 | -2.19 |
| IG2_366910 | interG_chr2_pos_166_366910:367027 | - | -0.84 | -1.01 |
| IG2_369070 | interG_chr2_pos_168_369070:370084 | - | -2.07 | -2.34 |
| IG2_370562 | interG_chr2_pos_169_370562:372148 | - | -0.82 | -0.92 |
| IG2_433416 | interG_chr2_pos_212_433416:433537 | - | - | 0.66 |
| IG2_551370 | interG_chr2_pos_283_551370:553896 | -2,66 | - | -1.27 |
| IG2_643345 | interG_chr2_pos_336_643345:643912 | -1,03 | - | - |
| IG2_764648 | interG_chr2_pos_390_764648:770522 | -2,56 | - | -2.37 |
| IG2_81665 | interG_chr2_pos_38_81665:81872 | - | 0.62 | - |
| IG2_836365 | interG_chr2_pos_424_836365:839355 | -0,82 | - | - |
| IG2_896897 | interG_chr2_pos_456_896897:896992 | -0,71 | - | - |
| IG2_904254 | interG_chr2_pos_460_904254:904769 | -1,14 | - | - |
| IG2_925879 | interG_chr2_pos_474_925879:926112 | - | - | -0.75 |
| IG2_977477 | interG_chr2_pos_504_977477:977615 | - | -0.64 | -0.73 |
| IG3_216945 | interG_chr3_pos_131_216945:241075 | -0,72 | - | - |
| IG3_354019 | interG_chr3_pos_188_354019:357929 | -5,73 | - | -3.11 |
| IG3_493778 | interG_chr3_pos_239_493778:498308 | 1,47 | - | - |
| IG3_536263 | interG_chr3_pos_259_536263:537450 | 0,66 | - | - |
| IG3_574500 | interG_chr3_pos_268_574500:584648 | 2,48 | - | - |
| IG3_584892 | interG_chr3_pos_269_584892:600856 | 0,98 | 0.61 | 0.74 |
| IG3_601100 | interG_chr3_pos_270_601100:601231 | 1,16 | - | 0.75 |
| IG3_607288 | interG_chr3_pos_279_607288:607409 | 2,01 | 0.92 | 1.51 |
| IG3_608028 | interG_chr3_pos_280_608028:608090 | 1,13 | - | 1.07 |
| IG3_608364 | interG_chr3_pos_281_608364:608456 | 1,23 | 0.67 | 0.93 |
| IG3_688383 | interG_chr3_pos_312_688383:688445 | - | - | -0.61 |
| IG3_745205 | interG_chr3_pos_334_745205:745285 | 1,64 | - | 1.11 |
| IG3_811123 | interG_chr3_pos_363_811123:815031 | - | - | -1.24 |
| IG3_81534 | interG_chr3_pos_43_81534:81671 | - | - | -0.91 |
| pBCA005 | conserved hypothetical protein | - | - | 1.11 |
| pBCA046 | putative TraE conjugative transfer protein | - | - | -1.90 |
| pBCA050 | hypothetical protein | - | - | 1.37 |
