# Supplementary material for: Deciphering the Role of RND Efflux Transporters in Burkholderia cenocepacia
Source: PLoS One. 2011 Apr 19;6(4):e18902. doi: 10.1371/journal.pone.0018902 (PMC3079749; doi:10.1371/journal.pone.0018902)
Supplement: Table S2 — Gene Ontology (GO) terms functional enrichment analysis showing the over or under-representation of up-regulated genes of mutant D4 in comparison to B. cenocepacia J2315 whole genome functional annotation. Only GO terms over- or under- represented with an associated p-value <0.05 are shown. (DOC) [file pone.0018902.s009.doc]

**Table S2. Gene Ontology (GO) terms functional enrichment analysis showing the over or under-representation of up-regulated genes of mutant D4 in comparison to *B. cenocepacia* J2315 whole genome functional annotation.**

| GO terms | Name | FDR | FWER | p-Value | Over/Under |
| --- | --- | --- | --- | --- | --- |
| GO:0043064 | flagellum organization | 1.36E-09 | 7.17E-09 | 0.000 | over |
| GO:0044460 | flagellum part | 1.36E-09 | 7.23E-09 | 0.000 | over |
| GO:0003774 | motor activity | 1.36E-09 | 7.23E-09 | 0.000 | over |
| GO:0044461 | bacterial-type flagellum part | 1.36E-09 | 7.23E-09 | 0.000 | over |
| GO:0044463 | cell projection part | 1.36E-09 | 7.23E-09 | 0.000 | over |
| GO:0006935 | chemotaxis | 1.36E-09 | 7.23E-09 | 0.000 | over |
| GO:0007610 | behavior | 1.36E-09 | 7.23E-09 | 0.000 | over |
| GO:0007626 | locomotory behavior | 1.36E-09 | 7.23E-09 | 0.000 | over |
| GO:0042330 | taxis | 1.36E-09 | 7.23E-09 | 0.000 | over |
| GO:0006928 | cellular component movement | 1.36E-09 | 7.27E-09 | 0.000 | over |
| GO:0048870 | cell motility | 1.36E-09 | 7.31E-09 | 0.000 | over |
| GO:0001539 | ciliary or flagellar motility | 1.36E-09 | 7.31E-09 | 0.000 | over |
| GO:0042221 | response to chemical stimulus | 1.36E-09 | 7.31E-09 | 0.000 | over |
| GO:0019861 | flagellum | 1.36E-09 | 7.31E-09 | 0.000 | over |
| GO:0043229 | intracellular organelle | 1.36E-09 | 7.33E-09 | 0.000 | over |
| GO:0043226 | organelle | 1.36E-09 | 7.33E-09 | 0.000 | over |
| GO:0042995 | cell projection | 1.36E-09 | 7.33E-09 | 0.000 | over |
| GO:0030030 | cell projection organization | 1.36E-09 | 7.40E-09 | 0.000 | over |
| GO:0040011 | locomotion | 1.36E-09 | 7.40E-09 | 0.000 | over |
| GO:0009605 | response to external stimulus | 1.36E-09 | 7.40E-09 | 0.000 | over |
| GO:0009296 | flagellum assembly | 1.36E-09 | 7.61E-09 | 0.000 | over |
| GO:0009288 | bacterial-type flagellum | 1.36E-09 | 7.61E-09 | 0.000 | over |
| GO:0030031 | cell projection assembly | 1.36E-09 | 7.84E-09 | 0.000 | over |
| GO:0007165 | signal transduction | 1.75E-08 | 1.05E-07 | 0.000 | over |
| GO:0050896 | response to stimulus | 9.98E-08 | 6.24E-07 | 0.000 | over |
| GO:0004871 | signal transducer activity | 3.79E-06 | 2.56E-05 | 0.000 | over |
| GO:0060089 | molecular transducer activity | 3.79E-06 | 2.56E-05 | 0.000 | over |
| GO:0008152 | metabolic process | 0 | 0 | 0.000 | under |
| GO:0017111 | nucleoside-triphosphatase activity | 5.32E-05 | 3.99E-04 | 0.000 | over |
| GO:0005488 | binding | 0 | 0 | 0.000 | under |
| GO:0022607 | cellular component assembly | 1.10E-04 | 9.47E-04 | 0.000 | over |
| GO:0016462 | pyrophosphatase activity | 1.10E-04 | 9.66E-04 | 0.000 | over |
| GO:0016818 | hydrolase activity, acting on acid anhydrides, in phosphorus-containing anhydrides | 1.10E-04 | 9.88E-04 | 0.000 | over |
| GO:0016817 | hydrolase activity, acting on acid anhydrides | 1.10E-04 | 9.88E-04 | 0.000 | over |
| GO:0005198 | structural molecule activity | 1.10E-04 | 0.001015 | 0.000 | over |
| GO:0004888 | transmembrane receptor activity | 3.23E-04 | 0.003067 | 0.000 | over |
| GO:0016043 | cellular component organization | 4.55E-04 | 0.00443 | 0.000 | over |
| GO:0004872 | receptor activity | 0.001022 | 0.010168 | 0.000 | over |
| GO:0044464 | cell part | 0.002441 | 0.024711 | 0.000 | over |
| GO:0044237 | cellular metabolic process | 0 | 0 | 0.001 | under |
| GO:0016787 | hydrolase activity | 0.011352 | 0.122387 | 0.001 | over |
| GO:0030243 | cellulose metabolic process | 0.014503 | 0.165814 | 0.001 | over |
| GO:0015930 | glutamate synthase activity | 0.014503 | 0.165814 | 0.001 | over |
| GO:0045181 | glutamate synthase activity, NADH or NADPH as acceptor | 0.014503 | 0.165814 | 0.001 | over |
| GO:0030244 | cellulose biosynthetic process | 0.014503 | 0.165814 | 0.001 | over |
| GO:0032991 | macromolecular complex | 0 | 0 | 0.001 | under |
| GO:0005667 | transcription factor complex | 0 | 0 | 0.001 | under |
| GO:0003700 | transcription factor activity | 0 | 0 | 0.001 | under |
| GO:0003676 | nucleic acid binding | 0 | 0 | 0.002 | under |
| GO:0016491 | oxidoreductase activity | 0 | 0 | 0.002 | under |
| GO:0009250 | glucan biosynthetic process | 0.024463 | 0.272441 | 0.002 | over |
| GO:0006011 | UDP-glucose metabolic process | 0.024463 | 0.272441 | 0.002 | over |
| GO:0044422 | organelle part | 0 | 0 | 0.003 | under |
| GO:0044446 | intracellular organelle part | 0 | 0 | 0.003 | under |
| GO:0043234 | protein complex | 0 | 0 | 0.003 | under |
| GO:0016639 | oxidoreductase activity, acting on the CH-NH2 group of donors, NAD or NADP as acceptor | 0.038261 | 0.397743 | 0.003 | over |
| GO:0015031 | protein transport | 0.046829 | 0.474861 | 0.005 | over |
| GO:0045184 | establishment of protein localization | 0.046829 | 0.474861 | 0.005 | over |
| GO:0006537 | glutamate biosynthetic process | 0.048788 | 0.530685 | 0.005 | over |
| GO:0031328 | positive regulation of cellular biosynthetic process | 0.048788 | 0.530685 | 0.005 | over |
| GO:0009891 | positive regulation of biosynthetic process | 0.048788 | 0.530685 | 0.005 | over |
| GO:0010628 | positive regulation of gene expression | 0.048788 | 0.530685 | 0.005 | over |
| GO:0010557 | positive regulation of macromolecule biosynthetic process | 0.048788 | 0.530685 | 0.005 | over |
| GO:0045941 | positive regulation of transcription | 0.048788 | 0.530685 | 0.005 | over |
| GO:0016563 | transcription activator activity | 0.048788 | 0.530685 | 0.005 | over |
| GO:0044428 | nuclear part | 0 | 0 | 0.006 | under |
| GO:0044451 | nucleoplasm part | 0 | 0 | 0.006 | under |
| GO:0003677 | DNA binding | 0 | 0 | 0.009 | under |
| GO:0009058 | biosynthetic process | 0 | 0 | 0.009 | under |
| GO:0046872 | metal ion binding | 0 | 0 | 0.011 | under |
| GO:0055114 | oxidation reduction | 0 | 0 | 0.015 | under |
| GO:0032787 | monocarboxylic acid metabolic process | 0 | 0 | 0.021 | under |
| GO:0044249 | cellular biosynthetic process | 0 | 0 | 0.022 | under |
| GO:0046483 | heterocycle metabolic process | 0 | 0 | 0.037 | under |
| GO:0044238 | primary metabolic process | 0 | 0 | 0.039 | under |
| GO:0005215 | transporter activity | 0 | 0 | 0.039 | under |
| GO:0046914 | transition metal ion binding | 0 | 0 | 0.039 | under |
| GO:0043167 | ion binding | 0 | 0 | 0.040 | under |
| GO:0043169 | cation binding | 0 | 0 | 0.040 | under |
| GO:0019752 | carboxylic acid metabolic process | 0 | 0 | 0.050 | under |
| GO:0006082 | organic acid metabolic process | 0 | 0 | 0.050 | under |
