# Supplementary material for: Deciphering the Role of RND Efflux Transporters in Burkholderia cenocepacia
Source: PLoS One. 2011 Apr 19;6(4):e18902. doi: 10.1371/journal.pone.0018902 (PMC3079749; doi:10.1371/journal.pone.0018902)
Supplement: Table S3 — Gene Ontology (GO) terms functional enrichment analysis showing the over or under-representation of down-regulated genes of mutant D4 in comparison to B. cenocepacia J2315 whole genome functional annotation. (DOC) [file pone.0018902.s010.doc]

**Table S3. Gene Ontology (GO) terms functional enrichment analysis showing the over or under-representation of down-regulated genes of mutant D4 in comparison to *B. cenocepacia* J2315 whole genome functional annotation.**

| GO terms | Name | FDR | FWER | p-Value | Over/Under |
| --- | --- | --- | --- | --- | --- |
| GO:0000166 | nucleotide binding | 0 | 0 | 0.054719 | under |
| GO:0009058 | biosynthetic process | 0 | 0 | 0.063258 | under |
| GO:0048037 | cofactor binding | 0 | 0 | 0.08831 | under |
| GO:0017076 | purine nucleotide binding | 0 | 0 | 0.104298 | under |
| GO:0022857 | transmembrane transporter activity | 0 | 0 | 0.105307 | under |
| GO:0042180 | cellular ketone metabolic process | 0 | 0 | 0.119366 | under |
| GO:0001882 | nucleoside binding | 0 | 0 | 0.123531 | under |
| GO:0030554 | adenyl nucleotide binding | 0 | 0 | 0.125132 | under |
| GO:0001883 | purine nucleoside binding | 0 | 0 | 0.125132 | under |
| GO:0022891 | substrate-specific transmembrane transporter activity | 0 | 0 | 0.135787 | under |
| GO:0044271 | cellular nitrogen compound biosynthetic process | 0 | 0 | 0.136865 | under |
| GO:0043436 | oxoacid metabolic process | 0 | 0 | 0.140027 | under |
| GO:0019752 | carboxylic acid metabolic process | 0 | 0 | 0.140027 | under |
| GO:0006082 | organic acid metabolic process | 0 | 0 | 0.140027 | under |
| GO:0016787 | hydrolase activity | 0 | 0 | 0.140832 | under |
| GO:0044249 | cellular biosynthetic process | 0 | 0 | 0.143208 | under |
| GO:0051186 | cofactor metabolic process | 0 | 0 | 0.148113 | under |
| GO:0044281 | small molecule metabolic process | 0 | 0 | 0.156537 | under |
| GO:0005886 | plasma membrane | 0 | 0 | 0.166677 | under |
| GO:0044238 | primary metabolic process | 0 | 0 | 0.174431 | under |
| GO:0032553 | ribonucleotide binding | 0 | 0 | 0.180184 | under |
| GO:0032555 | purine ribonucleotide binding | 0 | 0 | 0.180184 | under |
| GO:0003824 | catalytic activity | 0 | 0 | 0.186886 | under |
| GO:0044283 | small molecule biosynthetic process | 0 | 0 | 0.186919 | under |
| GO:0050662 | coenzyme binding | 0 | 0 | 0.198027 | under |
| GO:0005737 | cytoplasm | 0 | 0 | 0.205909 | under |
| GO:0015075 | ion transmembrane transporter activity | 0 | 0 | 0.207522 | under |
| GO:0022804 | active transmembrane transporter activity | 0 | 0 | 0.209146 | under |
| GO:0023060 | signal transmission | 0 | 0 | 0.210783 | under |
| GO:0023046 | signaling process | 0 | 0 | 0.210783 | under |
| GO:0005524 | ATP binding | 0 | 0 | 0.213467 | under |
| GO:0032559 | adenyl ribonucleotide binding | 0 | 0 | 0.213467 | under |
| GO:0006811 | ion transport | 0 | 0 | 0.214094 | under |
| GO:0060089 | molecular transducer activity | 0 | 0 | 0.224332 | under |
| GO:0004871 | signal transducer activity | 0 | 0 | 0.224332 | under |
| GO:0044444 | cytoplasmic part | 0 | 0 | 0.229627 | under |
| GO:0006732 | coenzyme metabolic process | 0 | 0 | 0.242456 | under |
| GO:0016887 | ATPase activity | 0 | 0 | 0.25204 | under |
| GO:0044424 | intracellular part | 0 | 0 | 0.255717 | under |
| GO:0044262 | cellular carbohydrate metabolic process | 0 | 0 | 0.274433 | under |
| GO:0016614 | oxidoreductase activity, acting on CH-OH group of donors | 0 | 0 | 0.276561 | under |
| GO:0042967 | acyl-carrier-protein biosynthetic process | 0 | 0 | 0.294173 | under |
| GO:0005488 | binding | 0 | 0 | 0.294439 | under |
| GO:0006066 | alcohol metabolic process | 0 | 0 | 0.315283 | under |
| GO:0016746 | transferase activity, transferring acyl groups | 0 | 0 | 0.320169 | under |
| GO:0016616 | oxidoreductase activity, acting on the CH-OH group of donors, NAD or NADP as acceptor | 0 | 0 | 0.32264 | under |
| GO:0016788 | hydrolase activity, acting on ester bonds | 0 | 0 | 0.325129 | under |
| GO:0016747 | transferase activity, transferring acyl groups other than amino-acyl groups | 0 | 0 | 0.327636 | under |
| GO:0032787 | monocarboxylic acid metabolic process | 0 | 0 | 0.331949 | under |
| GO:0009059 | macromolecule biosynthetic process | 0 | 0 | 0.331949 | under |
| GO:0008324 | cation transmembrane transporter activity | 0 | 0 | 0.332707 | under |
| GO:0008415 | acyltransferase activity | 0 | 0 | 0.335271 | under |
| GO:0006519 | cellular amino acid and derivative metabolic process | 0 | 0 | 0.344015 | under |
| GO:0051188 | cofactor biosynthetic process | 0 | 0 | 0.34838 | under |
| GO:0016874 | ligase activity | 0 | 0 | 0.35106 | under |
| GO:0000160 | two-component signal transduction system (phosphorelay) | 0 | 0 | 0.353761 | under |
| GO:0009309 | amine biosynthetic process | 0 | 0 | 0.356481 | under |
| GO:0042623 | ATPase activity, coupled | 0 | 0 | 0.364765 | under |
| GO:0016817 | hydrolase activity, acting on acid anhydrides | 0 | 0 | 0.371815 | under |
| GO:0016818 | hydrolase activity, acting on acid anhydrides, in phosphorus-containing anhydrides | 0 | 0 | 0.371815 | under |
| GO:0050794 | regulation of cellular process | 0 | 0 | 0.372713 | under |
| GO:0016462 | pyrophosphatase activity | 0 | 0 | 0.375788 | under |
| GO:0006812 | cation transport | 0 | 0 | 0.381893 | under |
| GO:0008652 | cellular amino acid biosynthetic process | 0 | 0 | 0.384823 | under |
| GO:0018130 | heterocycle biosynthetic process | 0 | 0 | 0.393742 | under |
| GO:0008152 | metabolic process | 0 | 0 | 0.3978 | under |
| GO:0017111 | nucleoside-triphosphatase activity | 0 | 0 | 0.39819 | under |
| GO:0005996 | monosaccharide metabolic process | 0 | 0 | 0.405945 | under |
| GO:0044106 | cellular amine metabolic process | 0 | 0 | 0.410216 | under |
| GO:0016051 | carbohydrate biosynthetic process | 0 | 0 | 0.412183 | under |
| GO:0006520 | cellular amino acid metabolic process | 0 | 0 | 0.42076 | under |
| GO:0008233 | peptidase activity | 0 | 0 | 0.421714 | under |
| GO:0032991 | macromolecular complex | 0 | 0 | 0.425124 | under |
| GO:0044462 | external encapsulating structure part | 0 | 0 | 0.428186 | under |
| GO:0005975 | carbohydrate metabolic process | 0 | 0 | 0.44129 | under |
| GO:0046394 | carboxylic acid biosynthetic process | 0 | 0 | 0.445766 | under |
| GO:0016053 | organic acid biosynthetic process | 0 | 0 | 0.445766 | under |
| GO:0006753 | nucleoside phosphate metabolic process | 0 | 0 | 0.448184 | under |
| GO:0009117 | nucleotide metabolic process | 0 | 0 | 0.448184 | under |
| GO:0044464 | cell part | 0 | 0 | 0.448977 | under |
| GO:0015405 | P-P-bond-hydrolysis-driven transmembrane transporter activity | 0 | 0 | 0.451603 | under |
| GO:0015399 | primary active transmembrane transporter activity | 0 | 0 | 0.451603 | under |
| GO:0019842 | vitamin binding | 0 | 0 | 0.451603 | under |
| GO:0016820 | hydrolase activity, acting on acid anhydrides, catalyzing transmembrane movement of substances | 0 | 0 | 0.451603 | under |
| GO:0043492 | ATPase activity, coupled to movement of substances | 0 | 0 | 0.476256 | under |
| GO:0042626 | ATPase activity, coupled to transmembrane movement of substances | 0 | 0 | 0.476256 | under |
| GO:0009108 | coenzyme biosynthetic process | 0 | 0 | 0.479882 | under |
| GO:0015291 | secondary active transmembrane transporter activity | 0 | 0 | 0.479882 | under |
| GO:0019538 | protein metabolic process | 0 | 0 | 0.482635 | under |
| GO:0070011 | peptidase activity, acting on L-amino acid peptides | 0 | 0 | 0.483535 | under |
| GO:0019318 | hexose metabolic process | 0 | 0 | 0.483535 | under |
| GO:0016491 | oxidoreductase activity | 0 | 0 | 0.492625 | under |
| GO:0016020 | membrane | 0 | 0 | 0.492625 | under |
| GO:0005976 | polysaccharide metabolic process | 0 | 0 | 0.494656 | under |
| GO:0055114 | oxidation reduction | 0 | 0 | 0.496884 | under |
