# Supplementary material for: Deciphering the Role of RND Efflux Transporters in Burkholderia cenocepacia
Source: PLoS One. 2011 Apr 19;6(4):e18902. doi: 10.1371/journal.pone.0018902 (PMC3079749; doi:10.1371/journal.pone.0018902)
Supplement: Table S4 — Gene Ontology (GO) terms functional enrichment analysis showing the over or under-representation of up-regulated genes of mutant D9 in comparison to B. cenocepacia J2315 whole genome functional annotation. Only GO terms over- or under- represented with an associated p-value <0.05 are shown. (DOC) [file pone.0018902.s011.doc]

**Table S4. Gene Ontology (GO) terms functional enrichment analysis showing the over or under-representation of up-regulated genes of mutant D9 in comparison to *B. cenocepacia* J2315 whole genome functional annotation.**

| GO terms | Name | FDR | FWER | p-Value | Over/Under |
| --- | --- | --- | --- | --- | --- |
| GO:0009432 | SOS response | 0.1176 | 0.100141 | 8.94E-04 | over |
| GO:0031668 | cellular response to extracellular stimulus | 0.1176 | 0.136718 | 0.001358 | over |
| GO:0071496 | cellular response to external stimulus | 0.1176 | 0.136718 | 0.001358 | over |
| GO:0007154 | cell communication | 0.1176 | 0.136718 | 0.001358 | over |
| GO:0009991 | response to extracellular stimulus | 0.1176 | 0.136718 | 0.001358 | over |
| GO:0019357 | nicotinate nucleotide biosynthetic process | 0.87248 | 0.782963 | 0.005162 | over |
| GO:0004515 | nicotinate-nucleotide adenylyltransferase activity | 0.87248 | 0.782963 | 0.005162 | over |
| GO:0004000 | adenosine deaminase activity | 0.954085 | 0.983185 | 0.015411 | over |
| GO:0005737 | cytoplasm | 0.954085 | 0.986936 | 0.02001 | over |
| GO:0006281 | DNA repair | 0.954085 | 0.987599 | 0.020182 | over |
| GO:0006974 | response to DNA damage stimulus | 0.954085 | 0.987599 | 0.020182 | over |
| GO:0003697 | single-stranded DNA binding | 0.954085 | 0.993232 | 0.020497 | over |
| GO:0009605 | response to external stimulus | 0.954085 | 0.99351 | 0.021078 | over |
| GO:0006289 | nucleotide-excision repair | 0.954085 | 0.997229 | 0.025559 | over |
| GO:0009380 | excinuclease repair complex | 0.954085 | 0.997229 | 0.025559 | over |
| GO:0004190 | aspartic-type endopeptidase activity | 0.954085 | 0.997229 | 0.025559 | over |
| GO:0070001 | aspartic-type peptidase activity | 0.954085 | 0.997229 | 0.025559 | over |
| GO:0003684 | damaged DNA binding | 0.954085 | 0.997229 | 0.025559 | over |
| GO:0009381 | excinuclease ABC activity | 0.954085 | 0.997229 | 0.025559 | over |
| GO:0033554 | cellular response to stress | 0.954085 | 0.997295 | 0.025801 | over |
| GO:0006259 | DNA metabolic process | 0.954085 | 0.997337 | 0.026302 | over |
| GO:0051716 | cellular response to stimulus | 0.954085 | 0.997478 | 0.026793 | over |
| GO:0003677 | DNA binding | 0.954085 | 0.997695 | 0.029614 | over |
| GO:0016814 | hydrolase activity, acting on carbon-nitrogen (but not peptide) bonds, in cyclic amidines | 0.954085 | 0.998941 | 0.030595 | over |
| GO:0004518 | nuclease activity | 0.954085 | 0.999006 | 0.031982 | over |
| GO:0016787 | hydrolase activity | 0.954085 | 0.999181 | 0.035378 | over |
| GO:0043566 | structure-specific DNA binding | 0.954085 | 0.999541 | 0.035607 | over |
| GO:0009326 | formate dehydrogenase complex | 0.954085 | 0.999789 | 0.040594 | over |
| GO:0008863 | formate dehydrogenase activity | 0.954085 | 0.999789 | 0.040594 | over |
| GO:0009435 | NAD biosynthetic process | 0.954085 | 0.999789 | 0.040594 | over |
| GO:0008408 | 3'-5' exonuclease activity | 0.954085 | 0.999789 | 0.040594 | over |
| GO:0019674 | NAD metabolic process | 0.954085 | 0.999789 | 0.040594 | over |
| GO:0019359 | nicotinamide nucleotide biosynthetic process | 0.954085 | 0.999789 | 0.040594 | over |
| GO:0015942 | formate metabolic process | 0.954085 | 0.999789 | 0.040594 | over |
| GO:0019363 | pyridine nucleotide biosynthetic process | 0.954085 | 0.999789 | 0.040594 | over |
| GO:0016779 | nucleotidyltransferase activity | 0.954085 | 0.999818 | 0.043437 | over |
| GO:0008094 | DNA-dependent ATPase activity | 0.974996 | 0.999908 | 0.045556 | over |
| GO:0004520 | endodeoxyribonuclease activity | 0.974996 | 0.999908 | 0.045556 | over |
