# Supplementary material for: Deciphering the Role of RND Efflux Transporters in Burkholderia cenocepacia
Source: PLoS One. 2011 Apr 19;6(4):e18902. doi: 10.1371/journal.pone.0018902 (PMC3079749; doi:10.1371/journal.pone.0018902)
Supplement: Table S5 — Gene Ontology (GO) terms functional enrichment analysis showing the over or under-representation of down-regulated genes of mutant D9 in comparison to B. cenocepacia J2315 whole genome functional annotation. Only GO terms over- or under- represented with an associated p-value <0.05 are shown. (DOC) [file pone.0018902.s012.doc]

**Table S5.** **Gene Ontology (GO) terms functional enrichment analysis showing the over or under-representation of down-regulated genes of mutant D9 in comparison to *B. cenocepacia* J2315 whole genome functional annotation.**

| GO terms | Name | FDR | FWER | p-Value | Over/Under |
| --- | --- | --- | --- | --- | --- |
| GO:0040011 | locomotion | 2.15E-05 | 5.37E-06 | 6.13E-08 | over |
| GO:0044463 | cell projection part | 2.59E-05 | 2.59E-05 | 3.34E-07 | over |
| GO:0044461 | bacterial-type flagellum part | 2.59E-05 | 2.59E-05 | 3.34E-07 | over |
| GO:0044460 | flagellum part | 2.59E-05 | 2.59E-05 | 3.34E-07 | over |
| GO:0019861 | flagellum | 4.18E-05 | 5.22E-05 | 6.15E-07 | over |
| GO:0048870 | cell motility | 7.55E-05 | 1.32E-04 | 1.55E-06 | over |
| GO:0001539 | ciliary or flagellar motility | 7.55E-05 | 1.32E-04 | 1.55E-06 | over |
| GO:0042995 | cell projection | 8.20E-05 | 1.64E-04 | 1.87E-06 | over |
| GO:0006928 | cellular component movement | 1.45E-04 | 3.25E-04 | 2.85E-06 | over |
| GO:0003774 | motor activity | 6.22E-04 | 0.001554 | 1.56E-05 | over |
| GO:0004022 | alcohol dehydrogenase (NAD) activity | 0.001615 | 0.004433 | 3.84E-05 | over |
| GO:0050896 | response to stimulus | 0.001921 | 0.005746 | 5.52E-05 | over |
| GO:0043229 | intracellular organelle | 0.004134 | 0.014365 | 1.50E-04 | over |
| GO:0043226 | organelle | 0.004134 | 0.014365 | 1.50E-04 | over |
| GO:0043064 | flagellum organization | 0.010587 | 0.038926 | 2.22E-04 | over |
| GO:0030030 | cell projection organization | 0.01189 | 0.046446 | 3.02E-04 | over |
| GO:0030243 | cellulose metabolic process | 0.018044 | 0.077991 | 4.77E-04 | over |
| GO:0030244 | cellulose biosynthetic process | 0.018044 | 0.077991 | 4.77E-04 | over |
| GO:0008106 | alcohol dehydrogenase (NADP+) activity | 0.028626 | 0.139549 | 9.46E-04 | over |
| GO:0009250 | glucan biosynthetic process | 0.028626 | 0.139549 | 9.46E-04 | over |
| GO:0006011 | UDP-glucose metabolic process | 0.028626 | 0.139549 | 9.46E-04 | over |
| GO:0006950 | response to stress | 0.035035 | 0.175277 | 0.001469 | over |
| GO:0034637 | cellular carbohydrate biosynthetic process | 0.046449 | 0.24399 | 0.002186 | over |
| GO:0006006 | glucose metabolic process | 0.046449 | 0.256051 | 0.002322 | over |
| GO:0009891 | positive regulation of biosynthetic process | 0.046449 | 0.294204 | 0.002327 | over |
| GO:0010628 | positive regulation of gene expression | 0.046449 | 0.294204 | 0.002327 | over |
| GO:0045941 | positive regulation of transcription | 0.046449 | 0.294204 | 0.002327 | over |
| GO:0031328 | positive regulation of cellular biosynthetic process | 0.046449 | 0.294204 | 0.002327 | over |
| GO:0010557 | positive regulation of macromolecule biosynthetic process | 0.046449 | 0.294204 | 0.002327 | over |
| GO:0016563 | transcription activator activity | 0.046449 | 0.294204 | 0.002327 | over |
| GO:0048522 | positive regulation of cellular process | 0.04788 | 0.365529 | 0.003231 | over |
| GO:0048518 | positive regulation of biological process | 0.04788 | 0.365529 | 0.003231 | over |
| GO:0004033 | aldo-keto reductase activity | 0.04788 | 0.365529 | 0.003231 | over |
| GO:0009893 | positive regulation of metabolic process | 0.04788 | 0.365529 | 0.003231 | over |
| GO:0051173 | positive regulation of nitrogen compound metabolic process | 0.04788 | 0.365529 | 0.003231 | over |
| GO:0031325 | positive regulation of cellular metabolic process | 0.04788 | 0.365529 | 0.003231 | over |
| GO:0010604 | positive regulation of macromolecule metabolic process | 0.04788 | 0.365529 | 0.003231 | over |
| GO:0045935 | positive regulation of nucleobase, nucleoside, nucleotide and nucleic acid metabolic process | 0.04788 | 0.365529 | 0.003231 | over |
| GO:0008270 | zinc ion binding | 0.054931 | 0.417515 | 0.004253 | over |
| GO:0009288 | bacterial-type flagellum | 0.054931 | 0.446055 | 0.004272 | over |
| GO:0009296 | flagellum assembly | 0.054931 | 0.446055 | 0.004272 | over |
| GO:0018883 | caprolactam metabolic process | 0.054931 | 0.446055 | 0.004272 | over |
| GO:0019384 | caprolactam catabolic process | 0.054931 | 0.446055 | 0.004272 | over |
| GO:0030031 | cell projection assembly | 0.067355 | 0.523454 | 0.005448 | over |
| GO:0017111 | nucleoside-triphosphatase activity | 0.073108 | 0.560806 | 0.00642 | over |
| GO:0043605 | cellular amide catabolic process | 0.077515 | 0.5901 | 0.006755 | over |
| GO:0019318 | hexose metabolic process | 0.079954 | 0.611054 | 0.007446 | over |
| GO:0007610 | behavior | 0.079954 | 0.660922 | 0.008204 | over |
| GO:0006935 | chemotaxis | 0.079954 | 0.660922 | 0.008204 | over |
| GO:0007626 | locomotory behavior | 0.079954 | 0.660922 | 0.008204 | over |
| GO:0006164 | purine nucleotide biosynthetic process | 0.079954 | 0.660922 | 0.008204 | over |
| GO:0042330 | taxis | 0.079954 | 0.660922 | 0.008204 | over |
| GO:0016462 | pyrophosphatase activity | 0.079954 | 0.661962 | 0.008299 | over |
| GO:0016817 | hydrolase activity, acting on acid anhydrides | 0.079954 | 0.667133 | 0.008681 | over |
| GO:0016818 | hydrolase activity, acting on acid anhydrides, in phosphorus-containing anhydrides | 0.079954 | 0.667133 | 0.008681 | over |
| GO:0006163 | purine nucleotide metabolic process | 0.080764 | 0.677413 | 0.008917 | over |
| GO:0009225 | nucleotide-sugar metabolic process | 0.085609 | 0.704985 | 0.009747 | over |
| GO:0006753 | nucleoside phosphate metabolic process | 0.087291 | 0.725197 | 0.01116 | over |
| GO:0009117 | nucleotide metabolic process | 0.087291 | 0.725197 | 0.01116 | over |
| GO:0006096 | glycolysis | 0.087291 | 0.730265 | 0.011272 | over |
| GO:0045493 | xylan catabolic process | 0.187005 | 0.985375 | 0.012783 | over |
| GO:0004594 | pantothenate kinase activity | 0.187005 | 0.985375 | 0.012783 | over |
| GO:0005887 | integral to plasma membrane | 0.187005 | 0.985375 | 0.012783 | over |
| GO:0040012 | regulation of locomotion | 0.187005 | 0.985375 | 0.012783 | over |
| GO:0031226 | intrinsic to plasma membrane | 0.187005 | 0.985375 | 0.012783 | over |
| GO:0044087 | regulation of cellular component biogenesis | 0.187005 | 0.985375 | 0.012783 | over |
| GO:0043758 | acetate-CoA ligase (ADP-forming) activity | 0.187005 | 0.985375 | 0.012783 | over |
| GO:0005945 | 6-phosphofructokinase complex | 0.187005 | 0.985375 | 0.012783 | over |
| GO:0050795 | regulation of behavior | 0.187005 | 0.985375 | 0.012783 | over |
| GO:0030257 | type III protein secretion system complex | 0.187005 | 0.985375 | 0.012783 | over |
| GO:0032101 | regulation of response to external stimulus | 0.187005 | 0.985375 | 0.012783 | over |
| GO:0010383 | cell wall polysaccharide metabolic process | 0.187005 | 0.985375 | 0.012783 | over |
| GO:0008443 | phosphofructokinase activity | 0.187005 | 0.985375 | 0.012783 | over |
| GO:0030092 | regulation of flagellum assembly | 0.187005 | 0.985375 | 0.012783 | over |
| GO:0009427 | bacterial-type flagellum basal body, distal rod, L ring | 0.187005 | 0.985375 | 0.012783 | over |
| GO:0031344 | regulation of cell projection organization | 0.187005 | 0.985375 | 0.012783 | over |
| GO:0030694 | bacterial-type flagellum basal body, rod | 0.187005 | 0.985375 | 0.012783 | over |
| GO:0009428 | bacterial-type flagellum basal body, distal rod, P ring | 0.187005 | 0.985375 | 0.012783 | over |
| GO:0060491 | regulation of cell projection assembly | 0.187005 | 0.985375 | 0.012783 | over |
| GO:0050568 | protein-glutamine glutaminase activity | 0.187005 | 0.985375 | 0.012783 | over |
| GO:0050182 | phosphate butyryltransferase activity | 0.187005 | 0.985375 | 0.012783 | over |
| GO:0050920 | regulation of chemotaxis | 0.187005 | 0.985375 | 0.012783 | over |
| GO:0008662 | 1-phosphofructokinase activity | 0.187005 | 0.985375 | 0.012783 | over |
| GO:0003872 | 6-phosphofructokinase activity | 0.187005 | 0.985375 | 0.012783 | over |
| GO:0010410 | hemicellulose metabolic process | 0.187005 | 0.985375 | 0.012783 | over |
| GO:0045491 | xylan metabolic process | 0.187005 | 0.985375 | 0.012783 | over |
| GO:0016043 | cellular component organization | 0.187005 | 0.986901 | 0.013499 | over |
| GO:0022607 | cellular component assembly | 0.187005 | 0.987954 | 0.014921 | over |
| GO:0009308 | amine metabolic process | 0 | 0 | 0.01575 | under |
| GO:0005198 | structural molecule activity | 0.187005 | 0.989546 | 0.015926 | over |
| GO:0016052 | carbohydrate catabolic process | 0.187005 | 0.989714 | 0.016168 | over |
| GO:0055086 | nucleobase, nucleoside and nucleotide metabolic process | 0.187005 | 0.989777 | 0.016241 | over |
| GO:0016051 | carbohydrate biosynthetic process | 0.187005 | 0.989888 | 0.016533 | over |
| GO:0019319 | hexose biosynthetic process | 0.187005 | 0.990508 | 0.016969 | over |
| GO:0006094 | gluconeogenesis | 0.187005 | 0.990508 | 0.016969 | over |
| GO:0006754 | ATP biosynthetic process | 0.187005 | 0.991104 | 0.017153 | over |
| GO:0046034 | ATP metabolic process | 0.187005 | 0.991104 | 0.017153 | over |
| GO:0005996 | monosaccharide metabolic process | 0.187005 | 0.991246 | 0.017667 | over |
| GO:0009605 | response to external stimulus | 0.187005 | 0.991505 | 0.018049 | over |
| GO:0090304 | nucleic acid metabolic process | 0 | 0 | 0.018649 | under |
| GO:0006091 | generation of precursor metabolites and energy | 0.187005 | 0.991736 | 0.01915 | over |
| GO:0046364 | monosaccharide biosynthetic process | 0.187005 | 0.991976 | 0.019166 | over |
| GO:0009142 | nucleoside triphosphate biosynthetic process | 0.187005 | 0.992504 | 0.019282 | over |
| GO:0009145 | purine nucleoside triphosphate biosynthetic process | 0.187005 | 0.992504 | 0.019282 | over |
| GO:0009206 | purine ribonucleoside triphosphate biosynthetic process | 0.187005 | 0.992504 | 0.019282 | over |
| GO:0009201 | ribonucleoside triphosphate biosynthetic process | 0.187005 | 0.992504 | 0.019282 | over |
| GO:0046165 | alcohol biosynthetic process | 0.187005 | 0.992974 | 0.020322 | over |
| GO:0003676 | nucleic acid binding | 0 | 0 | 0.020943 | under |
| GO:0044270 | cellular nitrogen compound catabolic process | 0.187005 | 0.993926 | 0.021517 | over |
| GO:0009199 | ribonucleoside triphosphate metabolic process | 0.187005 | 0.993926 | 0.021517 | over |
| GO:0009205 | purine ribonucleoside triphosphate metabolic process | 0.187005 | 0.993926 | 0.021517 | over |
| GO:0009144 | purine nucleoside triphosphate metabolic process | 0.187005 | 0.993926 | 0.021517 | over |
| GO:0009141 | nucleoside triphosphate metabolic process | 0.191822 | 0.994926 | 0.023854 | over |
| GO:0016566 | specific transcriptional repressor activity | 0.264995 | 0.999676 | 0.025405 | over |
| GO:0009975 | cyclase activity | 0.264995 | 0.999676 | 0.025405 | over |
| GO:0016759 | cellulose synthase activity | 0.264995 | 0.999676 | 0.025405 | over |
| GO:0048583 | regulation of response to stimulus | 0.264995 | 0.999676 | 0.025405 | over |
| GO:0046058 | cAMP metabolic process | 0.264995 | 0.999676 | 0.025405 | over |
| GO:0006171 | cAMP biosynthetic process | 0.264995 | 0.999676 | 0.025405 | over |
| GO:0018454 | acetoacetyl-CoA reductase activity | 0.264995 | 0.999676 | 0.025405 | over |
| GO:0016760 | cellulose synthase (UDP-forming) activity | 0.264995 | 0.999676 | 0.025405 | over |
| GO:0008568 | microtubule-severing ATPase activity | 0.264995 | 0.999676 | 0.025405 | over |
| GO:0030254 | protein secretion by the type III secretion system | 0.264995 | 0.999676 | 0.025405 | over |
| GO:0004016 | adenylate cyclase activity | 0.264995 | 0.999676 | 0.025405 | over |
| GO:0044106 | cellular amine metabolic process | 0 | 0 | 0.027541 | under |
| GO:0009165 | nucleotide biosynthetic process | 0.27629 | 0.999785 | 0.029468 | over |
| GO:0006520 | cellular amino acid metabolic process | 0 | 0 | 0.029588 | under |
| GO:0033692 | cellular polysaccharide biosynthetic process | 0.279226 | 0.999821 | 0.03145 | over |
| GO:0042221 | response to chemical stimulus | 0.279226 | 0.999829 | 0.03217 | over |
| GO:0046700 | heterocycle catabolic process | 0.284329 | 0.999865 | 0.034168 | over |
| GO:0009424 | bacterial-type flagellum hook | 0.312094 | 0.999983 | 0.037869 | over |
| GO:0009187 | cyclic nucleotide metabolic process | 0.312094 | 0.999983 | 0.037869 | over |
| GO:0006612 | protein targeting to membrane | 0.312094 | 0.999983 | 0.037869 | over |
| GO:0016849 | phosphorus-oxygen lyase activity | 0.312094 | 0.999983 | 0.037869 | over |
| GO:0006614 | SRP-dependent cotranslational protein targeting to membrane | 0.312094 | 0.999983 | 0.037869 | over |
| GO:0043682 | copper-transporting ATPase activity | 0.312094 | 0.999983 | 0.037869 | over |
| GO:0007606 | sensory perception of chemical stimulus | 0.312094 | 0.999983 | 0.037869 | over |
| GO:0004008 | copper-exporting ATPase activity | 0.312094 | 0.999983 | 0.037869 | over |
| GO:0009190 | cyclic nucleotide biosynthetic process | 0.312094 | 0.999983 | 0.037869 | over |
| GO:0005375 | copper ion transmembrane transporter activity | 0.312094 | 0.999983 | 0.037869 | over |
| GO:0006613 | cotranslational protein targeting to membrane | 0.312094 | 0.999983 | 0.037869 | over |
| GO:0005509 | calcium ion binding | 0.312094 | 0.999983 | 0.037869 | over |
| GO:0045047 | protein targeting to ER | 0.312094 | 0.999983 | 0.037869 | over |
| GO:0005975 | carbohydrate metabolic process | 0.312094 | 0.999983 | 0.038012 | over |
| GO:0034404 | nucleobase, nucleoside and nucleotide biosynthetic process | 0.312094 | 0.999984 | 0.038776 | over |
| GO:0034654 | nucleobase, nucleoside, nucleotide and nucleic acid biosynthetic process | 0.312094 | 0.999984 | 0.038776 | over |
| GO:0006073 | cellular glucan metabolic process | 0.312094 | 0.999987 | 0.039866 | over |
| GO:0044042 | glucan metabolic process | 0.312094 | 0.999987 | 0.039866 | over |
| GO:0019320 | hexose catabolic process | 0.312094 | 0.999989 | 0.042175 | over |
| GO:0006007 | glucose catabolic process | 0.312094 | 0.999989 | 0.042175 | over |
| GO:0046365 | monosaccharide catabolic process | 0.312094 | 0.999989 | 0.042175 | over |
| GO:0009152 | purine ribonucleotide biosynthetic process | 0.313022 | 0.99999 | 0.042841 | over |
| GO:0006066 | alcohol metabolic process | 0.313022 | 0.999991 | 0.044272 | over |
| GO:0009150 | purine ribonucleotide metabolic process | 0.317336 | 0.999993 | 0.045897 | over |
| GO:0044275 | cellular carbohydrate catabolic process | 0.317408 | 0.999993 | 0.047548 | over |
| GO:0043603 | cellular amide metabolic process | 0.318702 | 0.999994 | 0.049031 | over |
