# Supplementary material for: Deciphering the Role of RND Efflux Transporters in Burkholderia cenocepacia
Source: PLoS One. 2011 Apr 19;6(4):e18902. doi: 10.1371/journal.pone.0018902 (PMC3079749; doi:10.1371/journal.pone.0018902)
Supplement: Table S6 — Gene Ontology (GO) terms functional enrichment analysis showing the over or under-representation of up-regulated genes of mutant D4-D9 in comparison to B. cenocepacia J2315 whole genome functional annotation. Only GO terms over- or under- represented with an associated p-value <0.05 are shown. (DOC) [file pone.0018902.s013.doc]

**Table S6. Gene Ontology (GO) terms functional enrichment analysis showing the over or under-representation of up-regulated genes of mutant D4-D9 in comparison to *B. cenocepacia* J2315 whole genome functional annotation.**

| GO terms | Name | FDR | FWER | p-Value | Over/Under |
| --- | --- | --- | --- | --- | --- |
| GO:0048870 | cell motility | 1.94E-09 | 7.47E-09 | 0 | over |
| GO:0044461 | bacterial-type flagellum part | 1.94E-09 | 7.47E-09 | 0 | over |
| GO:0043226 | organelle | 1.94E-09 | 7.47E-09 | 0 | over |
| GO:0042995 | cell projection | 1.94E-09 | 7.47E-09 | 0 | over |
| GO:0019861 | flagellum | 1.94E-09 | 7.47E-09 | 0 | over |
| GO:0009605 | response to external stimulus | 1.94E-09 | 7.47E-09 | 0 | over |
| GO:0006928 | cellular component movement | 1.94E-09 | 7.47E-09 | 0 | over |
| GO:0043229 | intracellular organelle | 1.94E-09 | 7.47E-09 | 0 | over |
| GO:0044460 | flagellum part | 1.94E-09 | 7.47E-09 | 0 | over |
| GO:0044463 | cell projection part | 1.94E-09 | 7.47E-09 | 0 | over |
| GO:0001539 | ciliary or flagellar motility | 1.94E-09 | 7.47E-09 | 0 | over |
| GO:0042330 | taxis | 1.94E-09 | 7.77E-09 | 3.86E-12 | over |
| GO:0006935 | chemotaxis | 1.94E-09 | 7.77E-09 | 3.86E-12 | over |
| GO:0007626 | locomotory behavior | 1.94E-09 | 7.77E-09 | 3.86E-12 | over |
| GO:0007610 | behavior | 1.94E-09 | 7.77E-09 | 3.86E-12 | over |
| GO:0040011 | locomotion | 1.94E-09 | 7.77E-09 | 4.19E-12 | over |
| GO:0003774 | motor activity | 2.04E-09 | 8.65E-09 | 1.06E-11 | over |
| GO:0043064 | flagellum organization | 5.32E-08 | 2.39E-07 | 2.57E-09 | over |
| GO:0030030 | cell projection organization | 1.39E-07 | 6.59E-07 | 6.90E-09 | over |
| GO:0009288 | bacterial-type flagellum | 3.53E-07 | 1.86E-06 | 2.09E-08 | over |
| GO:0009296 | flagellum assembly | 3.53E-07 | 1.86E-06 | 2.09E-08 | over |
| GO:0030031 | cell projection assembly | 1.13E-06 | 6.19E-06 | 6.11E-08 | over |
| GO:0005198 | structural molecule activity | 6.59E-05 | 3.79E-04 | 3.70E-06 | over |
| GO:0007165 | signal transduction | 9.38E-05 | 5.63E-04 | 4.78E-06 | over |
| GO:0042221 | response to chemical stimulus | 1.02E-04 | 6.35E-04 | 5.24E-06 | over |
| GO:0050896 | response to stimulus | 3.46E-04 | 0.00224338 | 2.07E-05 | over |
| GO:0004871 | signal transducer activity | 0.00162346 | 0.0112999 | 9.54E-05 | over |
| GO:0060089 | molecular transducer activity | 0.00162346 | 0.0112999 | 9.54E-05 | over |
| GO:0023046 | signaling process | 0.00263957 | 0.0196023 | 1.56E-04 | over |
| GO:0023060 | signal transmission | 0.00263957 | 0.0196023 | 1.56E-04 | over |
| GO:0022607 | cellular component assembly | 0.00348578 | 0.0266536 | 2.16E-04 | over |
| GO:0004872 | receptor activity | 0.00389824 | 0.0307052 | 2.53E-04 | over |
| GO:0043232 | intracellular non-membrane-bounded organelle | 0.00536918 | 0.0446134 | 3.58E-04 | over |
| GO:0043228 | non-membrane-bounded organelle | 0.00536918 | 0.0446134 | 3.58E-04 | over |
| GO:0009420 | bacterial-type flagellum filament | 0.0252909 | 0.203588 | 9.52E-04 | over |
| GO:0048583 | regulation of response to stimulus | 0.0252909 | 0.203588 | 9.52E-04 | over |
| GO:0016043 | cellular component organization | 0.0330756 | 0.263603 | 0.00168113 | over |
| GO:0009424 | bacterial-type flagellum hook | 0.0555663 | 0.434294 | 0.00279786 | over |
| GO:0009425 | bacterial-type flagellum basal body | 0.0555663 | 0.434294 | 0.00279786 | over |
| GO:0030244 | cellulose biosynthetic process | 0.0555663 | 0.434294 | 0.00279786 | over |
| GO:0030243 | cellulose metabolic process | 0.0555663 | 0.434294 | 0.00279786 | over |
| GO:0051649 | establishment of localization in cell | 0.0617091 | 0.493153 | 0.00394634 | over |
| GO:0009991 | response to extracellular stimulus | 0.0617091 | 0.508283 | 0.00399022 | over |
| GO:0007154 | cell communication | 0.0617091 | 0.508283 | 0.00399022 | over |
| GO:0071496 | cellular response to external stimulus | 0.0617091 | 0.508283 | 0.00399022 | over |
| GO:0031668 | cellular response to extracellular stimulus | 0.0617091 | 0.508283 | 0.00399022 | over |
| GO:0004856 | xylulokinase activity | 0.0793804 | 0.636696 | 0.00548223 | over |
| GO:0005997 | xylulose metabolic process | 0.0793804 | 0.636696 | 0.00548223 | over |
| GO:0004888 | transmembrane receptor activity | 0.0793804 | 0.636696 | 0.00548223 | over |
| GO:0009250 | glucan biosynthetic process | 0.0793804 | 0.636696 | 0.00548223 | over |
| GO:0006011 | UDP-glucose metabolic process | 0.0793804 | 0.636696 | 0.00548223 | over |
| GO:0006525 | arginine metabolic process | 0.0821367 | 0.656388 | 0.00598817 | over |
| GO:0005488 | binding | 0 | 0 | 0.00745898 | under |
| GO:0005215 | transporter activity | 0 | 0 | 0.00791192 | under |
| GO:0005667 | transcription factor complex | 0 | 0 | 0.0085159 | under |
| GO:0003700 | transcription factor activity | 0 | 0 | 0.00889527 | under |
| GO:0007155 | cell adhesion | 0.118118 | 0.797228 | 0.00895218 | over |
| GO:0022610 | biological adhesion | 0.118118 | 0.797228 | 0.00895218 | over |
| GO:0009084 | glutamine family amino acid biosynthetic process | 0.12672 | 0.825129 | 0.0100503 | over |
| GO:0022891 | substrate-specific transmembrane transporter activity | 0 | 0 | 0.010133 | under |
| GO:0022804 | active transmembrane transporter activity | 0 | 0 | 0.0102486 | under |
| GO:0045941 | positive regulation of transcription | 0.15276 | 0.906539 | 0.0131572 | over |
| GO:0010628 | positive regulation of gene expression | 0.15276 | 0.906539 | 0.0131572 | over |
| GO:0010557 | positive regulation of macromolecule biosynthetic process | 0.15276 | 0.906539 | 0.0131572 | over |
| GO:0031328 | positive regulation of cellular biosynthetic process | 0.15276 | 0.906539 | 0.0131572 | over |
| GO:0016563 | transcription activator activity | 0.15276 | 0.906539 | 0.0131572 | over |
| GO:0006537 | glutamate biosynthetic process | 0.15276 | 0.906539 | 0.0131572 | over |
| GO:0009891 | positive regulation of biosynthetic process | 0.15276 | 0.906539 | 0.0131572 | over |
| GO:0043234 | protein complex | 0 | 0 | 0.014032 | under |
| GO:0009064 | glutamine family amino acid metabolic process | 0.160157 | 0.920512 | 0.0154186 | over |
| GO:0044428 | nuclear part | 0 | 0 | 0.0154313 | under |
| GO:0022857 | transmembrane transporter activity | 0 | 0 | 0.0158911 | under |
| GO:0006560 | proline metabolic process | 0.160157 | 0.93477 | 0.016878 | over |
| GO:0045184 | establishment of protein localization | 0.160157 | 0.936797 | 0.017351 | over |
| GO:0015031 | protein transport | 0.160157 | 0.936797 | 0.017351 | over |
| GO:0044451 | nucleoplasm part | 0 | 0 | 0.0174054 | under |
| GO:0009987 | cellular process | 0.160157 | 0.938109 | 0.0177071 | over |
| GO:0042254 | ribosome biogenesis | 0.160157 | 0.942896 | 0.0179192 | over |
| GO:0003735 | structural constituent of ribosome | 0.160157 | 0.942896 | 0.0179192 | over |
| GO:0015935 | small ribosomal subunit | 0.160157 | 0.956608 | 0.0180493 | over |
| GO:0045935 | positive regulation of nucleobase, nucleoside, nucleotide and nucleic acid metabolic process | 0.160157 | 0.956608 | 0.0180493 | over |
| GO:0010604 | positive regulation of macromolecule metabolic process | 0.160157 | 0.956608 | 0.0180493 | over |
| GO:0051173 | positive regulation of nitrogen compound metabolic process | 0.160157 | 0.956608 | 0.0180493 | over |
| GO:0048518 | positive regulation of biological process | 0.160157 | 0.956608 | 0.0180493 | over |
| GO:0009893 | positive regulation of metabolic process | 0.160157 | 0.956608 | 0.0180493 | over |
| GO:0048522 | positive regulation of cellular process | 0.160157 | 0.956608 | 0.0180493 | over |
| GO:0031325 | positive regulation of cellular metabolic process | 0.160157 | 0.956608 | 0.0180493 | over |
| GO:0006605 | protein targeting | 0.160157 | 0.956608 | 0.0180493 | over |
| GO:0017111 | nucleoside-triphosphatase activity | 0.160157 | 0.957892 | 0.0185232 | over |
| GO:0006631 | fatty acid metabolic process | 0.00529945 | 0.0145028 | 0.0196404 | under |
| GO:0022613 | ribonucleoprotein complex biogenesis | 0.164864 | 0.963194 | 0.0198337 | over |
| GO:0044085 | cellular component biogenesis | 0.171285 | 0.969005 | 0.0218699 | over |
| GO:0004040 | amidase activity | 0.1877 | 0.979791 | 0.0235824 | over |
| GO:0006071 | glycerol metabolic process | 0.1877 | 0.979791 | 0.0235824 | over |
| GO:0016462 | pyrophosphatase activity | 0.191572 | 0.982233 | 0.025617 | over |
| GO:0016818 | hydrolase activity, acting on acid anhydrides, in phosphorus-containing anhydrides | 0.195712 | 0.985242 | 0.0271009 | over |
| GO:0016817 | hydrolase activity, acting on acid anhydrides | 0.195712 | 0.985242 | 0.0271009 | over |
| GO:0009432 | SOS response | 0.208894 | 0.991948 | 0.0297128 | over |
| GO:0016998 | cell wall macromolecule catabolic process | 0.208894 | 0.991948 | 0.0297128 | over |
| GO:0000049 | tRNA binding | 0.208894 | 0.991948 | 0.0297128 | over |
| GO:0044459 | plasma membrane part | 0.208894 | 0.991948 | 0.0297128 | over |
| GO:0046903 | secretion | 0.208894 | 0.99231 | 0.0304954 | over |
| GO:0009306 | protein secretion | 0.208894 | 0.99231 | 0.0304954 | over |
| GO:0032940 | secretion by cell | 0.208894 | 0.99231 | 0.0304954 | over |
| GO:0009428 | bacterial-type flagellum basal body, distal rod, P ring | 0.341285 | 0.999991 | 0.0309735 | over |
| GO:0030092 | regulation of flagellum assembly | 0.341285 | 0.999991 | 0.0309735 | over |
| GO:0004043 | L-aminoadipate-semialdehyde dehydrogenase activity | 0.341285 | 0.999991 | 0.0309735 | over |
| GO:0003992 | N2-acetyl-L-ornithine:2-oxoglutarate 5-aminotransferase activity | 0.341285 | 0.999991 | 0.0309735 | over |
| GO:0019357 | nicotinate nucleotide biosynthetic process | 0.341285 | 0.999991 | 0.0309735 | over |
| GO:0005416 | cation:amino acid symporter activity | 0.341285 | 0.999991 | 0.0309735 | over |
| GO:0016714 | oxidoreductase activity, acting on paired donors, with incorporation or reduction of molecular oxygen, reduced pteridine as one donor, and incorporation of one atom of oxygen | 0.341285 | 0.999991 | 0.0309735 | over |
| GO:0032101 | regulation of response to external stimulus | 0.341285 | 0.999991 | 0.0309735 | over |
| GO:0009267 | cellular response to starvation | 0.341285 | 0.999991 | 0.0309735 | over |
| GO:0031344 | regulation of cell projection organization | 0.341285 | 0.999991 | 0.0309735 | over |
| GO:0004515 | nicotinate-nucleotide adenylyltransferase activity | 0.341285 | 0.999991 | 0.0309735 | over |
| GO:0004055 | argininosuccinate synthase activity | 0.341285 | 0.999991 | 0.0309735 | over |
| GO:0030257 | type III protein secretion system complex | 0.341285 | 0.999991 | 0.0309735 | over |
| GO:0003904 | deoxyribodipyrimidine photo-lyase activity | 0.341285 | 0.999991 | 0.0309735 | over |
| GO:0003913 | DNA photolyase activity | 0.341285 | 0.999991 | 0.0309735 | over |
| GO:0005887 | integral to plasma membrane | 0.341285 | 0.999991 | 0.0309735 | over |
| GO:0040012 | regulation of locomotion | 0.341285 | 0.999991 | 0.0309735 | over |
| GO:0005283 | sodium:amino acid symporter activity | 0.341285 | 0.999991 | 0.0309735 | over |
| GO:0030694 | bacterial-type flagellum basal body, rod | 0.341285 | 0.999991 | 0.0309735 | over |
| GO:0006562 | proline catabolic process | 0.341285 | 0.999991 | 0.0309735 | over |
| GO:0018298 | protein-chromophore linkage | 0.341285 | 0.999991 | 0.0309735 | over |
| GO:0031226 | intrinsic to plasma membrane | 0.341285 | 0.999991 | 0.0309735 | over |
| GO:0006030 | chitin metabolic process | 0.341285 | 0.999991 | 0.0309735 | over |
| GO:0080135 | regulation of cellular response to stress | 0.341285 | 0.999991 | 0.0309735 | over |
| GO:0004568 | chitinase activity | 0.341285 | 0.999991 | 0.0309735 | over |
| GO:0003842 | 1-pyrroline-5-carboxylate dehydrogenase activity | 0.341285 | 0.999991 | 0.0309735 | over |
| GO:0060491 | regulation of cell projection assembly | 0.341285 | 0.999991 | 0.0309735 | over |
| GO:0004371 | glycerone kinase activity | 0.341285 | 0.999991 | 0.0309735 | over |
| GO:0044087 | regulation of cellular component biogenesis | 0.341285 | 0.999991 | 0.0309735 | over |
| GO:0031667 | response to nutrient levels | 0.341285 | 0.999991 | 0.0309735 | over |
| GO:0009427 | bacterial-type flagellum basal body, distal rod, L ring | 0.341285 | 0.999991 | 0.0309735 | over |
| GO:0031669 | cellular response to nutrient levels | 0.341285 | 0.999991 | 0.0309735 | over |
| GO:0050795 | regulation of behavior | 0.341285 | 0.999991 | 0.0309735 | over |
| GO:0003961 | O-acetylhomoserine aminocarboxypropyltransferase activity | 0.341285 | 0.999991 | 0.0309735 | over |
| GO:0050568 | protein-glutamine glutaminase activity | 0.341285 | 0.999991 | 0.0309735 | over |
| GO:0008834 | di-trans,poly-cis-decaprenylcistransferase activity | 0.341285 | 0.999991 | 0.0309735 | over |
| GO:0006032 | chitin catabolic process | 0.341285 | 0.999991 | 0.0309735 | over |
| GO:0042594 | response to starvation | 0.341285 | 0.999991 | 0.0309735 | over |
| GO:0006282 | regulation of DNA repair | 0.341285 | 0.999991 | 0.0309735 | over |
| GO:0004505 | phenylalanine 4-monooxygenase activity | 0.341285 | 0.999991 | 0.0309735 | over |
| GO:0004657 | proline dehydrogenase activity | 0.341285 | 0.999991 | 0.0309735 | over |
| GO:0080134 | regulation of response to stress | 0.341285 | 0.999991 | 0.0309735 | over |
| GO:0050920 | regulation of chemotaxis | 0.341285 | 0.999991 | 0.0309735 | over |
| GO:0019843 | rRNA binding | 0.346072 | 0.999993 | 0.0326897 | over |
| GO:0044422 | organelle part | 0.0343499 | 0.0984938 | 0.0334151 | under |
| GO:0015405 | P-P-bond-hydrolysis-driven transmembrane transporter activity | 0.0343499 | 0.12881 | 0.0340605 | under |
| GO:0015399 | primary active transmembrane transporter activity | 0.0343499 | 0.12881 | 0.0340605 | under |
| GO:0050662 | coenzyme binding | 0.0343499 | 0.12881 | 0.0345031 | under |
| GO:0044446 | intracellular organelle part | 0.0343499 | 0.12881 | 0.0346072 | under |
| GO:0030529 | ribonucleoprotein complex | 0.347733 | 0.999994 | 0.0347343 | over |
| GO:0006026 | aminoglycan catabolic process | 0.349387 | 0.999996 | 0.0363988 | over |
| GO:0019400 | alditol metabolic process | 0.349387 | 0.999996 | 0.0363988 | over |
| GO:0019321 | pentose metabolic process | 0.349387 | 0.999996 | 0.0363988 | over |
| GO:0006558 | L-phenylalanine metabolic process | 0.351301 | 0.999997 | 0.0369843 | over |
| GO:0044267 | cellular protein metabolic process | 0.351301 | 0.999997 | 0.0372353 | over |
| GO:0006412 | translation | 0.354036 | 0.999997 | 0.0396516 | over |
| GO:0015075 | ion transmembrane transporter activity | 0.0346092 | 0.137251 | 0.0399699 | under |
| GO:0042626 | ATPase activity, coupled to transmembrane movement of substances | 0.0419214 | 0.181222 | 0.0426974 | under |
| GO:0043492 | ATPase activity, coupled to movement of substances | 0.0419214 | 0.181222 | 0.0426974 | under |
| GO:0009279 | cell outer membrane | 0.362831 | 0.999998 | 0.0427062 | over |
| GO:0033279 | ribosomal subunit | 0.369214 | 0.999999 | 0.0436007 | over |
| GO:0022892 | substrate-specific transporter activity | 0.0484962 | 0.225936 | 0.0466119 | under |
| GO:0051234 | establishment of localization | 0.0484962 | 0.235037 | 0.0485636 | under |
| GO:0006810 | transport | 0.0484962 | 0.235037 | 0.0485636 | under |
| GO:0043648 | dicarboxylic acid metabolic process | 0.382342 | 0.999999 | 0.0492039 | over |
