# Supplementary material for: Deciphering the Role of RND Efflux Transporters in Burkholderia cenocepacia
Source: PLoS One. 2011 Apr 19;6(4):e18902. doi: 10.1371/journal.pone.0018902 (PMC3079749; doi:10.1371/journal.pone.0018902)
Supplement: Table S7 — Gene Ontology (GO) terms functional enrichment analysis showing the over or under-representation of down-regulated genes of mutant D4–D9 in comparison to B. cenocepacia J2315 whole genome functional annotation. Only GO terms over- or under- represented with an associated p-value <0.05 are shown. (DOC) [file pone.0018902.s014.doc]

**Table S7. Gene Ontology (GO) terms functional enrichment analysis showing the over or under-representation of down-regulated genes of mutant D4-D9 in comparison to *B. cenocepacia* J2315 whole genome functional annotation.**

| GO terms | Name | FDR | FWER | p-Value | Over/Under |
| --- | --- | --- | --- | --- | --- |
| GO:0009289 | pilus | 0.113684 | 0.068841 | 4.94E-04 | over |
| GO:0006091 | generation of precursor metabolites and energy | 0.113684 | 0.097202 | 7.95E-04 | over |
| GO:0005488 | binding | 0 | 0 | 8.82E-04 | under |
| GO:0009056 | catabolic process | 0.113684 | 0.106714 | 9.49E-04 | over |
| GO:0004022 | alcohol dehydrogenase (NAD) activity | 0.113684 | 0.127735 | 9.60E-04 | over |
| GO:0044282 | small molecule catabolic process | 0.113684 | 0.13248 | 0.001034 | over |
| GO:0008568 | microtubule-severing ATPase activity | 0.165068 | 0.281205 | 0.001406 | over |
| GO:0016566 | specific transcriptional repressor activity | 0.165068 | 0.281205 | 0.001406 | over |
| GO:0003987 | acetate-CoA ligase activity | 0.165068 | 0.281205 | 0.001406 | over |
| GO:0006096 | glycolysis | 0.178929 | 0.358063 | 0.002269 | over |
| GO:0044260 | cellular macromolecule metabolic process | 0 | 0 | 0.002298 | under |
| GO:0004586 | ornithine decarboxylase activity | 0.178929 | 0.58161 | 0.004113 | over |
| GO:0008783 | agmatinase activity | 0.178929 | 0.58161 | 0.004113 | over |
| GO:0006574 | valine catabolic process | 0.178929 | 0.606183 | 0.00474 | over |
| GO:0009083 | branched chain family amino acid catabolic process | 0.178929 | 0.606183 | 0.00474 | over |
| GO:0006550 | isoleucine catabolic process | 0.178929 | 0.606183 | 0.00474 | over |
| GO:0006552 | leucine catabolic process | 0.178929 | 0.606183 | 0.00474 | over |
| GO:0019319 | hexose biosynthetic process | 0.178929 | 0.618325 | 0.004941 | over |
| GO:0006094 | gluconeogenesis | 0.178929 | 0.618325 | 0.004941 | over |
| GO:0046903 | secretion | 0.178929 | 0.624549 | 0.00505 | over |
| GO:0032940 | secretion by cell | 0.178929 | 0.624549 | 0.00505 | over |
| GO:0009306 | protein secretion | 0.178929 | 0.624549 | 0.00505 | over |
| GO:0016020 | membrane | 0.178929 | 0.641683 | 0.005265 | over |
| GO:0001882 | nucleoside binding | 0.011966 | 0.017798 | 0.005411 | under |
| GO:0001883 | purine nucleoside binding | 0.011966 | 0.017798 | 0.005691 | under |
| GO:0030554 | adenyl nucleotide binding | 0.011966 | 0.017798 | 0.005691 | under |
| GO:0046364 | monosaccharide biosynthetic process | 0.178929 | 0.672496 | 0.006206 | over |
| GO:0017076 | purine nucleotide binding | 0.011966 | 0.017798 | 0.006822 | under |
| GO:0046165 | alcohol biosynthetic process | 0.178929 | 0.702688 | 0.006921 | over |
| GO:0006139 | nucleobase, nucleoside, nucleotide and nucleic acid metabolic process | 0.012247 | 0.021216 | 0.007128 | under |
| GO:0019320 | hexose catabolic process | 0.178929 | 0.7219 | 0.007352 | over |
| GO:0046365 | monosaccharide catabolic process | 0.178929 | 0.7219 | 0.007352 | over |
| GO:0006007 | glucose catabolic process | 0.178929 | 0.7219 | 0.007352 | over |
| GO:0016052 | carbohydrate catabolic process | 0.178929 | 0.730997 | 0.007753 | over |
| GO:0004491 | methylmalonate-semialdehyde dehydrogenase (acylating) activity | 0.178929 | 0.78815 | 0.008022 | over |
| GO:0042618 | poly-hydroxybutyrate metabolic process | 0.178929 | 0.78815 | 0.008022 | over |
| GO:0051790 | short-chain fatty acid biosynthetic process | 0.178929 | 0.78815 | 0.008022 | over |
| GO:0008106 | alcohol dehydrogenase (NADP+) activity | 0.178929 | 0.78815 | 0.008022 | over |
| GO:0042619 | poly-hydroxybutyrate biosynthetic process | 0.178929 | 0.78815 | 0.008022 | over |
| GO:0046358 | butyrate biosynthetic process | 0.178929 | 0.78815 | 0.008022 | over |
| GO:0006560 | proline metabolic process | 0.178929 | 0.821966 | 0.00942 | over |
| GO:0044275 | cellular carbohydrate catabolic process | 0.178929 | 0.822595 | 0.009532 | over |
| GO:0043170 | macromolecule metabolic process | 0.015267 | 0.033792 | 0.009611 | under |
| GO:0090304 | nucleic acid metabolic process | 0.015267 | 0.033792 | 0.009807 | under |
| GO:0044248 | cellular catabolic process | 0.178929 | 0.835681 | 0.010425 | over |
| GO:0051649 | establishment of localization in cell | 0.178929 | 0.842691 | 0.011227 | over |
| GO:0019867 | outer membrane | 0.178929 | 0.842691 | 0.011227 | over |
| GO:0015977 | carbon fixation | 0.178929 | 0.854846 | 0.011358 | over |
| GO:0071704 | organic substance metabolic process | 0.178929 | 0.854846 | 0.011358 | over |
| GO:0019643 | reductive tricarboxylic acid cycle | 0.178929 | 0.854846 | 0.011358 | over |
| GO:0005975 | carbohydrate metabolic process | 0.178929 | 0.859417 | 0.011757 | over |
| GO:0016051 | carbohydrate biosynthetic process | 0.178929 | 0.863274 | 0.012549 | over |
| GO:0009063 | cellular amino acid catabolic process | 0.178929 | 0.866633 | 0.012925 | over |
| GO:0009310 | amine catabolic process | 0.178929 | 0.866633 | 0.012925 | over |
| GO:0006595 | polyamine metabolic process | 0.195815 | 0.904827 | 0.013041 | over |
| GO:0006596 | polyamine biosynthetic process | 0.195815 | 0.904827 | 0.013041 | over |
| GO:0030163 | protein catabolic process | 0.195815 | 0.904827 | 0.013041 | over |
| GO:0046164 | alcohol catabolic process | 0.206641 | 0.920777 | 0.014165 | over |
| GO:0006525 | arginine metabolic process | 0.206641 | 0.924664 | 0.014923 | over |
| GO:0008152 | metabolic process | 0.021012 | 0.051225 | 0.015608 | under |
| GO:0000166 | nucleotide binding | 0.028151 | 0.081094 | 0.016384 | under |
| GO:0003676 | nucleic acid binding | 0.028151 | 0.081094 | 0.016568 | under |
| GO:0006090 | pyruvate metabolic process | 0.218702 | 0.947726 | 0.017824 | over |
| GO:0032787 | monocarboxylic acid metabolic process | 0.218702 | 0.948866 | 0.018287 | over |
| GO:0046395 | carboxylic acid catabolic process | 0.218702 | 0.949473 | 0.018342 | over |
| GO:0016054 | organic acid catabolic process | 0.218702 | 0.949473 | 0.018342 | over |
| GO:0034637 | cellular carbohydrate biosynthetic process | 0.218702 | 0.950763 | 0.018889 | over |
| GO:0046459 | short-chain fatty acid metabolic process | 0.240502 | 0.967683 | 0.019082 | over |
| GO:0019605 | butyrate metabolic process | 0.240502 | 0.967683 | 0.019082 | over |
| GO:0005524 | ATP binding | 0.028976 | 0.096588 | 0.019647 | under |
| GO:0032559 | adenyl ribonucleotide binding | 0.028976 | 0.096588 | 0.019647 | under |
| GO:0006006 | glucose metabolic process | 0.246024 | 0.971923 | 0.020225 | over |
| GO:0019538 | protein metabolic process | 0.03204 | 0.113401 | 0.021069 | under |
| GO:0044267 | cellular protein metabolic process | 0.037148 | 0.154236 | 0.022374 | under |
| GO:0006551 | leucine metabolic process | 0.261798 | 0.980428 | 0.022667 | over |
| GO:0006573 | valine metabolic process | 0.261798 | 0.980428 | 0.022667 | over |
| GO:0032553 | ribonucleotide binding | 0.037148 | 0.154236 | 0.022786 | under |
| GO:0032555 | purine ribonucleotide binding | 0.037148 | 0.154236 | 0.022786 | under |
| GO:0065007 | biological regulation | 0.041022 | 0.177403 | 0.024314 | under |
| GO:0006549 | isoleucine metabolic process | 0.272499 | 0.985471 | 0.025409 | over |
| GO:0009081 | branched chain family amino acid metabolic process | 0.272499 | 0.985471 | 0.025409 | over |
| GO:0004033 | aldo-keto reductase activity | 0.28926 | 0.990983 | 0.026061 | over |
| GO:0015662 | ATPase activity, coupled to transmembrane movement of ions, phosphorylative mechanism | 0.28926 | 0.990983 | 0.026061 | over |
| GO:0044262 | cellular carbohydrate metabolic process | 0.28926 | 0.991017 | 0.026104 | over |
| GO:0042995 | cell projection | 0.28926 | 0.992002 | 0.02746 | over |
| GO:0050789 | regulation of biological process | 0.051757 | 0.228514 | 0.027697 | under |
| GO:0006066 | alcohol metabolic process | 0.28926 | 0.992213 | 0.027859 | over |
| GO:0019318 | hexose metabolic process | 0.295416 | 0.993654 | 0.028376 | over |
| GO:0032196 | transposition | 0.059575 | 0.275394 | 0.029348 | under |
| GO:0006553 | lysine metabolic process | 0.295416 | 0.993948 | 0.029825 | over |
| GO:0050794 | regulation of cellular process | 0.059575 | 0.280049 | 0.030259 | under |
| GO:0006950 | response to stress | 0.298211 | 0.99465 | 0.031574 | over |
| GO:0006554 | lysine catabolic process | 0.29827 | 0.9954 | 0.032842 | over |
| GO:0009068 | aspartate family amino acid catabolic process | 0.29827 | 0.9954 | 0.032842 | over |
| GO:0006766 | vitamin metabolic process | 0.06519 | 0.317472 | 0.033011 | under |
| GO:0018883 | caprolactam metabolic process | 0.314239 | 0.997325 | 0.033901 | over |
| GO:0019384 | caprolactam catabolic process | 0.314239 | 0.997325 | 0.033901 | over |
| GO:0006020 | inositol metabolic process | 0.314239 | 0.997325 | 0.033901 | over |
| GO:0004803 | transposase activity | 0.06519 | 0.346263 | 0.034331 | under |
| GO:0006767 | water-soluble vitamin metabolic process | 0.06519 | 0.346263 | 0.034331 | under |
| GO:0006313 | transposition, DNA-mediated | 0.06519 | 0.346263 | 0.034331 | under |
| GO:0005996 | monosaccharide metabolic process | 0.314239 | 0.997513 | 0.035454 | over |
| GO:0016070 | RNA metabolic process | 0.068903 | 0.372875 | 0.035703 | under |
| GO:0018874 | benzoate metabolic process | 0.314239 | 0.997678 | 0.036407 | over |
| GO:0005945 | 6-phosphofructokinase complex | 0.604189 | 0.999999 | 0.037611 | over |
| GO:0008662 | 1-phosphofructokinase activity | 0.604189 | 0.999999 | 0.037611 | over |
| GO:0050269 | coniferyl-aldehyde dehydrogenase activity | 0.604189 | 0.999999 | 0.037611 | over |
| GO:0045491 | xylan metabolic process | 0.604189 | 0.999999 | 0.037611 | over |
| GO:0004030 | aldehyde dehydrogenase [NAD(P)+] activity | 0.604189 | 0.999999 | 0.037611 | over |
| GO:0008443 | phosphofructokinase activity | 0.604189 | 0.999999 | 0.037611 | over |
| GO:0010410 | hemicellulose metabolic process | 0.604189 | 0.999999 | 0.037611 | over |
| GO:0050100 | methylitaconate delta-isomerase activity | 0.604189 | 0.999999 | 0.037611 | over |
| GO:0010383 | cell wall polysaccharide metabolic process | 0.604189 | 0.999999 | 0.037611 | over |
| GO:0043711 | pilus organization | 0.604189 | 0.999999 | 0.037611 | over |
| GO:0008776 | acetate kinase activity | 0.604189 | 0.999999 | 0.037611 | over |
| GO:0043754 | dihydrolipoyllysine-residue (2-methylpropanoyl)transferase activity | 0.604189 | 0.999999 | 0.037611 | over |
| GO:0003872 | 6-phosphofructokinase activity | 0.604189 | 0.999999 | 0.037611 | over |
| GO:0009297 | pilus assembly | 0.604189 | 0.999999 | 0.037611 | over |
| GO:0050182 | phosphate butyryltransferase activity | 0.604189 | 0.999999 | 0.037611 | over |
| GO:0008792 | arginine decarboxylase activity | 0.604189 | 0.999999 | 0.037611 | over |
| GO:0003879 | ATP phosphoribosyltransferase activity | 0.604189 | 0.999999 | 0.037611 | over |
| GO:0045493 | xylan catabolic process | 0.604189 | 0.999999 | 0.037611 | over |
| GO:0009064 | glutamine family amino acid metabolic process | 0.604189 | 1 | 0.039472 | over |
| GO:0008168 | methyltransferase activity | 0.078921 | 0.425576 | 0.041756 | under |
| GO:0016405 | CoA-ligase activity | 0.625708 | 1 | 0.042527 | over |
| GO:0045184 | establishment of protein localization | 0.625708 | 1 | 0.043937 | over |
| GO:0015031 | protein transport | 0.625708 | 1 | 0.043937 | over |
| GO:0044424 | intracellular part | 0.080171 | 0.441972 | 0.044928 | under |
| GO:0006099 | tricarboxylic acid cycle | 0.629277 | 1 | 0.048519 | over |
| GO:0046356 | acetyl-CoA catabolic process | 0.629277 | 1 | 0.048519 | over |
| GO:0006633 | fatty acid biosynthetic process | 0.629277 | 1 | 0.048717 | over |
