# Supplementary material for: Deciphering the Role of RND Efflux Transporters in Burkholderia cenocepacia
Source: PLoS One. 2011 Apr 19;6(4):e18902. doi: 10.1371/journal.pone.0018902 (PMC3079749; doi:10.1371/journal.pone.0018902)
Supplement: Table S8 — Schematic representation of data obtained from PM (from PM11 to PM20) analyses of B. cenocepacia strain J2315, D4, D9 and D4–D9. *IC50 was calculated on the basis of the kinetic curves obtained on the four different concentrations of each chemical compound and it was defined as the well or fraction of a well at which the area of kinetic curve is at half of its maximal value over the concentration series. **IC50 is reported only for compounds under which the difference between the areas of the kinetic curves of wild-type and mutant strain was over 15000 units in at least one of the concentrations tested. (DOC) [file pone.0018902.s015.doc]

**Table S8. Schematic representation of data obtained from PM (from PM11 to PM20) analyses of *B. cenocepacia* strain J2315, D4, D9 and D4-D9.**

| **Substrate/Chemical** | **Panels (wells)** | **Mode of action** | **IC50*** | | | |
| --- | --- | --- | --- | --- | --- | --- |
| **Antibiotics** | | | **J2315** | **D4**** | **D9**** | **D4-D9**** |
| Minocycline | PM11 (C9-C12) | Protein synthesis. 30S ribosomal subunit. tetracycline | 3.67 | >4.40 | >4.40 | >4.40 |
| Tylosin | PM13(H9-H12) | Protein synthesis. 50S ribosomal subunit. macrolide | 3.6 | 2.25 |  | 2.09 |
| Josamycin | PM19(A1-A4) | Protein synthesis. 50S ribosomal subunit. macrolide | 4.01 | 2.85 |  | 2.6 |
| Puromycin | PM15 (F9-F12) | Protein synthesis. 30S ribosomal subunit. premature chanin termination | >4.40 | 2.65 |  | 2.62 |
| Chloramphenicol | PM11-(F1-F4) | Protein synthesis. amphenicol | 3.67 | 1.98 |  | 1.87 |
| Chloramphenicol | PM14 (F1-F4) | Protein synthesis. amphenicol | >4.40 | 2.76 |  | 2.73 |
| Thiamphenicol | PM18(a9-A12) | Protein synthesis. amphenicol | 3.66 | 1.35 |  | 1.28 |
| Fusidic acid | PM15(C5-C8) | Protein synthesis. elongation factor | 1.65 | 0.63 |  | 0.62 |
| Nalidixic acid | PM11(E9-E12) | DNA topoisomerase. quinolone antibiotics | 3.79 | 2.55 |  | 1.99 |
| Oxolinic acid | PM13(B9-B12) | DNA topoisomerase. quinolone antibiotics | >4.40 | 3.9 |  | 3.57 |
| Cinoxacin | PM16(D9-D12) | DNA topoisomerase. quinolone antibiotics | >4.40 | 3.52 |  | 3.03 |
| **DNA intercalators** | | | | | | |
| Acriflavine | PM14(A1-A4) | DNA intercalator. inhibits RNA synthesis | >4.40 | 3.93 |  | 3.92 |
| Proflavine | PM20(D1-D4) | DNA intercalator. inhibits RNA synthesis | >4.40 | 4.09 |  | 2.89 |
| **Drugs** | | | | | | |
| Ketoprofen | PM18(A1-A4) | Biofilm inhibitor. anti-capsule agent. prostaglandin syntetase inhibitor | 3.51 | 2.71 |  | 2.22 |
| Lidocaine | PM18(D9-D12) | Ion channel inhibitor. Na+. anaesthetic | 2.89 | 0.68 |  | 0.89 |
| Orphenadrine | PM20(B1-B4) | Anti-cholinergic | 4.21 |  |  | 2.84 |
| Propranolol | PM20(B5-B8) | Beta-adrenergic blocker | 4.34 | 3.57 |  | 3.14 |
| Atropine | PM20(C5-C8) | Acetylcholine receptor. antagonist | 3.67 |  |  | 3.16 |
| Amitriptyline | PM20(A1-A4) | Membrane. transport. tricyclic antidepressant (TCA) | >4.40 |  |  | 3.46 |
| **Fungicides** | | | | | | |
| Dichlofluanid | PM16(C1-C4) | Fungicide. phenylsulphamide | >4.40 | 3.47 |  |  |
| Captan | PM20(G1-G4) | Multisite. carbamate. fungicide | 4.36 |  |  | 2.21 |
| **Detergents** | | | | | | |
| Niaproof | PM17(E1-E4) | Membrane. detergent. anionic | 2.51 | 1.51 |  | 1.27 |
| Lauryl sulfobetaine | PM19(G1-G4) | Membrane. detergent. zwitterionic | 1.5 | 0.6 |  | 0.6 |
| **Toxic anions** | | | | | | |
| Sodium dichromate | PM14(D9-D12) | Toxic anion. SO4 analog | 1.62 |  |  | 0.84 |
| Sodium metasilicate | PM18(E1-E4) | Toxic anion | 3.5 | 3.00 |  | 2.8 |
| **Respiration/Ionophores/Uncouplers** | | | | | | |
| Pentachlorophenol | PM18(C9-C12) | Respiration. ionophore. H+ | >4.40 |  |  | 3.89 |
| FCCP | PM19(E1-E4) | Respiration. ionophore. H+ | 4.14 | 0.67 |  | 0.64 |
| Menadione | PM14(G9-G12) | Respiration. uncoupler | >4.40 |  |  | 4.29 |
| Tetrazolium violet | PM20(B9-B12) | Respiration. uncoupler | >4.40 | 4.17 |  | 4.08 |
| **Oxidizing agents** | | | | | | |
| Plumbagin | PM18(h9-H12) | Oxidizing agent | >4.40 | 3.91 |  | 3.93 |
| D.L-Thioctic acid | PM19(E5-E9) | Oxidizing agent | 3.86 | 2.96 |  | 2.58 |
| Ornidazole | PM20(C9-C12) | Nitro compound. oxidizing agent. DNA damage | 4.35 |  |  | 3.62 |

*IC50 was calculated on the basis of the kinetic curves obtained on the four different concentrations of each chemical compound and it was defined as the well or fraction of a well at which the area of kinetic curve is at half of its maximal value over the concentration series.

**IC50 is reported only for compounds under which the difference between the areas of the kinetic curves of wild-type and mutant strain was over 15000 units in at least one of the concentrations tested.
