# Supplementary material for: Deciphering the Role of RND Efflux Transporters in Burkholderia cenocepacia
Source: PLoS One. 2011 Apr 19;6(4):e18902. doi: 10.1371/journal.pone.0018902 (PMC3079749; doi:10.1371/journal.pone.0018902)
Supplement: Table S9 — List of genes differentially regulated in B. cenocepacia strains D4, D9, D4–D9 versus J2315 known to be also controlled by AHL-based quorum sensing. (DOC) [file pone.0018902.s016.doc]

**Table S9. List of genes differentially regulated in *B. cenocepacia* strains D4, D9, D4-D9 *versus* J2315 known to be also controlled by AHL-based quorum sensing.**

| **Gene no.** | **Description** | **Change in gene expression (log_2_ fold change)** | | | **Reference** |
| --- | --- | --- | --- | --- | --- |
|  |  | **D4 vs J2315** | **D9 vs J2315** | **D4-D9 vs J2315** |  |
| BCAL0010 | phenylalanine-4-hydroxylase | - | - | 2.02 | Subsin *et al*. 2007.  J. Bacteriol. 189:968-979 |
| BCAL0170 | conserved hypothetical protein | -1.93 | - | - | Subsin *et al*., 2007 |
| BCAL0562 | putative negative regulator of flagellin synthesis (anti-sigma-28 factor) | 2.81 | - | 1.34 | Chambers *et al*. 2006. BMC Microbiol. 6:104 |
| BCAL0812 | sigma 54 modulation protein | -1.42 | -1.50 | -1.15 | Subsin *et al*., 2007 |
| BCAL0854 | GntR family regulatory protein | - | - | 1.02 | Subsin *et al*., 2007 |
| BCAL1040 | glycosyl transferases group 1 protein | - | - | 0.59 | Subsin *et al*., 2007 |
| BCAL1651 | LexA repressor | - | 1.07 | 1.38 | Subsin *et al*., 2007 |
| BCAL2464 | short chain dehydrogenase | - | - | -0.83 | Subsin *et al*., 2007 |
| BCAL2757 | superoxide dismutase SodB | - | - | 0.66 | Riedel *et al*. 2003. Electroph. 24:740-750 |
| BCAL2799 | putative carbohydrate kinase | - | - | 0.65 | Subsin *et al*., 2007 |
| BCAL2818 | putative sugar kinase protein | - | - | 0.82 | Subsin *et al*., 2007 |
| BCAL3506 | flagellar motor switch protein FliM | 2.58 | - | - | Subsin *et al*., 2007 |
| BCAM0300 | putative metallo-beta-lactamase family protein | - | - | -1.66 | Subsin *et al*., 2007 |
| BCAM0901 | putative AMP nucleosidase | - | - | 0.60 | Subsin *et al*., 2007 |
| BCAM1110 | putative MFS family sugar transporter protein | 2.58 | - | - | Subsin *et al*., 2007 |
| BCAM1502 | conserved hypothetical protein | - | - | -1.78 | Chambers *et al*., 2006 |
| BCAS0293 | nematocidal protein AidA | - | - | 1.84 | Chambers *et al*., 2006 Riedel *et al*., 2003 |
